# Plakophilin 1 suppresses keratinocyte innate immune responses through DExD/H helicases

**DOI:** 10.1038/s41423-026-01462-3

**Published:** 2026-08-19

**Authors:** René Keil, Danny Misiak, Stefan Hüttelmaier, Frederik Tellkamp, Marcus Krüger, Mechthild Hatzfeld

**Affiliations:** 1 Charles Tanford Protein Research Center, Martin Luther University Halle, Institute of Molecular Medicine, Halle, Germany; 2 Institute for Genetics University of Cologne, Cologne, Germany; 3 Cluster of Excellence for Cellular Stress Responses and Age-related Diseases (CECAD) University of Cologne, Cologne, Germany; 4 Center for Molecular Medicine Cologne (CMMC), Cologne, Germany

**Keywords:** Inflammation, RIG-I-like receptors, DExD/H-box helicases, Plakophilin 1, RIG-I-like receptors, Cell signalling, Acute inflammation

## Abstract

The innate immune response is a first line of defense that allows a rapid reaction to pathogens as well as molecules released from damaged cells. It relies on pathogen-sensing receptors, many of which are nucleic acid-binding proteins, including RNA helicases. Plakophilin 1 (PKP1) is a desmosomal protein essential for strong intercellular adhesion and epithelial barrier integrity. Loss-of-function mutations in PKP1 cause ectodermal dysplasia–skin fragility syndrome (EDSFS), which is characterized by skin fragility, chronic inflammation, and recurrent infections. Here, we report that PKP1, whose role in barrier formation is to function as a critical regulator of innate immune responses. PKP1 sequesters a subset of dsRNAs that sense DExD/H-box RNA helicases—DDX1, DDX3X, DDX21, and DHX15. This limits their ability to activate the MDA5-MAVS signaling axis and prevents excessive activation of IRF3- and NFκB-driven gene expression, including the synthesis of proinflammatory cytokines, mainly IFN-β1, IL6, and TNFα. The inhibitory role of PKP1 is erased during innate immune responses by dsRNA cues, inducing proteasomal PKP1 degradation. This releases sequestered helicases, enabling dsRNA sensing and a rapid inflammatory response. Collectively, these findings identify PKP1 as a key gatekeeper that prevents exaggerated inflammation in quiescent keratinocytes, which is supported by excessive inflammation in EDSFS upon PKP1 loss. In healthy keratinocytes with proinflammatory traits, PKP1 is rapidly degraded, allowing the activation of the MDA5-MAVS signaling cascade by helicases to initiate inflammation. Hence, PKP1 combines two complementary functions essential for epidermal immune homeostasis: it provides a physical barrier preventing substance entry and suppresses innate immune responses in keratinocytes.

## Introduction

Innate immunity serves as the first line of defense against microbial pathogens, orchestrating the activation of adaptive immune responses and tissue repair. This system utilizes a conserved repertoire of receptors specific for microbial components to generate rapid inflammatory responses [1]. It spans virtually all tissues and involves cells of both epithelial and hematopoietic origin [2]. Innate immunity comprises several components, including physical barriers and pathogen-sensing receptors and their effectors. Epithelial cells in barrier tissues perform critical immune functions by detecting, restricting, and often directly eliminating extrinsic pathogens [3].

The skin provides an effective physical barrier between the internal and external environment that prevents the entry of potentially harmful substances and microorganisms [4]. The outermost layer of the epidermis mainly consists of keratinocytes (KCs) that are organized into a multilayered epithelium. Their tight cohesion enables the formation of a physical barrier. Dysfunction of this barrier is associated with inflammatory skin diseases such as psoriasis and atopic dermatitis, as well as tumor development. Desmosomes are intercellular junctions that provide resistance to mechanical forces by linking the intermediate filaments of neighboring cells [5–8]. Reduced intercellular cohesion between KCs is accompanied by local and/or systemic inflammation [9–11], a phenomenon underscored by severe genetic and infectious diseases in which pathogens target desmosomal components. The protein plakophilin 1 (PKP1) is a major component of desmosomes in stratified epithelia [12]. Loss-of-function mutations in PKP1 cause the rare autosomal recessive disorder “ectodermal dysplasia skin fragility syndrome” (EDSFS; OMIM 604536) [13], which is characterized by skin fragility, chronic perioral inflammation (cheilitis), and palmoplantar hyperkeratosis. In addition, growth retardation, generalized neonatal erythema, recurrent skin and systemic infections, and pruritus have been described [14]. These patients exhibit a reduced number and size of desmosomes in the epidermis, which is also observed in PKP1 knockout (KO) mice [15]. PKP1 is required for desmosomes to adopt a highly stable Ca^2+^-independent hyperadhesive state [12, 16, 17]. In addition to its structural role, PKP1 is involved in the regulation of several cellular processes, including mRNA metabolism, translation, cell growth, proliferation, migration and the DNA damage response [12, 18–20]. Notably, PKP1 is associated with various RNA-binding proteins (RBPs), several of which are known to modulate cytokine stability [21, 22] and govern immune responses. In prostatic tumors, PKP1 deficiency is correlated with immune cell recruitment and upregulation of cytokine expression [23]. These data suggest that, in addition to its role in barrier formation, PKP1 may actively modulate inflammatory responses.

KCs are central mediators of innate immunity, highlighting the interdependence of physical and immunological barriers. They express several pattern recognition receptors (PRRs), including Toll-like receptors (TLRs) and RIG-like receptors (retinoic acid-inducible gene-I-like receptors, RLRs). Upon sensing pathogen-associated molecular patterns (PAMPs) or damage-associated molecular patterns (DAMPs), these PRRs trigger signaling cascades that activate transcription factors such as AP-1, NFκB and interferon regulatory factors (IRFs). This leads to the induction of proinflammatory cytokines, type I interferons (IFNs), chemokines, and antimicrobial proteins that are essential for rapid and efficient host defense and contribute to tissue repair and homeostasis. However, excessive or prolonged inflammation can lead to nonhealing chronic wounds, fibrosis, autoimmunity or cancer [24–27].

Many PRRs are nucleic acid-binding proteins, including DExD/H-box family RNA helicases that sense viral double-stranded RNA (dsRNA) [28]. Canonical RLRs include RIG-I (*DDX58*), melanoma differentiation-associated protein 5 (MDA-5; *IFIH1*), and laboratory of genetics and physiology 2 (LGP2; *DHX58*). Upon activation, RIG-I and MDA5 interact with mitochondrial antiviral signaling (MAVS), which recruits downstream targets to promote the nuclear translocation of IRF3 and NFκB, thereby activating the transcription of innate immune response genes [29]. While the absence of both RIG-I and MDA5 completely abrogates IFN production, indicating their essential role in evoking type I IFN responses to RNA viruses [30], LGP2 functions as a versatile modulator. It can act as a negative regulator of RIG-I and MDA5 [31–33] while also facilitating the MDA5-driven response to dsRNA [34, 35]. Additional helicases further fine-tune this defense: DDX3 enhances dsRNA sensing at early stages of infection [36]. DHX15 binds to PAMP RNAs to facilitate signaling to MAVS [37, 38], and the DDX1/DDX21/DHX36 complex seems to act as a primary sensor in parallel to RIG-I and MDA-5 [39].

In this study, we demonstrated that PKP1 directly regulates inflammatory responses in keratinocytes. We show that PKP1 forms complexes with the RNA helicases DDX1, DDX3X, DDX21, and DHX15, resulting in their sequestration and reduced activation of the MDA5-MAVS signaling axis. Upon dsRNA stimulation, PKP1 undergoes proteasomal degradation, triggering the disassembly of these complexes and unleashing helicase-mediated immune activation. This dynamic regulation enables a transient inflammatory response while preventing excessive or prolonged signaling. In summary, we propose that PKP1 serves as a molecular checkpoint that integrates epithelial integrity with innate immune control by modulating helicase functions.

## Results

### Loss of PKP1 increases the secretion of proinflammatory cytokines in murine keratinocytes

**Fig. 1:**
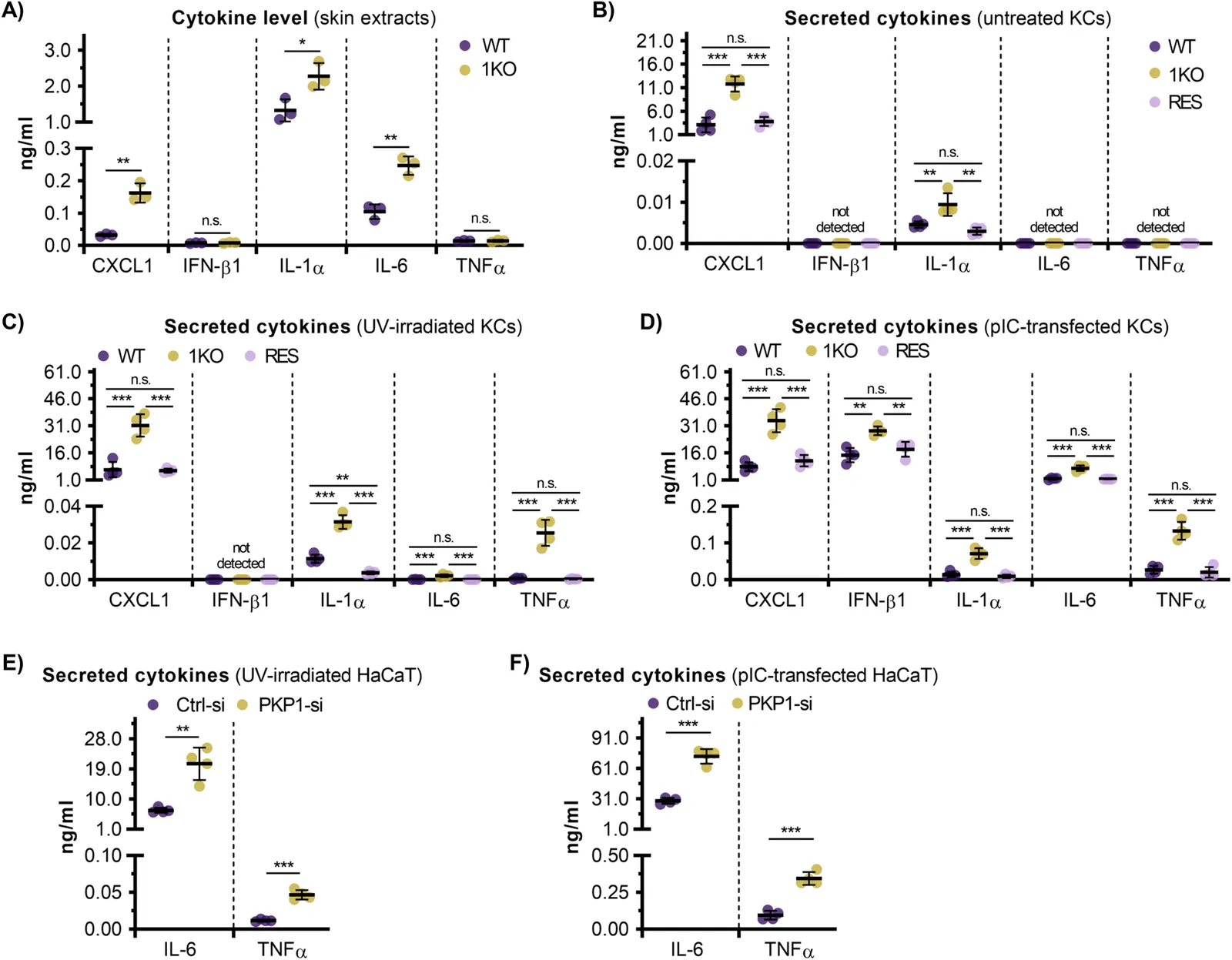
Loss of PKP1 increases the secretion of proinflammatory cytokines. **A** Protein extracts from the back skin of newborn PKP1-KO (1KO) mice and their wild-type (WT) littermates were analyzed for the expression of CXCL1, IFN-β1, IL-1α, IL-6 and TNFα by ELISA. The data represent the mean cytokine concentrations ± SDs from three WT and three 1KO animals. **B** WT, PKP1-KO (1KO) and PKP1-rescue (RES) KCs were cultured for 24 h in high-calcium medium, and the levels of secreted CXCL1, IFN-β1, IL-1α, IL-6 and TNFα were quantified by ELISA. The data represent the mean cytokine concentrations ± SDs; n = 4. WT, 1KO and RES KCs were exposed to UVB irradiation (**C**) or transfected with pIC (**D**). After 24 h, the culture supernatants were collected, and the levels of secreted CXCL1, IFN-β1, IL-1α, IL-6 and TNFα were quantified by ELISA. The data represent the mean cytokine concentrations ± SDs; *n* = 4. Human HaCaT cells were transfected with control or PKP1-directed siRNAs and subsequently exposed to UVB irradiation (**E**) or transfected with pIC (**F**). After 24 h, the culture supernatants were collected, and the levels of secreted IL-6 and TNFα were quantified by ELISA. The data represent the mean cytokine concentrations ± SDs; *n* = 4. Statistical significance was determined by t tests (**A, E, F**) or one-way ANOVA with Tukey’s multiple comparison test (**B–D**). n.s. = not significant; * *p* < 0.05; ** *p* < 0.01; *** *p* < 0.001

PKP1-KO mice develop skin lesions within a few hours, after which they rapidly regress and die within 24 h after birth [15]. To address whether dysregulated inflammatory responses might contribute to this phenotype, we initially quantified the levels of the proinflammatory cytokines C-X-C motif chemokine ligand 1 (CXCL1), interferon-β1 (IFN-β1), interleukin-1α (IL-1α), IL-6 and tumor necrosis factor α (TNFα) in protein extracts derived from the skin of newborn wild-type (WT) and PKP1-KO mice (Fig. 1A). IFN-β1 and TNFα were barely detectable and essentially unaltered in skin extracts derived from WT and PKP1-KO mice. In contrast, PKP1-KO mice presented significantly increased levels of CXCL1, IL-1α and IL-6, suggesting that the loss of PKP1 induces an inflammatory response in the skin. However, this study does not distinguish between immune cell recruitment caused by barrier defects and the intrinsic activation of inflammatory signaling. To further investigate whether PKP1 interferes more directly with inflammatory responses in KCs, we used spontaneously immortalized WT, PKP1-KO and PKP1-KO KCs expressing PKP1-GFP (PKP1-rescue KCs) (Fig. S1A) [16]. Under basal conditions (24 h in high-calcium medium without exogenous stimulation), KCs secreted CXCL1 and IL-1α but failed to produce detectable levels of IFN-β1, IL-6 and TNFα (Fig. 1B). Notably, the secretion of both CXCL1 and IL-1α was substantially greater in PKP1-KO KCs than in both WT and PKP1-rescued KCs. These data indicate that PKP1 deficiency leads to aberrant, cell-intrinsic inflammatory activation in KCs.

We next investigated whether exogenous stimulation further altered the inflammatory profiles of WT, PKP1-KO, and rescue KCs (experimental design, Fig. S1B). Exposure to UV radiation is a highly relevant physiological factor that causes inflammatory reactions in KCs [40, 41]. Accordingly, we quantified cytokine secretion from WT, PKP1-KO and PKP1-rescued KCs 24 h post-UVB irradiation (Figs. 1C and S1C). UVB irradiation induced or increased the secretion of CXCL1, IL-1α, IL-6, and TNFα, while IFN-β1 remained undetectable in all the cell lines. Notably, these proinflammatory cytokines were secreted at the highest level in PKP1-KO-KC cells. These findings indicate that PKP1 attenuates the inflammatory response in KCs. Mechanistically, UVB irradiation can damage self-noncoding RNAs, which subsequently trigger cytokine production in KCs [42]. To determine the impact of PKP1 on dsRNA-driven immune activation, KCs were transfected with polyinosinic-polycytidylic acid (pIC). Comparable and high transfection efficiencies were confirmed via Cy3-labeled pIC (Fig. S1D), ensuring that subsequent differences in cytokine secretion were not due to differential pIC uptake. Stimulation with pIC strongly induced the secretion of CXCL1, IFN-β1, IL-1α, IL-6 and TNFα. Consistent with the hypothesis that PKP1 restricts dsRNA-driven signaling, compared with PKP1-KO KCs, WT and PKP1-rescue KCs secreted significantly lower amounts of these proinflammatory cytokines (Figs. 1D and S1E).

To analyze whether the PKP1-dependent modulation of cytokine production is preserved in human cells, we used HaCaT keratinocytes treated with either control or PKP1-directed siRNAs (see Fig. S1F for knockdown efficiency). Consistent with our murine data, UV irradiation induced the secretion of IL6 and TNFα, which was further amplified in PKP1-depleted cells (Fig. 1E). Similarly, pIC transfection increased IL6 and TNFα secretion, and again, PKP1 knockdown amplified this effect (Fig. 1F). Collectively, these findings indicate that PKP1 acts as a negative regulator of cytokine secretion in both mouse KCs and human KCs.

**Fig. 2:**
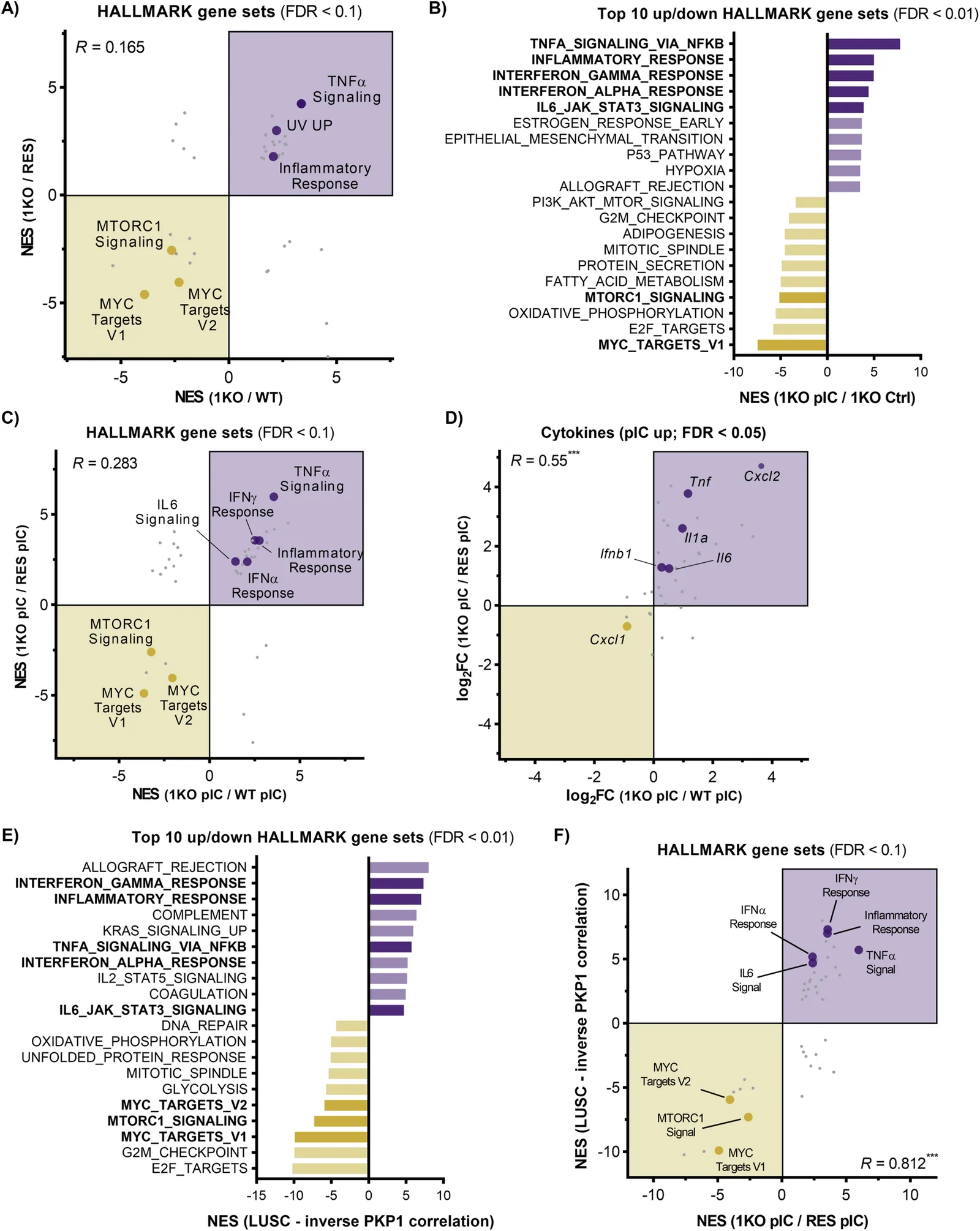
Globally, PKP1 affects inflammatory and innate immune gene expression. **A** XY plot of normalized enrichment scores (NESs) for mouse ortholog hallmark gene sets determined by gene set enrichment analysis (GSEA). GSEA was performed using gene lists ranked by fold change in untreated PKP1-KO (1KO) versus WT KCs (x-axis) or untreated 1KO versus PKP1-rescue (RES) KCs (y-axis). **B** Top up- and downregulated mouse ortholog hallmark gene sets identified by GSEA using a gene list ranked by fold change between pIC-transfected and mock-transfected 1KO KCs. **C** XY plot of the NES for mouse ortholog hallmark gene sets comparing pIC-transfected 1KO versus WT KCs (x-axis) and pIC-transfected 1KO versus RES KCs (y-axis). **D** XY plot of log2-fold changes in cytokine-encoding genes in pIC-transfected 1KO versus WT KCs (x-axis) and pIC-transfected 1KO versus RES KCs (y-axis) based on RNA-seq data. Only cytokines significantly upregulated upon pIC treatment in 1KO KCs were included. **E** Top up- and downregulated hallmark gene sets identified by GSEA using a gene list ranked by inverse correlation coefficients between PKP1 and significantly correlated genes (*p* ≤ 0.05) from the “Mixed Lung Squamous Cell Carcinoma v32” TCGA dataset. **F** XY plot of the NES for hallmark gene sets comparing pIC-transfected 1KO KCs versus RES KCs (x-axis), and gene sets enriched in LUSC samples with inverse PKP1 correlation (y-axis)

To assess whether PKP1 affects inflammatory responses globally, we performed an RNA-seq analysis (LC Sciences; Texas) of unstimulated and pIC-stimulated WT, PKP1-KO and PKP1-rescued KCs. Gene set enrichment analyses (GSEAs) of data from unstimulated KCs revealed significant upregulation of proinflammatory gene expression patterns upon PKP1 knockout compared with those in WT and rescue KCs (Figs. 2A and S2A). This analysis also confirmed the published effect of PKP1 on c-MYC and mTOR signaling [43, 44], validating our transcriptomic approach. Strikingly, the top five hallmark gene sets upregulated in pIC-stimulated PKP1-KO KCs were exclusively proinflammatory gene sets (Fig. 2B). The significant upregulation of these genes and the expression of specific pIC-induced cytokines in PKP1-KO cells, as well as their subsequent restoration in rescue KCs (Figs. 2C, D and S2B, C), indicate that PKP1 attenuates the inflammatory transcriptome, likely through transcriptional or posttranscriptional control.

While PKP1 expression is primarily restricted to stratified squamous epithelia, it is frequently overexpressed in lung squamous cell carcinoma (LUSC) [45, 46]. To evaluate whether the immunosuppressive role of PKP1 extends to human disease, we analyzed transcriptomic data from 550 patient-derived LUSC samples (TCGA dataset, “Mixed Lung Squamous Cell Carcinoma v32”). We performed GSEA using a gene list ranked by the inverse correlation coefficients between PKP1 expression and all significantly correlated genes (*p* ≤ 0.05). Strikingly, the top 10 gene sets induced in the PKP1-low cohort matched the five proinflammatory hallmark pathways identified in our pIC-stimulated PKP1-KO KCs (compare Fig. 2B, E). These data directly substantiate that PKP1 loss triggers an uncoupled inflammatory state. Conversely, this clinical analysis also confirmed the robust positive correlation between PKP1 expression and c-MYC/mTOR signaling. By demonstrating that the absence of PKP1 mimics active immune stimulation while its presence aligns with growth-promoting pathways, these data independently validate our computational model and align with established regulatory axes [43, 44].

A comparative analysis of significantly PKP1-correlated gene sets in LUSC samples versus differentially regulated sets in pIC-treated PKP1-KO KCs (Figs. 2F and S2D) revealed a shared transcriptomic signature. Collectively, these data demonstrate that the loss of PKP1 leads to global dysregulation of inflammatory signaling, a pattern consistently observed across both murine keratinocyte models and human LUSC datasets.

### PKP1 affects the transcriptional control of proinflammatory cytokines

**Fig. 3:**
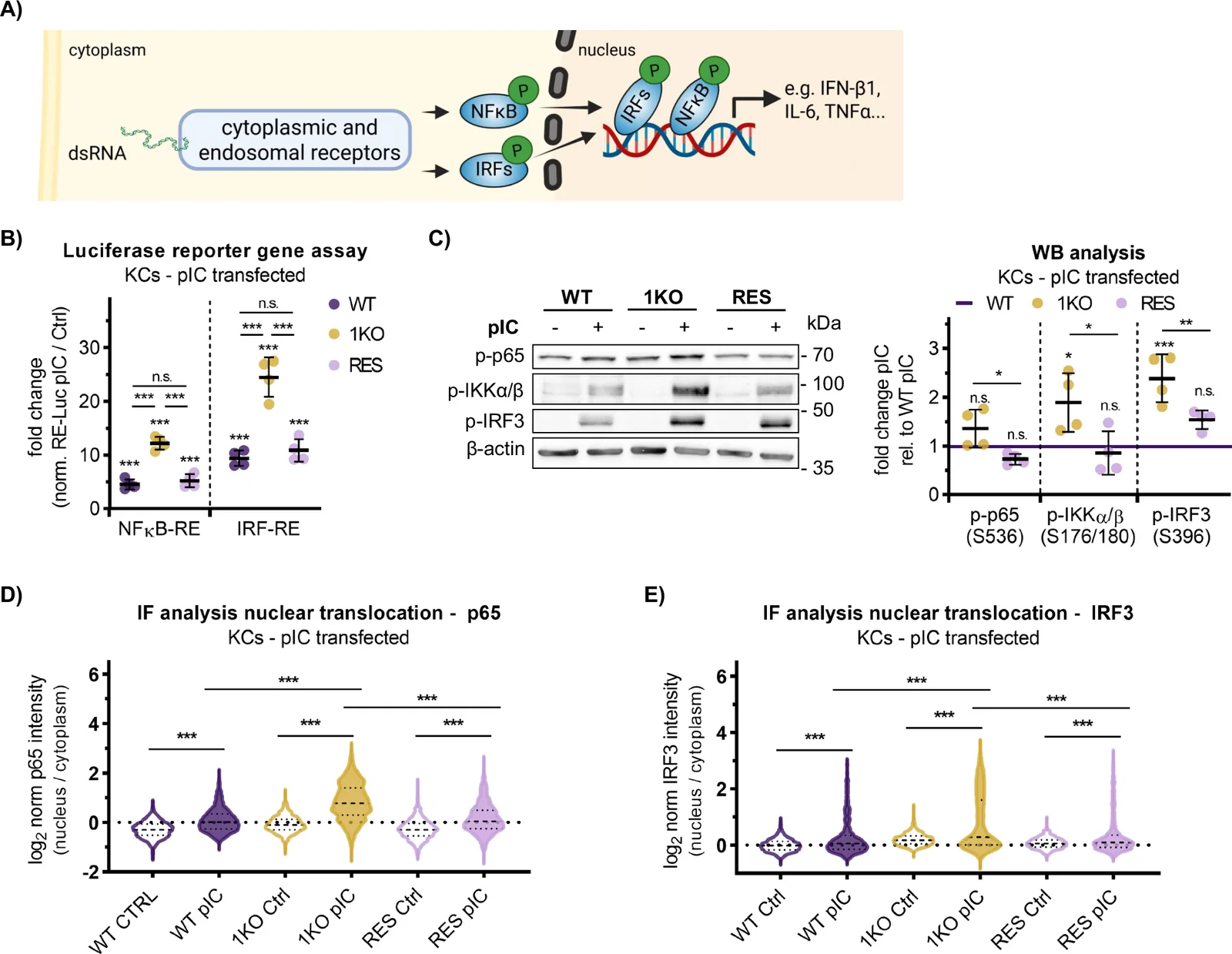
PKP1 represses NFκB and IRF transcription factor activity. **A** Schematic overview of NFκB and IRF transcription factor activation by dsRNA. Created with BioRender. **B** Luciferase reporter assays measuring NFκB and IRF transcription factor activity in pIC-transfected WT, PKP1-KO (1KO) and PKP1-rescue (RES) KCs. Response element-dependent firefly luciferase activity (RE-Luc) was normalized to Renilla luciferase luminescence. The data represent the mean fold changes ± SDs of normalized RE-Luc activity in pIC-transfected KCs relative to that in mock-transfected KCs; *n* = 4. **C** Western blot analysis of WT, 1KO and RES KCs 6 h after mock or pIC transfection. Left: representative immunoblots of phosphorylated p65, IKKα/β and IRF3. β-actin served as a loading control. Right: Densitometric quantification of phospho-protein levels normalized to that of β-actin. Data show the fold changes ± SDs in pIC-transfected 1KO and RES KCs relative to pIC-transfected WT KCs; *n* = 4. Immunofluorescence analysis of mock- and pIC-transfected WT, 1KO and RES KCs to determine the nuclear accumulation of p65 (**D**) and IRF3 (**E**). Data are shown as log2-transformed nuclear-to-cytoplasmic fluorescence intensity ratios and represent >900 cells per condition pooled from three independent experiments. Representative images are shown in Figure S3. Statistical significance was determined using one-way ANOVA with Tukey’s multiple comparison test (**B, C**) or a nonparametric Kruskal‒Wallis test (**D, E**); n.s. = not significant; * *p* < 0.05; ** *p* < 0.01; *** *p* < 0.001

The absence of PKP1 promoted proinflammatory signaling under basal conditions, which was substantially enhanced by pIC stimulation. Our GSEA of the RNA-seq data suggested that both NFκB- and IRF-driven gene expression [Fig. 3A, [47]] are impaired by PKP1 and enhanced in PKP1-KO keratinocytes. To validate the role of PKP1 in the transcriptional activation of both pathways, we first explored luciferase reporters containing NFKB- or IRF-response elements (REs) in WT, PKP1-KO and PKP1-rescued KCs after pIC stimulation (Fig. 3B). Stimulation of KCs with pIC significantly increased NFκB-RE-Luc activity and, to a greater extent, IRF-RE-Luc activity. Consistent with our previous results, compared with WT and PKP1-rescued KCs, PKP1-KO KCs exhibited significantly higher NFκB- and IRF-RE-Luc activity. This was further evaluated by monitoring the activation of the respective transcription factors via their phosphorylation (Fig. 3C). However, pIC transfection led to only a modest increase in the phosphorylation of p65 at S536 across all genotypes. Since p65 activation is governed by multiple phosphorylation sites and regulatory steps, we also examined the phosphorylation of the upstream kinases IKKα/β at S176/180. Unlike p65-S536 phosphorylation, IKKα/β phosphorylation was strongly induced by pIC treatment, with a significantly greater increase observed in PKP1-KO cells. To resolve the discrepancy between the subtle p65 phosphorylation and the robust induction seen in our luciferase reporter assays, we analyzed the nuclear translocation of p65 as a definitive surrogate for its activation [48]. In unstimulated WT, PKP1-KO, and PKP1-rescue KCs, p65 was predominantly cytoplasmic, with no marked differences in the nuclear-to-cytoplasmic ratio between genotypes. After pIC transfection, p65 translocated into the nucleus in all KC cell lines. Notably, compared with those in WT and PKP1-rescue cells, the nuclear pool in KC-KO cells was significantly greater, as demonstrated by an increased nuclear-to-cytoplasmic ratio (Figs. 3D and S2A). These findings are fully consistent with those of the NFκB reporter gene assays and support the notion that PKP1 serves as a negative regulator of this signaling pathway.

In addition to activating canonical NFκB-driven inflammation, PRR engagement also activates IRF3 to coordinate the innate immune response. Phosphorylated IRF3 (p-IRF3) was not detectable in unstimulated KCs but was readily detected in pIC-stimulated cells (Fig. 3C). In accordance with the results of the reporter gene assay, compared with PKP1-KO KCs, PKP1-expressing KCs presented significantly lower levels of p-IRF3, suggesting that PKP1 attenuates the dsRNA-dependent activation of IRF3. Moreover, the nuclear translocation of IRF3 upon pIC stimulation was also regulated in a PKP1-dependent manner (Figs. 3E and S3B). To determine whether this regulatory mechanism is conserved, we examined p65 and IRF3 phosphorylation in human HaCaT cells (Fig. S3C). Consistent with our findings in murine KCs, pIC transfection triggered increases in both p65 and IRF3 phosphorylation, an effect that was markedly more pronounced in PKP1-depleted cells. Taken together, these results demonstrate a conserved inhibitory role of PKP1, establishing it as a crucial gatekeeper that restricts both NFκB-mediated inflammation and IRF3-driven signaling.

**Fig. 4:**
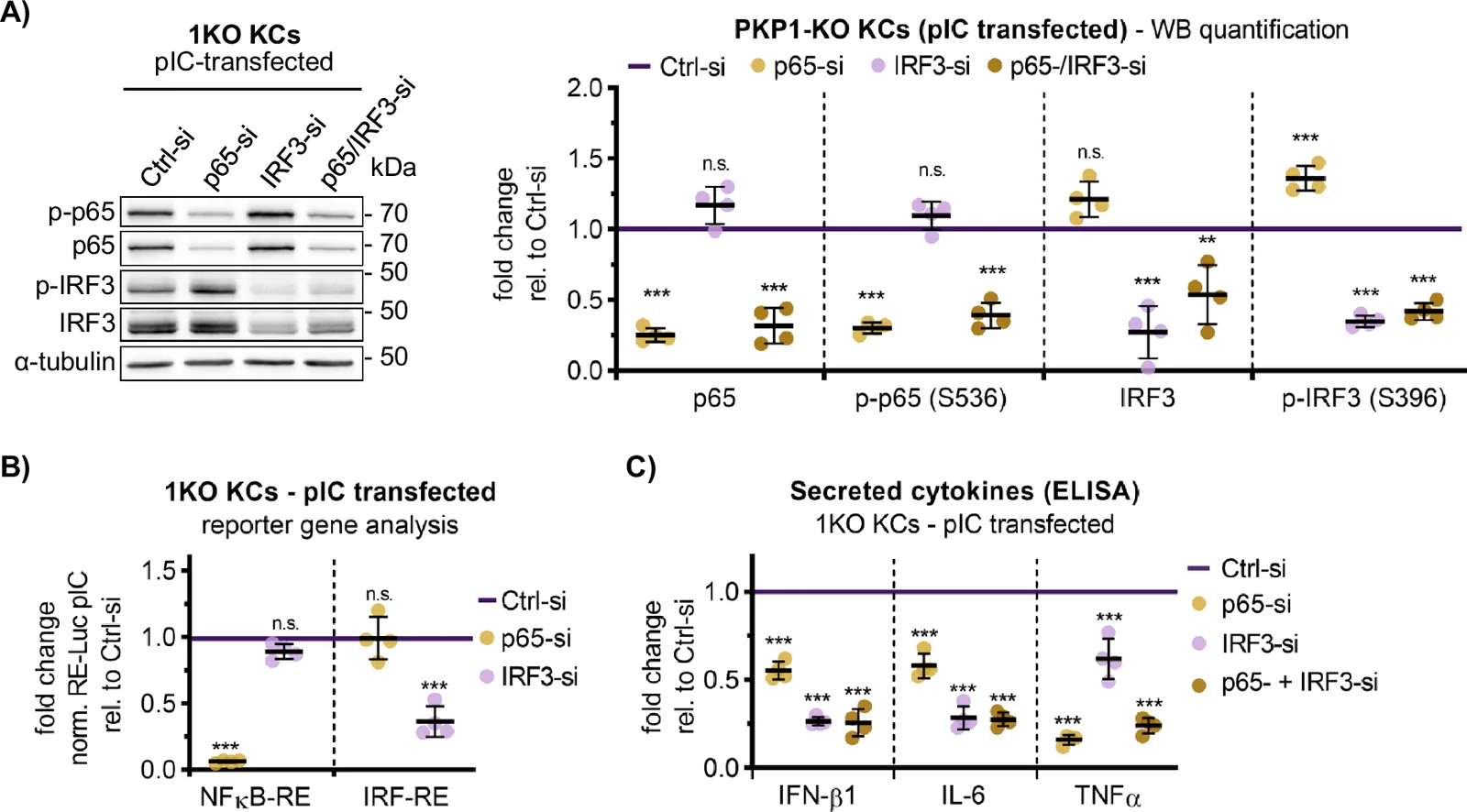
PKP1-dependent proinflammatory cytokine secretion depends on NFκB-p65 and IRF3. **A** Western blot analysis of PKP1-KO (1KO) KCs transfected with the indicated siPools prior to pIC stimulation. Left: representative immunoblots of phosphorylated and total p65 and IRF3. α-Tubulin served as a loading control. Right: Densitometric quantification of total and phosphorylated protein levels normalized to that of α-tubulin. The data represent the fold changes ± SDs relative to control siPool-transfected, pIC-stimulated 1KO KCs; *n* = 4. **B** Luciferase reporter assays measuring NFκB and IRF transcription factor activity in siPool-transfected and pIC-stimulated 1KO KCs. RE-Luc activity was normalized to Renilla luciferase luminescence. The data are presented as the mean fold changes ± SDs relative to control siPool-transfected, pIC-stimulated 1KO KCs; *n* = 4. **C** 1KO KCs transfected with the indicated siPools were stimulated with pIC. After 24 h, the levels of secreted IFN-β1, IL-6 and TNFα were quantified by ELISA. The data represent the fold changes ± SDs relative to control siPool-transfected, pIC-stimulated 1KO KCs; *n* = 4. Statistical significance was determined by one-way ANOVA with Tukey’s multiple comparison test. n.s. = not significant; ** *p* < 0.01; *** *p* < 0.001

Next, we analyzed whether p65 and IRF3 are mandatory for the dsRNA-dependent expression of IFN-β1, IL-6 and TNFα in KC from PKP1-KO mice. Therefore, we utilized siRNA pools to deplete p65 and IRF3 in PKP1-KO KCs prior to pIC transfection. The knockdown of p65 and IRF3 was validated by WB analysis, in which antibodies were used to detect phosphorylated or total amounts of the respective transcription factors (Fig. 4A). The amounts of total and phosphorylated p65 and IRF3 were reduced by more than 50%. To address the functional consequences and specificity of p65 and IRF3 depletion, NFκB- and IRF-RE-dependent luciferase reporter gene assays were performed (Fig. 4B). The knockdown of p65 almost completely abolished NFκB-RE-Luc activity but had no effect on IRF-RE-Luc activity. IRF3 depletion strongly reduced pIC-dependent IRF-RE-Luc activity but did not affect NFκB-RE-Luc activity. The remaining activity of the IRF-RE reporter could be due to either residual amounts of IRF3 or the dsRNA-dependent activation of other IRF family transcription factors, such as IRF1, IRF6 and IRF7, which are also expressed in our KC cell lines (Fig. S3D). Consistently, knockdown of either p65 or IRF3, as well as their combined depletion, significantly reduced the secretion of IFN-β1, IL-6 and TNFα in PKP1-KO KCs upon pIC transfection (Fig. 4C). While IRF3 depletion affected mainly the secretion of IFN-β1 and IL-6, TNFα secretion was dependent mainly on p65. Synergistic effects caused by the combined depletion of p65 and IRF3 were not detected. Collectively, these data demonstrate that the heightened immune response in the absence of PKP1 is driven by parallel, factor-specific axes, thereby reinforcing the pivotal inhibitory role of PKP1 as a dual gatekeeper that restricts both NF-κB- and IRF3-dependent downstream cytokine production.

### PKP1 regulates MDA5/MAVS signaling to control the transcription of proinflammatory cytokines

**Fig. 5:**
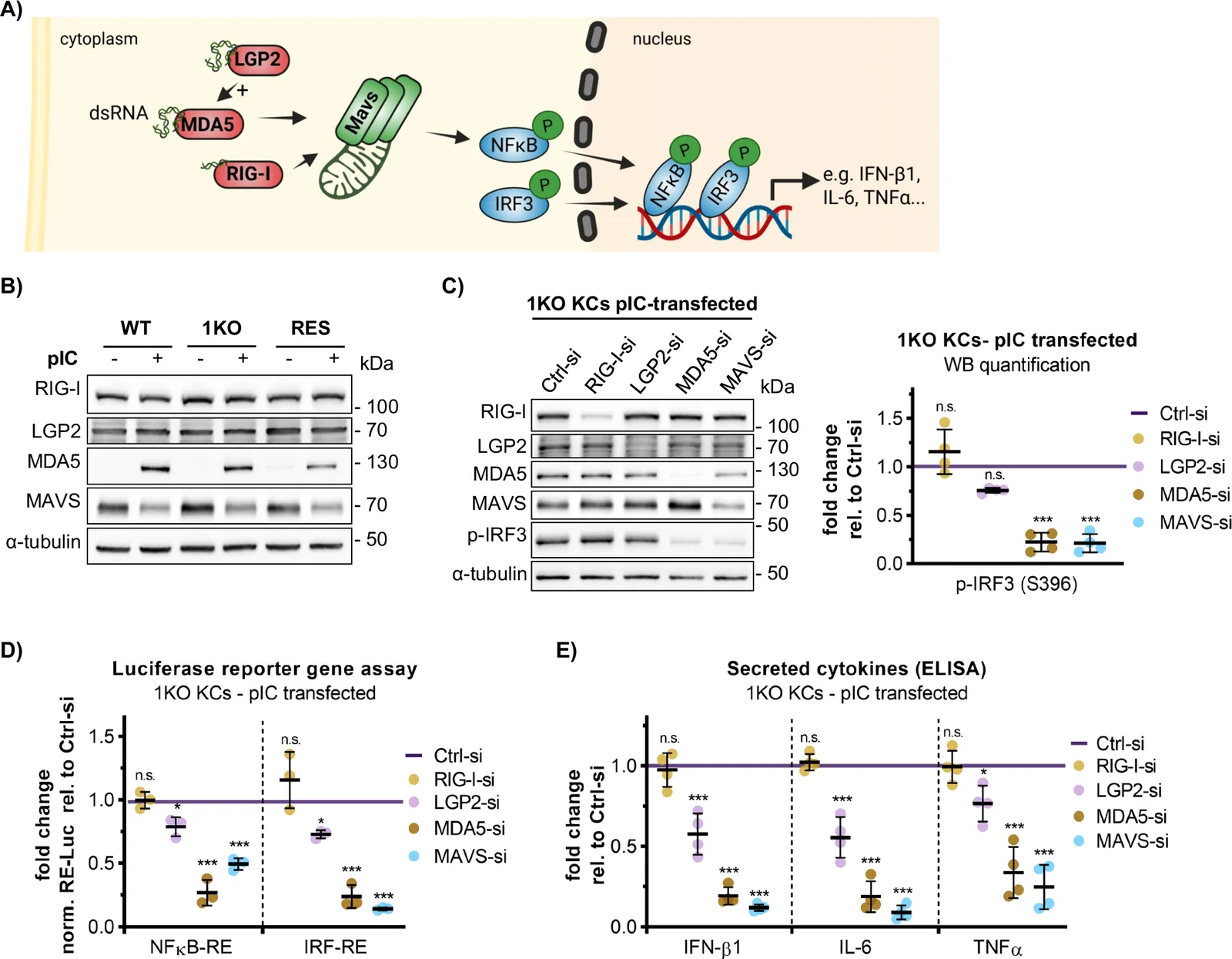
PKP1 affects the transcriptional control of proinflammatory cytokines through MDA5/MAVS signaling. **A** Schematic overview of RLR signaling responsible for dsRNA-dependent activation of NFkB and IRF3. Created in BioRender. **B** Western blot analysis of mock- and pIC-transfected WT, PKP1-KO (1KO) and PKP1-rescue (RES) KCs. Representative immunoblots showing proteins involved in dsRNA-induced RLR signaling. α-Tubulin served as a loading control. The corresponding quantification is shown in Fig. S4A. **C** Western blot analysis of 1KO KCs transfected with the indicated siPools prior to pIC stimulation. Left: representative immunoblots of RLR pathway components and phosphorylated IRF3 (p-IRF3) as a marker of pathway activation. α-Tubulin served as a loading control. Right: Densitometric quantification of p-IRF3 levels normalized to those of α-tubulin. The data represent the fold changes ± SDs relative to control siPool-transfected, pIC-stimulated 1KO KCs; *n* = 4. The quantification of the knockdown efficiency is shown in Fig. S4B. **D** Luciferase reporter assays measuring NFκB and IRF activity in siPool-transfected and pIC-stimulated 1KO KCs. RE-Luc activity was normalized to Renilla luciferase luminescence. The data are presented as the mean fold changes ± SDs relative to control siPool-transfected, pIC-stimulated 1KO KCs; *n* = 4. **E** 1KO KCs transfected with the indicated siPools were stimulated with pIC. After 24 h, the levels of secreted IFN-β1, IL-6 and TNFα were quantified by ELISA. The data represent the fold changes ± SDs relative to control siPool-transfected, pIC-stimulated 1KO KCs; *n* = 4. Statistical significance was determined by one-way ANOVA with Tukey’s multiple comparison test. n.s. = not significant; * *p* < 0.05; *** *p* < 0.001

Since pIC-stimulated PKP1-KO KCs exhibit increased cytokine secretion in an IRF3- and NFκB-dependent manner, we investigated how PKP1 acts to control the activity of IRF3 and NFκB. The RLRs MDA5, RIG-I and LGP2 have been described as dsRNA sensors whose activation induces the formation of the MAVS complex and the IRF3- and NFκB-dependent production of type I IFNs such as IFN-β1 and other proinflammatory cytokines, including IL-6 and TNFα [Fig. 5A, [49]]. We first assessed the protein levels of MDA5, RIG-I, and LGP2 in WT, PKP1-KO and PKP1-rescued KCs 6 h after pIC transfection (Figs. 5B and S4A). RIG-I and LGP2 were readily detected in all unstimulated and pIC-stimulated KC cell lines, but the differences between PKP1-expressing and PKP1-KO KCs were not significant. In contrast, the expression of MDA5 largely depended on pIC transfection and was slightly lower in PKP1-KO KCs than in WT KCs and in PKP1-rescued KCs. Furthermore, pIC transfection reduced the MAVS levels in all the cell lines, as previously described [50, 51], but no major differences were detected between the KC cell lines. These data suggest that PKP1 does not attenuate inflammatory signaling by modulating the baseline expression of RLRs or their adapter MAVS.

To determine whether PKP1 limits innate immunity by modulating RLR signaling, we utilized siPools to deplete RIG-I, MDA5, LGP2 and MAVS in PKP1-KO KCs prior to pIC stimulation. pIRF3 levels were determined to be indicators of the activation of dsRNA-dependent inflammatory responses (Figs. 5C and S4B). Despite a very efficient knockdown, RIG-I depletion had no effect on pIRF3 levels. Similarly, depletion of LGP2, a known facilitator of MDA5-driven responses [34, 35], only slightly reduced IRF3 phosphorylation. In contrast, the knockdown of MDA5 or MAVS expression significantly decreased pIC-dependent IRF3 phosphorylation in PKP1-KO KCs. These findings demonstrate that the hyperactivation of innate immunity upon PKP1 loss is selectively channeled through the MDA5/MAVS signaling cascade, which is consistent with the known preference of MDA5 for long dsRNA ligands [52].

To validate the PKP1-dependent role of MDA5, we determined the NF-κB- and IRF-RE-dependent luciferase activity in pIC-transfected KC cells with knockdown of MDA5, RIG-I, LGP2 and MAVS (Fig. 5D). The knockdown of both MDA5 and MAVS dramatically reduced the activity of both reporters, whereas the knockdown of RIG-I had no effect. LGP2 depletion slightly but significantly reduced NFκB- and IRF-RE-dependent luciferase activity, in agreement with its role in facilitating MDA5 signaling. The decreased activation of NFκB and IRF upon depletion of LGP2, MDA5 and MAVS culminated in the significantly reduced secretion of IFN-β1, IL-6 and TNFα in response to pIC transfection, whereas RIG-I had no effect on cytokine secretion (Fig. 5E). Consistent with the results of WB and luciferase reporter gene assays, LGP2 depletion had a weaker effect on the levels of IFN-β1, IL-6 and TNFα than did MDA5 or MAVS knockdown. We conclude that PKP1 interferes with the MDA5/MAVS-dependent activation of p65 and IRF3 to limit the secretion of proinflammatory cytokines in response to dsRNA. Thus, PKP1 acts either upstream of MDA5 or by directly regulating MDA5.

### PKP1-dependent transcriptional control of proinflammatory cytokines depends on DExD/H-box helicases

**Fig. 6:**
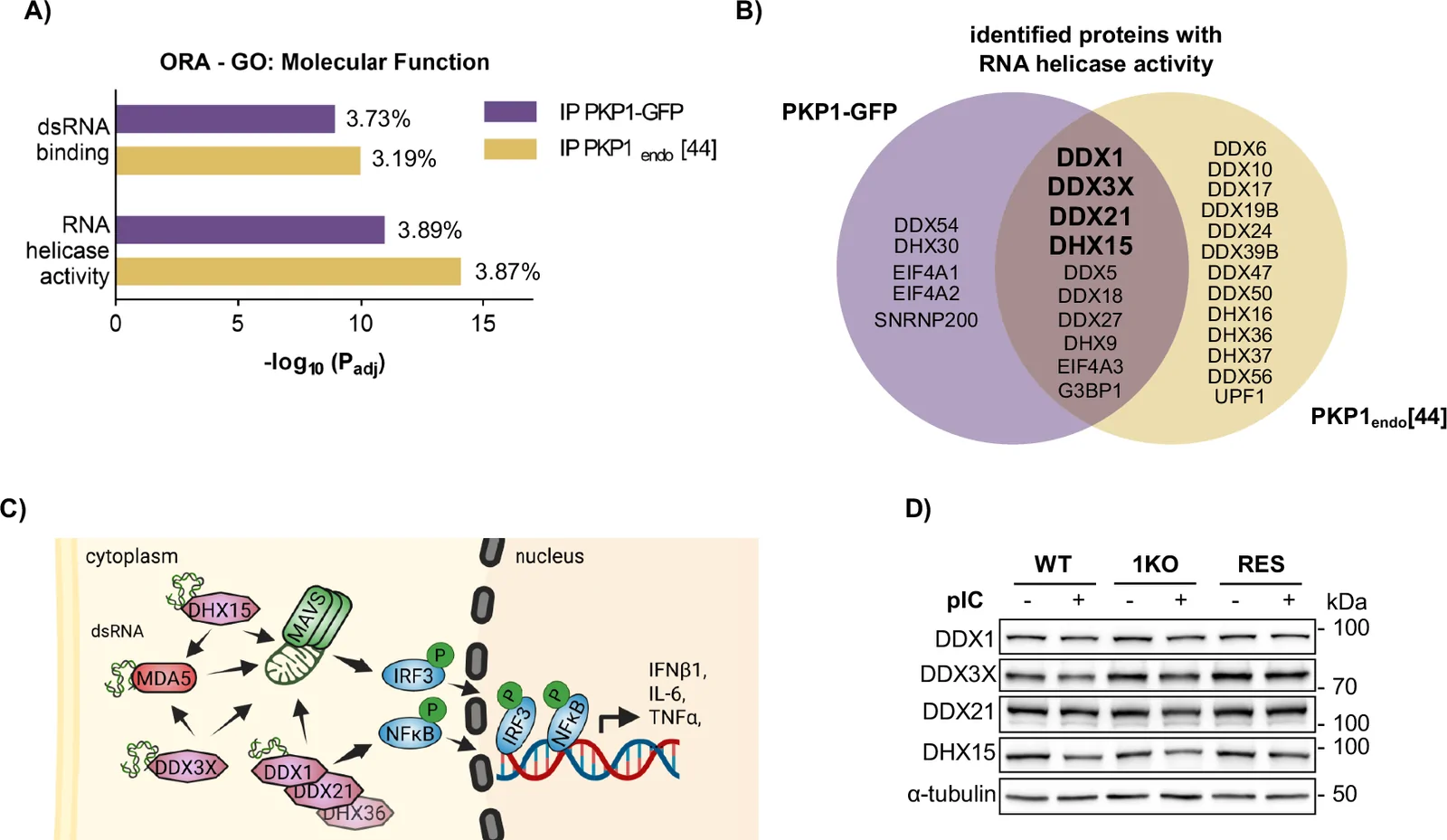
PKP1 is associated with DExD/H-box helicases. **A** *Overrepresentation* analysis (ORA) of putative PKP1 interaction partners. Analysis was performed using g: GOSt based on proteins identified by mass spectrometry after PKP1-GFP-Trap in this study and by endogenous PKP1 immunoprecipitation from the lung squamous cell carcinoma cell line EPCL-272H reported by Martin-Padron et al. *[44]. **B** Overview of the identified proteins annotated with RNA helicase activity. The Venn diagram compares proteins identified by the PKP1-GFP-Trap in this study with proteins identified in the published endogenous PKP1 immunoprecipitation dataset from EPCL-272H cells *[44]. **C** Schematic representation of DExD/H-box helicases supporting the MDA5/MAVS-dependent activation of NFkB and IRF3 mediated by dsRNA. Created in BioRender. **D** Representative immunoblots showing DExD/H-box helicase expression in mock- and pIC-transfected WT, PKP1-KO (1KO) and PKP1-rescue (RES) KCs. α-Tubulin served as a loading control. The corresponding quantification is shown in Fig. S5

To elucidate how PKP1 controls MDA5 activity, we identified PKP1-associated proteins by mass spectrometry (MS) analysis of proteins that were copurified with PKP1-WT-GFP. Interestingly, overrepresentation analysis (ORA) of our MS data, alongside a publicly available dataset of endogenous PKP1 interactors [44], revealed a robust association between PKP1 and dsRNA-binding proteins and RNA helicases (Fig. 6A). Notably, LGP2, RIG-1, MDA5 and MAVS were not coprecipitated, suggesting that PKP1 indirectly regulates MDA5/MAVS activity. Of particular interest in this respect was the robust association of PKP1 with the DExD/H-box helicases DDX1, DDX3X, DDX21 and DHX15, which trigger or enhance dsRNA-driven responses [Fig. 6B, C, [53, 54]].

**Fig. 7:**
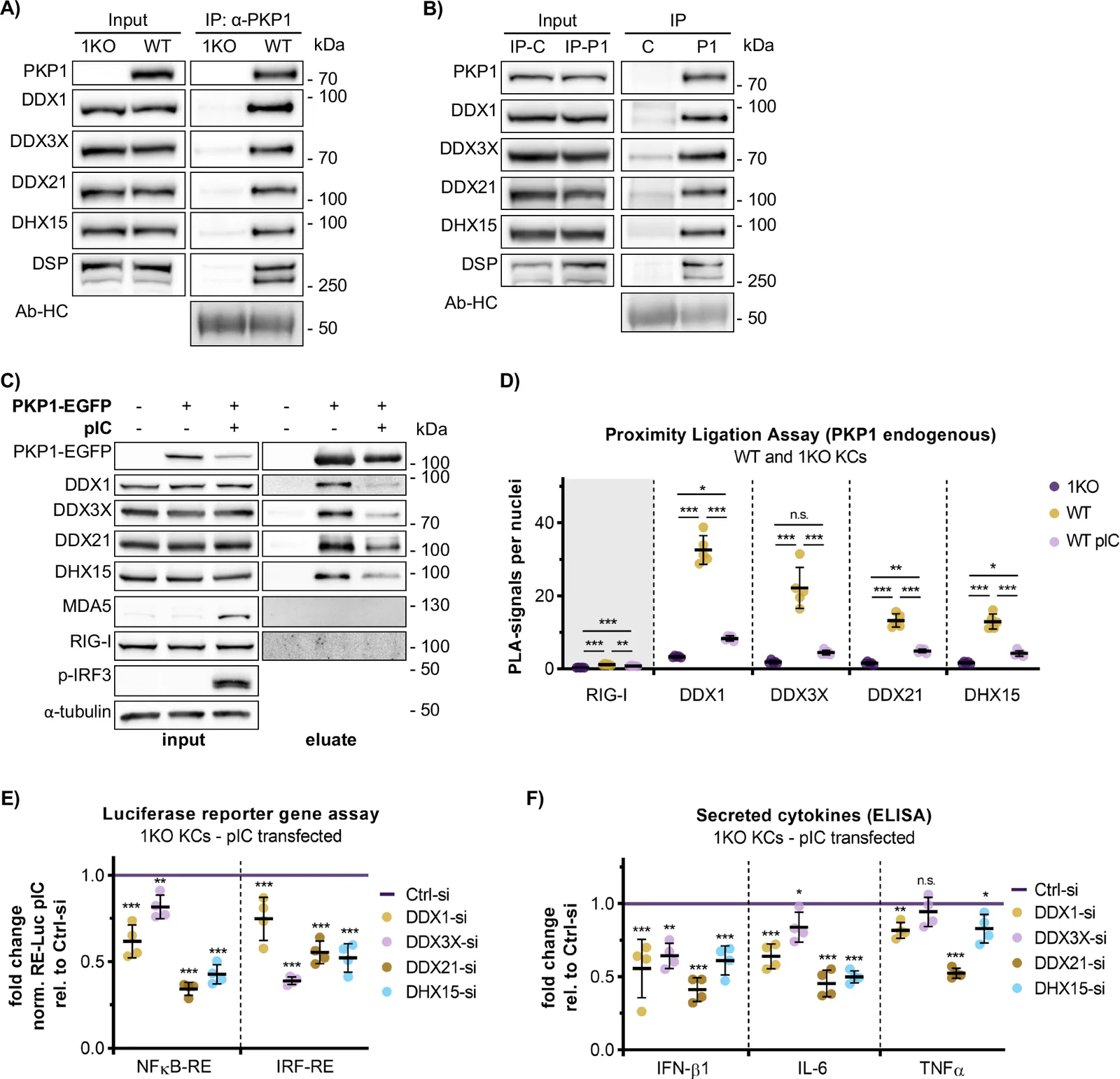
PKP1 restricts DExD/H-box helicase-dependent NFκB and IRF transcription factor activity and cytokine secretion. **A** Interaction of endogenous PKP1 with DExD/H-box helicases in murine KCs. Endogenous PKP1 was immunoprecipitated from WT KCs, while PKP1-KO (1KO) KCs served as a negative control. Representative immunoblots from four independent experiments are shown. **B** Interaction of endogenous PKP1 with DExD/H-box helicases in human HaCaT cells. Endogenous PKP1 was immunoprecipitated using guinea pig PKP1 antiserum; guinea pig preimmune serum served as a control. Representative immunoblots from four independent experiments are shown. **C** GFP-Trap experiments using mock- and pIC-transfected PKP1-rescue KCs. EGFP-expressing PKP1-KO KCs served as controls. Representative immunoblots of protein extracts (input) and precipitated fractions (eluate) are shown. The corresponding quantification of precipitated helicases is shown in Fig. S6A. **D** DuoLink proximity ligation assay (PLA) detecting endogenous PKP1 in proximity to the indicated DExD/H-box helicases in mock- and pIC-transfected WT KCs. 1KO KCs served as a specificity control. PLA signals were quantified and normalized to the number of nuclei. Five images per condition from two independent experiments were used for quantification. Representative images are shown in Fig. S6B. **E** Luciferase reporter assays measuring NFκB and IRF activity in 1KO KCs transfected with the indicated siPools and stimulated with pIC. RE-Luc activity was normalized to Renilla luciferase luminescence. The data are presented as the mean fold changes ± SDs relative to control siPool-transfected, pIC-stimulated 1KO KCs; *n* = 4. **F** 1KO KCs were transfected with the indicated siPools and stimulated with pIC. After 24 h, the levels of secreted IFN-β1, IL-6 and TNFα were quantified by ELISA. The data represent the fold changes ± SDs relative to control siPool-transfected, pIC-stimulated 1KO KCs; *n* = 4. Statistical significance was determined by one-way ANOVA with Tukey’s multiple comparison test. n.s. = not significant; * *p* < 0.05; ** *p* < 0.01; *** *p* < 0.001

All four helicases were readily detected in all investigated KC cells and showed only subtle changes in abundance upon pIC transfection, PKP1 depletion or re-expression (Figs. 6D and S5). To analyze in detail how PKP1 might interfere with inflammatory responses through these helicases, we performed immunoprecipitation (IP) experiments in both murine KCs (using PKP1-KO cells as a negative control; Fig. 7A) and human HaCaT cells (Fig. 7B). DDX1, DDX3X, DDX21 and DHX15 were copurified with PKP1 from both cell types, confirming the MS findings. We then utilized PKP1-GFP rescue KCs to assess the dynamics of these interactions following pIC transfection (Fig. 7C). Neither MDA-5 nor RIG-I, but again, DDX1, DDX3X, DDX21 and DHX15 were copurified with PKP1-GFP from unstimulated or pIC-transfected KCs. Interestingly, the amount of PKP1-GFP was considerably reduced in the input samples at 6 h after pIC transfection. Intriguingly, the pIC-induced reduction in PKP1-GFP abundance was accompanied by substantially reduced PKP1 association with the respective DExD/H-box helicases (Figs. 7C and S6A). These observations suggest the following two nonmutually exclusive mechanisms: [1] PKP1 binding to DExD/H-box helicases, impairing their ability to process dsRNA signals, and [2] dsRNA recognition triggering the rapid degradation of PKP1, thereby releasing these helicases to facilitate signaling.

To validate these data in a cellular setting, we used the DuoLink proximity ligation assay (PLA) approach to monitor endogenous PKP1-DExD/H-box helicase complexes in unstimulated and pIC-stimulated WT KCs. The PLA signals for the PKP1-helicase complexes were normalized to the nuclear count, with the PKP1-KO cells providing a baseline for a nonspecific background. We observed at least a 10-fold increase in PLA signals for PKP1-helicase associations relative to those of the KO controls, whereas no interaction was detected for RIG-I (Figs. 7D and S6B). Importantly, pIC stimulation significantly reduced the association of endogenous PKP1 with all four helicases.

Finally, we assessed the functional necessity of these helicases. The depletion of these genes in PKP1-KO KCs significantly diminished IRF3 phosphorylation (Fig. S5C), although the effect was less pronounced than that observed in cells in which MDA5 or MAVS was knocked down (Fig. 5C). Consistently, the activity of NFκB and IRF luciferase reporters, as well as cytokine secretion, was reduced by helicase depletion in pIC-transfected PKP1-KO cells (Fig. 7E, F). Collectively, these data demonstrate that PKP1 restrains DExD/H-box helicase functions by recruiting them to repressive complexes. The stimulus-dependent release of these helicases appears to be a prerequisite for the activity of the MDA5/MAVS signaling axis and subsequent cytokine production.

### Binding of PKP1 to DExD/H-box helicases is regulated by PKP1 stability

**Fig. 8:**
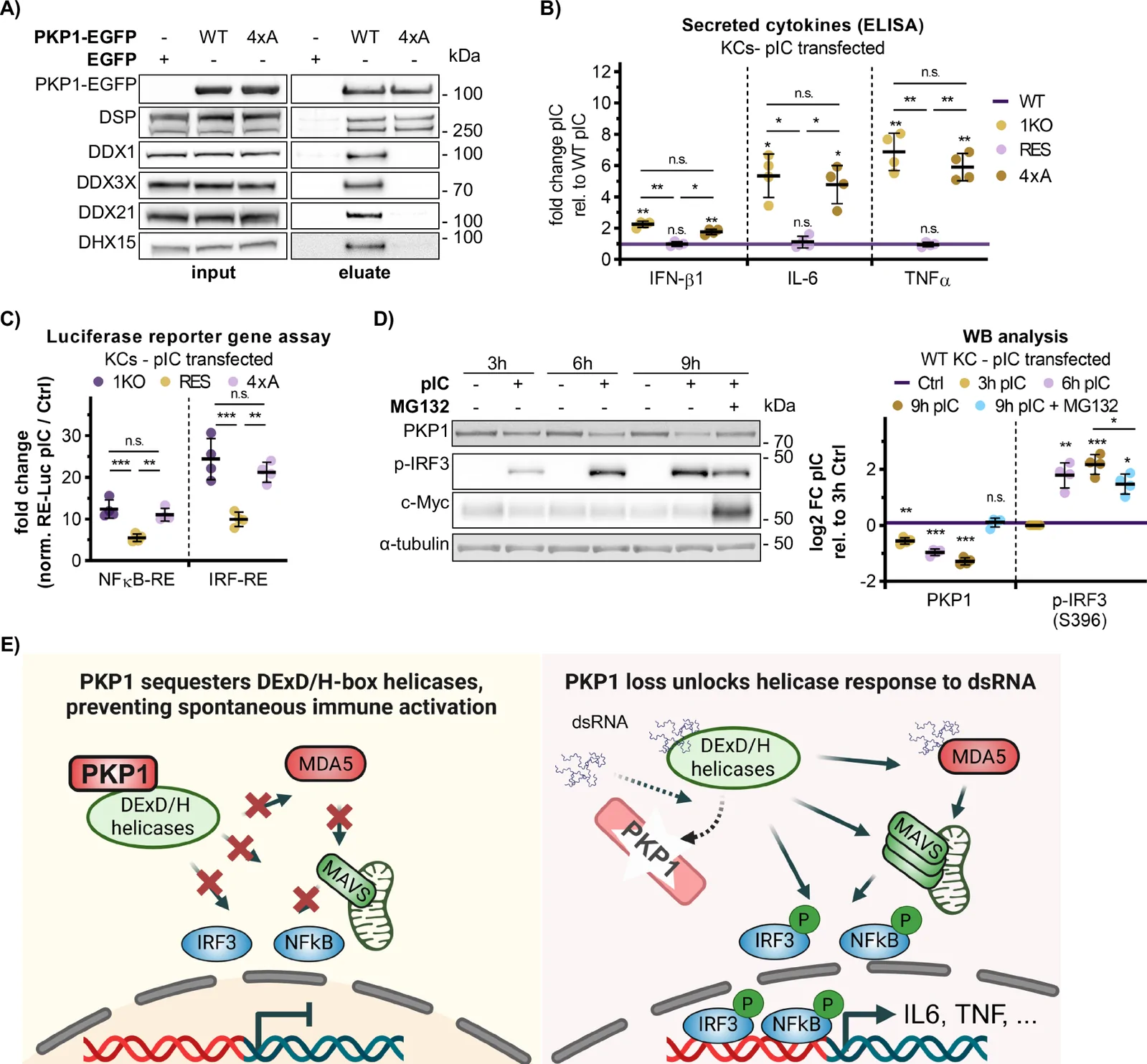
PKP1-mediated suppression of MDA5/MAVS signaling through DExD/H-box helicases is regulated by PKP1 modification and stability. **A** GFP-Trap experiment using PKP1-KO KCs expressing EGFP, PKP1-WT-EGFP or PKP1-4xA-EGFP. Representative immunoblots of protein extracts (input) and precipitated fractions (eluate) are shown. Desmoplakin (DSP) served as a positive control for retained desmosomal binding of the PKP1-4xA mutant. **B** ELISA-based quantification of IFN-β1, IL-6 and TNFα secretion in WT, PKP1-KO (1KO), PKP1-rescue (RES) and PKP1-4xA-EGFP (4xA) KCs 24 h after pIC transfection. The data represent the fold changes ± SDs relative to pIC-stimulated WT KCs; *n* = 4. **C** Luciferase reporter assays measuring NFκB and IRF transcription factor activity in pIC-stimulated 1KO, RES and 4xA KCs. RE-Luc activity was normalized to Renilla luciferase luminescence. The data represent the mean fold changes ± SDs of normalized RE-Luc activity; *n* = 4. **D** Western blot analysis of PKP1 protein stability and IRF3 activation in mock- and pIC-transfected WT KCs with or without proteasome inhibition by MG132. Left: representative immunoblots of PKP1, p-IRF3 and c-Myc. α-Tubulin served as a loading control. Right: Densitometric quantification of PKP1 and p-IRF3 levels normalized to that of α-tubulin. Data show log2-fold changes ± SDs relative to those of mock-transfected WT KCs at 3 h; *n* = 4. **E** Proposed model of PKP1-mediated suppression of RLR signaling by sequestration of DExD/H-box helicases. Under steady-state conditions, PKP1 binds DExD/H-box helicases and limits spontaneous or excessive dsRNA-induced inflammatory signaling. Upon dsRNA stimulation, the proteasomal degradation of PKP1 releases these helicases, enabling the MDA5/MAVS-dependent activation of IRF3 and NFκB. PKP1 deficiency leads to enhanced MDA5-dependent signaling and increased cytokine secretion. Created with BioRender. Statistical significance was determined by one-way ANOVA with Tukey’s multiple comparison test. n.s. = not significant; * *p* < 0.05; ** *p* < 0.01; *** *p* < 0.001

To investigate whether posttranslational modifications regulate the binding of PKP1 to DExD/H-box helicases, we utilized a nonphosphorylatable PKP1 mutant (Fig. S7A) [43] that was re-expressed in PKP1-KO KCs (PKP1-4xA). In contrast to PKP1-WT, which is localized in desmosomes, the nucleus and the cytoplasm, the PKP1-4xA mutant is localized almost exclusively to desmosomes (Fig. S7B) [43]. Strikingly, in GFP-Trap studies, PKP1-4xA failed to associate with any of the four helicases (Fig. 8A), which is in agreement with its failure to localize to the cytosol. In contrast, the association with desmosomal desmoplakin (DSP) was maintained as previously described [43]. Functionally, PKP1-4xA also failed to suppress NFκB and IRF activation or the subsequent production of proinflammatory cytokines upon pIC transfection (Fig. 8B, C). Consequently, compared with PKP1-KO cells, PKP1-4xA-expressing cells exhibited a proinflammatory profile, strongly supporting the hypothesis that PKP1 dampens the production of inflammatory cytokines by sequestering helicases in a desmosome-independent, cytosolic manner.

We further hypothesized that PKP1 protein decay in response to dsRNA cues might enable the activation of an inflammatory response. The levels of endogenous PKP1 were diminished by pIC transfection in a time-dependent manner in both human HaCaT cells and murine WT KCs (Figs. 8D and S8). p-IRF3 levels were inversely correlated with PKP1 levels and increased after pIC stimulation (Fig. 8D). Strikingly, the protein abundance of PKP1 and c-MYC (internal control) was substantially elevated by proteasome inhibition via MG132 in pIC-transfected KCs. The stabilization of PKP1 by MG132 treatment correlated with decreased p-IRF levels, confirming the inverse correlation (Fig. 8D). These data strongly suggest that the activation of MDA5/MAVS signaling requires the release of DExD/H-box helicases from PKP1 complexes, a process facilitated by the proteasomal degradation of PKP1.

In summary, we show that PKP1 suppresses dsRNA-induced inflammatory responses by sequestering DExD/H-box helicases, thereby limiting MDA5/MAVS signaling. As a consequence, IRF3 and NFκB activation and the production of type I IFNs and proinflammatory cytokines are suppressed. To allow for a physiological response, PKP1 is degraded in response to inflammatory stimuli, releasing the inhibition of the MDA5/MAVS axis. However, the permanent loss of PKP1 led to an amplified and uncontrolled inflammatory response (Fig. 8E).

## Discussion

The physical and immunological barrier components of the skin are highly interdependent. A compromised physical barrier permits pathogen entry and facilitates the release of DAMPs from stressed or injured cells, thereby triggering the inflammatory responses required for microbial clearance and tissue repair. Conversely, dysregulated inflammation can compromise barrier integrity and impair wound healing. Thus, epidermal homeostasis requires a precise balance between structural cohesion and innate immune responsiveness [24, 27].

Emerging evidence supports the concept that desmosomal proteins regulate inflammatory responses beyond their structural functions [reviewed previously [55]]. Loss-of-function mutations in DSG1 cause SAM syndrome, which is characterized by epidermal barrier defects, allergic inflammation and elevated keratinocyte-derived cytokines, whereas DSG1-deficient skin displays IL-17-driven inflammatory signatures [56, 57]. Mutations in DSP can also cause SAM syndrome and are linked to inflammatory cardiomyopathy with myocardial necrosis and lymphocytic infiltration [58–61]. Similarly, plakophilins have been implicated in immune regulation: PKP2 deficiency in cardiomyocytes induces inflammatory transcriptional programs, PKP3 loss enhances local and systemic inflammatory responses, and PKP1 deficiency in prostate tumors is correlated with immune cell recruitment and increased cytokine mRNA stability[23, 62–67]. These findings indicate that desmosomal proteins are not passive structural components but actively participate in balancing tissue cohesion and inflammatory signaling. However, the molecular mechanisms connecting desmosomal proteins to innate immune pathways have remained incompletely understood.

Our study identified PKP1 as a negative regulator of dsRNA-induced innate immune signaling in KCs. PKP1 combines two protective functions in stratified epithelia. First, through its established role in desmosomal adhesion and tight junction formation [15], it strengthens the physical barrier and limits the entry of potentially harmful environmental agents. Second, through desmosome-independent cytoplasmic function, PKP1 restrains dsRNA-induced inflammatory signaling. Specifically, PKP1 decoys DExD/H-box RNA helicases and limits their availability for MDA5/MAVS-dependent activation of IRF3 and NFκB. This prevents exaggerated cytokine production in response to exogenous or endogenous dsRNAs and may thereby protect epithelial tissues from chronic or self-amplifying inflammatory states. Consistently, PKP1 loss increased basal and stimulus-induced cytokine production in murine and human KCs and induced inflammatory transcriptional programs that were rescued by PKP1 re-expression (Figs. 1, 2 and S1, S2).

Mechanistically, PKP1 acts upstream of IRF3 and NFκB activation. The strong NFκB reporter activity and p65 nuclear translocation in PKP1-KO cells, together with the relatively modest change in p65 S536 phosphorylation, likely reflect the multilayered regulation of NFκB. p65 activation is controlled by IκB degradation, nuclear import, DNA binding, coactivator recruitment and multiple posttranslational modifications, not by S536 phosphorylation alone [68]. Thus, the combined reporter, IKKα/β phosphorylation, p65 translocation and cytokine data support enhanced NFκB activation in PKP1-KO cells (Figs. 3, 4 and S3).

Our data further demonstrated that the exaggerated inflammatory response caused by PKP1 loss is selectively channeled through MDA5/MAVS signaling (Fig. 5 and S4). The knockdown of MDA5 or MAVS strongly reduced IRF3 phosphorylation, NFκB and IRF reporter activity, and cytokine secretion in pIC-stimulated PKP1-KO cells. RIG-I depletion had no detectable effect, and LGP2 depletion had only a partial effect, which is consistent with its role as a facilitator of MDA5 signaling [34, 35, 52]. Since PKP1 did not copurify with MDA5 or RIG-I, PKP1 most likely controls MDA5/MAVS signaling indirectly rather than by binding the core RLR pathway components themselves.

The PKP1 interactome provides a mechanistic explanation for this indirect regulation. PKP1 is associated with several DExD/H-box RNA helicases, including DDX1, DDX3X, DDX21 and DHX15, all of which have been implicated in dsRNA-dependent innate immune signaling [36–39, 53, 54]. Depletion of these helicases in PKP1-KO keratinocytes reduced IRF3 phosphorylation, NF-κB and IRF reporter activity, and cytokine secretion, although these effects were weaker than those resulting from MDA5 or MAVS depletion. Thus, these helicases are not simply redundant with MDA5 in KCs. Rather, they appear to function as nonredundant accessory factors that support efficient MDA5/MAVS pathway activation. In murine KCs, however, DDX helicases cannot compensate for the loss of MDA5/MAVS, indicating that their function is cell type- and pathway context-dependent (Figs. 6, 7 and S5, S6). Whether PKP1 only sequesters DExD/H-box helicases or alters their biochemical activity remains unresolved. The current data strongly support sequestration, but PKP1 has previously been shown to stimulate eIF4A1 activity [69], suggesting that it may also influence ATPase activity and the RNA-binding or scaffold functions of other helicases. Since several innate immune functions of DExD/H-box helicases are scaffold-like and not always dependent on classical helicase activity [38, 54, 70], PKP1 may primarily limit its access to RNA substrates or MAVS-proximal signaling complexes.

Strikingly, PKP1-helicase interactions were dynamically disrupted upon pIC stimulation, as shown by the results of the GFP-trap and PLA analyses, and this coincided with the proteasome-dependent loss of PKP1 (Figs. 7C, D, 8D). Conversely, the nonphosphorylatable PKP1-4A mutant, which is largely restricted to desmosomes and retains DSP binding, failed to associate with DDX1, DDX3X, DDX21 or DHX15 and failed to suppress NF-κB, IRF and cytokine responses (Fig. 8A–C and S7). These findings indicate that the immunoregulatory function of PKP1 depends on its phosphorylated cytoplasmic pool rather than on its adhesive desmosomal function. PKP1 deactivation during inflammatory signaling likely occurs through linked mechanisms: loss of the cytoplasmic helicase-binding pool and proteasomal degradation of PKP1.

We have previously shown that AKT2-mediated phosphorylation stabilizes cytoplasmic PKP1 [43]. Thus, under steady-state conditions, phosphorylated PKP1 may promote or stabilize PKP1-helicase complexes, thereby maintaining an anti-inflammatory state. Upon dsRNA stimulation, the loss of stabilizing phosphorylation, either through active dephosphorylation or reduced kinase activity, may destabilize cytosolic PKP1, facilitate E3 ligase recruitment, and promote its proteasomal degradation. This releases sequestered DExD/H-box helicases and allows the activation of both IRF3 signaling and NFκB signaling. In this context, activation of PP1α upon dsRNA stimulation promotes MDA5 and RIG-I dephosphorylation and activation, enabling a functional innate immune response [71]. Whether PP1α, or another phosphatase, also contributes to PKP1 dephosphorylation remains to be determined. Identifying the relevant phosphatases, kinases, and E3 ligases will be important in the future.

This model also provides a framework for understanding PKP1 in human disease. Loss-of-function mutations in PKP1 cause ectodermal dysplasia–skin fragility syndrome, which is characterized not only by mechanical fragility but also by recurrent infections and inflammatory manifestations [13, 14]. These clinical features have largely been attributed to barrier failure. Our findings suggest the following mechanism: PKP1-deficient keratinocytes may be intrinsically prone to excessive dsRNA-dependent inflammatory activation. This is physiologically relevant because pIC can activate MDA5/MAVS-dependent signaling in human epidermal immune contexts, including keratinocytes and Langerhans cells [72]. Therefore, PKP1 loss may compromise epidermal homeostasis through both physical barrier weakening and immune dysregulation.

Our findings not only reveal a role for PKP1 in limiting excessive inflammation in the skin but also point to a broader role in squamous cell carcinogenesis. In particular, PKP1 is frequently overexpressed in lung squamous cell carcinoma (LUSC), where it has been linked to enhanced MYC translation, increased proliferation, and tumor growth [44–46]. Our transcriptomic data closely mirror this oncogenic role: PKP1-low tumors were enriched for inflammatory pathways overlapping with those induced in pIC-stimulated PKP1-KO KCs, whereas PKP1-high tumors were associated with c-MYC and mTOR signaling. Although these correlative findings do not provide direct functional evidence of immune evasion, they support a dual oncogenic model in which PKP1-high tumors combine MYC-linked growth programs with a more immune-restrained transcriptional state.

In summary, our findings position PKP1 as a central hub that coordinates epithelial adhesion, RNA-helicase availability and innate immune responsiveness. By sequestering DExD/H-box helicases, PKP1 limits the MDA5/MAVS-dependent activation of IRF3 and NFκB. Upon dsRNA stimulation, the proteasomal degradation of PKP1 releases this brake and permits a physiological inflammatory response. Permanent PKP1 loss, however, uncouples this regulatory mechanism and produces an amplified inflammatory state. This mechanism may be relevant for inflammatory skin disease, PKP1-associated skin fragility syndromes and squamous tumor biology (Fig. 8E).

## Materials and methods

### Animals

Newborn PKP1^–/–^ (PKP1-KO) mice and their PKP1^+/+^ (WT) littermates were used in this study. The generation of PKP1^–/–^ mice has been described previously [15]. PKP1^+/–^ mice backcrossed to C57BL/6 J (Charles River Laboratories, Germany) mice were inbred to obtain PKP1^–/–^ and PKP1^+/+^ animals. All mouse procedures were approved by the local authorities (Landesverwaltungsamt Saxony-Anhalt permit number: 2-1482) and performed in accordance with institutional policies.

### Cell lines, cell culture and treatments

Immortalized KCs from WT and PKP1-KO mice, as well as PKP1-KO KCs expressing PKP1-WT-GFP or GFP, have been described previously [15, 16]. PKP1-KO KCs expressing a phosphorylation-deficient PKP1 mutant termed “PKP1-4xA” were generated by lentiviral transduction as previously described [16, 43].

KCs were grown on 15 µg/ml collagen I (Corning, Glendale, Arizona, USA) in low-calcium medium (LCM; DMEM/Ham’s F12 medium containing 50 μM CaCl_2_, 10% (v/v) Ca^2+^-free FCS, 1 mM sodium pyruvate, 1 mM glutamate, 0.18 mM adenine, 0.5 μg/ml hydrocortisone, 5 μg/ml insulin, 10 ng/ml EGF, 100 pM cholera toxin, and 1 mg/ml glucose) at 32 °C in 5% CO_2_ and 90% humidity. To induce differentiation, the cells were switched to high-calcium medium (HCM; LCM supplemented with 1.2 mM CaCl_2_).

Human HaCaT keratinocytes were cultured in DMEM supplemented with 10% FCS and antibiotics at 37 °C in 5% CO_2_. For PKP1 knockdown, HaCaT cells were transfected with control or PKP1-directed siRNAs using Lipofectamine RNAiMAX according to the manufacturer’s instructions. The knockdown efficiency was verified by Western blotting.

Unless stated otherwise, the experiments were performed according to the standardized scheme shown in Fig. S1B. On day 1, KCs were seeded at a density of 0.5 × 10^5^ cells/cm^2^ in LCM, directly transfected with siRNA pools if needed and incubated at 32 °C for 24 h. On day 2, the cells were switched to HCMs and, where needed, transfected with luciferase reporter constructs. On day 3, the HCMs were exchanged, and the cells were either left untreated, mock-transfected, or transfected with pIC or exposed to UVB irradiation. Cells were harvested after 6 h for qRT‒PCR, Western blotting, immunofluorescence or GFP‒Trap experiments and after 24 h for ELISA or luciferase reporter assays. For proteasome inhibition, WT KCs were stimulated with pIC for 3 h before 10 µM MG132 (Sigma‒Aldrich, Germany) or the DMSO vehicle control was added for the remaining 6 h of incubation.

### Protein lysate preparation from the dorsal skin of newborn mice

For analysis of proinflammatory cytokine expression, pieces of back skin from newborn WT and PKP1-KO mice were homogenized with T25 ULTRA-TURRAX (IKA, Staufen, Germany) in cell lysis buffer (10 mM Tris-HCL; pH 7.5, 20 mM Na_4_P_2_O_7_, 100 mM NaCl, 1% Triton X-100, 0.5% deoxycholate, 0.1% SDS, 10% glycerol, and 1 mM EDTA supplemented with 1 μg/ml aprotinin, 1 μg/ml leupeptin, 1 mM Pefabloc, 5 mM NaF, and 1 mM Na_3_VO_4_). Skin extracts were centrifuged for 15 min at 13,000 *g* and 4 °C, and the protein concentration of the supernatants was determined using a Pierce^TM^ Micro BCA Protein-Assay Kit (Thermo Scientific) according to the manufacturer’s instructions.

### Transfection of siRNA pools

The siRNA pools (defined pools of 30 selected siRNAs; Table S1) were obtained from siTools Biotech GmbH (Martinsried, Germany) and transfected using Lipofectamine® RNAiMax (Thermo Fisher Scientific) according to the manufacturer’s instructions. KCs were directly transfected in suspension with 2 pmol/ml of the respective siRNA pools and kept under LCM for 24 h.

### Transfection of pIC

To stimulate murine keratinocytes with dsRNA, high-molecular-weight polyinosinic-polycytidylic acid (pIC; 0.1 µg/ml; InvivoGen, #tlrl-pic) was transfected using Lipofectamine RNAiMAX according to the manufacturer’s instructions. For HaCaT cells, 20 ng/ml high-molecular-weight pIC was transfected under otherwise identical conditions. Mock transfection was performed as a control. Cells were harvested 6 h after pIC transfection for qRT‒PCR, Western blotting, immunofluorescence or GFP-Trap experiments and 24 h after transfection for ELISA or luciferase reporter assays.

### Flow cytometric analysis of Cy3-pIC transfection efficiency

To determine the pIC transfection efficiency, WT, PKP1-KO and PKP1-rescue KCs were transfected with Cy3-labeled pIC (InvivoGen, tlrl-piccy3) under the same conditions used for the stimulation experiments. At 6 h after transfection, the cells were washed with PBS, detached using trypsin/EDTA, collected in medium, and pelleted by centrifugation. The cells were resuspended in PBS supplemented with 1% FCS and analyzed by flow cytometry (BD FACS Melody). Mock-transfected cells were used to define the Cy3-negative population and to set the threshold for Cy3-positive cells. The data were analyzed as the median fluorescence intensity.

### UVB irradiation

KCs kept for 24 h in HCM were switched to PBS prior to UVB irradiation. KCs were UVB irradiated at 2 J/cm^2^ using a Waldmann TP-4 UV therapy system equipped with four 36 W narrowband UVB (311 nm) lamps. The KCs were subsequently rinsed twice in PBS before they were incubated in HCMs for 24 h, after which cytokine secretion was analyzed.

### Luciferase reporter gene assays

Nontransfected or siRNA pool-transfected KCs grown in LCM for 24 h were switched to HCM and cotransfected with 0.9 µg/ml of a firefly luciferase reporter vector (Promega) carrying NFκB (pGL4.32 [luc2P/NFκB-RE]) or IRF (pGL4.45 [luc2P/ISRE]) response elements and 0.1 µg/ml of the pRL-SV40 vector (Promega) encoding Renilla luciferase for normalization using Lipofectamine 2000 (Thermo Fisher Scientific) according to the manufacturer’s instructions. Luciferase activity was measured 24 h after MOCK or pIC transfection using a Dual-Luciferase Reporter Assay System (Promega) and an Infinite® M PLEX plate reader (Tecan, Switzerland) in accordance with the manufacturer’s protocol. The response element-dependent firefly luciferase activities were normalized to the corresponding Renilla luciferase activities, and fold changes relative to the MOCK-transfected sample were calculated.

### ELISA

To analyze the secretion of proinflammatory cytokines, untreated keratinocytes or keratinocytes stimulated with UVB or pIC were maintained for 24 h in fresh HCMs. The supernatants were subsequently collected and centrifuged for 3 min at 1500 *g* and 4 °C, after which 100 µl of the cleared supernatants was used to quantify the amounts of CXCL1, IL-1α, IL-6, IFN-β1, and TNFα by using the ELISA kits listed in Table S2.

### Protein lysate preparation, SDS‒PAGE, and Western blotting

For protein expression analysis, KCs were lysed in SDS lysis buffer [20 mM Tris-HCl; pH 7.5, 1% SDS; supplemented with 1x Halt^TM^ protease and phosphatase inhibitor cocktail (Thermo Fisher Scientific) and 125 U/ml Benzonase (Santa Cruz Biotechnology)] and centrifuged for 15 min at 13,000 *g*. The protein concentration was determined using the Pierce^TM^ Micro BCA Protein-Assay-Kit (Thermo Scientific) according to the manufacturer’s instructions. Equal amounts of protein were separated by SDS‒PAGE, and the proteins were transferred to Amersham^TM^ Protan^TM^ nitrocellulose blotting membranes (0.2 µm pore size, Th. Geyer, Germany) using Mini Trans-Blot cells (Bio-Rad Laboratories, Hercules, California, USA). After transfer, the membranes were cut, blocked with 3% skim milk/TBST or 3% bovine serum albumin (BSA)/TBST, and subsequently probed overnight with the appropriate primary antibodies (Table S3). The membranes were subsequently washed three times with TBST and incubated for 1 h with the appropriate horseradish peroxidase-conjugated secondary antibodies (Table S4). The membranes were covered with Western Blot Ultra-Sensitive HRP Substrate (Takara), and chemiluminescence was detected using a Fusion-SL 3500WL imaging system (Peqlab, Erlangen, Germany). If staining with additional antibodies was needed, the membranes were washed three times in TBST for 10 min each, incubated in stripping buffer (0.2 M glycine; pH 2.5, 0.05% tween-20) for 1 h, washed again in TBST (3×10 min), blocked, and treated with the antibodies described above. β-actin staining served as the loading control. Fiji software was used for image processing [73]. For image processing, only linear adjustments of brightness and contrast were applied to improve clarity. No further modifications or alterations of the image data were performed.

### GFP-Trap® immunoprecipitation

For the GFP-Trap® experiments, 24×10^5^ (mass spectrometry, MS) or 8×10^5^ (WB analysis) PKP1-KO KCs expressing PKP1-WT-EGFP (PKP1-rescue), PKP1-4xA-EGFP or EGFP were seeded in LCM and treated as shown in Fig. S1A. Cells were lysed in IP buffer (20 mM Tris-HCl (pH 7.5), 137 mM NaCl, 10% glycerol, and 1% IGEPAL CA-630 supplemented with 1x Halt^TM^ protease and phosphatase inhibitor cocktail). The lysates were cleared by centrifugation for 15 min at 4 °C and 13,000 *g*. One-tenth of the supernatant was mixed with SDS‒PAGE loading buffer and used as an input control. GFP-Trap agarose (ChromoTek, Germany) was incubated with the residual supernatant for 1 h at 4 °C on an overhead rotator and then washed four times in IP buffer, after which the bound proteins were eluted in SDS‒PAGE loading buffer. Input and eluate samples were separated by SDS‒PAGE and analyzed by WB.

GFP-Trap IPs for MS were carried out as described above and subsequently subjected to on-bead digestion. To this end, the beads were dried by removing the excess liquid using a gel loading tip. Beads were resuspended in 50 µl of digestion buffer I [5 mM tris(2-carboxyethyl)phosphine (TCEP), 27.5 mM chloroacetamide (CAA), 10 ng/µl lysyl-endopeptidase (LysC) in 6 M urea, 2 M thiourea and 50 mM ammonium bicarbonate (ABC)] and incubated for 5 h at RT. Afterward, each sample was diluted to 2 M urea/thiourea with 50 mM ABC and 10 ng/µl trypsin. The samples were incubated overnight at RT on a shaker at 1050 rpm. The supernatants were removed by a Hamilton pipette (G22S), acidified to 4% formic acid and cleared by centrifugation in a tabletop centrifuge at maximum speed at RT for 10 min. The cleared peptide solutions were desalted and stored on SDB-RPS stage tips until measurement.

### Immunoprecipitation of endogenous PKP1

For immunoprecipitation of endogenous PKP1, murine keratinocytes or HaCaT cells were treated as indicated and lysed in IP buffer, as described for the GFP-Trap experiments. The lysates were cleared by centrifugation for 15 min at 13,000 g and 4 °C. One-tenth of the supernatant was mixed with SDS‒PAGE loading buffer and used as an input control. The remaining lysate was incubated with anti-PKP1 guinea pig serum overnight at 4 °C on an overnight rotator. For HaCaT cells, preimmune guinea pig serum was used as a negative control. For murine keratinocytes, PKP1-KO keratinocytes served as a specificity control. Immune complexes were captured using Protein A agarose beads (Thermo Scientific), washed four times with IP buffer, and eluted in SDS‒PAGE loading buffer. Input and eluate samples were separated by SDS‒PAGE and analyzed by Western blotting.

### Mass spectrometry and data analysis

The samples were eluted using 2% ammonia solution and 80% acetonitrile (ACN), dried and resuspended in 10 µl of 2% ACN and 5% formic acid. The samples were analyzed on a Thermo Orbitrap Q Exactive Plus coupled to a Thermo easy nLC. To this end, 2 µl of the samples were separated on a 1 h reversed-phase gradient during measurement with a 70k MS resolution with a 20 ms injection time and a 35k MSMS resolution with a 120 ms injection time. The resulting raw files were analyzed using MaxQuant (v1.6.14.0) and a UniProt (SwissProt and Tremble) mouse database, including isoforms (release 2022-02), using standard parameters with the following exceptions: Use label-free quantification (MaxLFQ), match between runs set true, and discard unmodified counterpart peptide sets to false. The MaxQuant output was further visualized by Perseus (v1.6.14.0) and InstantClue (v.1.11.2) [74–76].

### Quantification of mRNA levels

KCs were homogenized in TRIzol, and RNA was purified by phenol/chloroform extraction and isopropanol precipitation. cDNA synthesis was carried out using 1 µg of total RNA, random hexamer primers and SuperScript® II reverse transcriptase (Thermo Fisher Scientific) following the manufacturer’s protocol. Quantitative real-time PCR (qRT‒PCR) was performed with primaQUANT 2x qPCR CYBR Green MasterMix w/o ROX (Steinbrenner-Laborsysteme GmbH, Germany) using a LightCycler 480 II Real Time PCR system (Roche, Switzerland) with the following PCR conditions: 95 °C for 15 min followed by 40 cycles of 95 °C for 15 s, 62 °C for 15 s, and 72 °C for 20 s. Primer pairs (Table S5) were selected using Primer Blast (https://www.ncbi.nlm.nih.gov/tools/primer-blast/). The relative abundance of mRNAs was determined by the ΔΔC_t_ method and calculated by the following formula: fold change = (*E*_target_) ^∆CT^_target_ / (*E*_reference_) ^∆CT^_reference_. *Eef2* mRNA was used as a reference.

### RNA sequencing and data processing

Poly(A) mRNA sequencing was performed on three independent biological replicates of mock- and pIC-transfected WT, PKP1-KO and PKP1-rescue keratinocytes 6 h after pIC transfection. Strand-specific paired-end RNA sequencing was conducted by LC Sciences (Houston, TX, USA) using an Illumina NovaSeq 6000 platform, resulting in 150 bp long reads with an average of 2 × 36 million reads per sample. The quality of the raw fastq files was assessed using FastQC (https://www.bioinformatics.babraham.ac.uk/projects/fastqc/, v0.11.9). Sequencing adapters and low-quality read ends were clipped off using Cutadapt v2.8. The processed sequencing reads were aligned to the mouse genome (UCSC mm39) using HISAT2 v2.1.0. Alignments in the obtained BAM files were sorted, indexed and secondary alignments were filtered out using Samtools v1.10. FeatureCounts v2.0.0 was used to summarize the gene-mapped reads. Ensembl (GRC39.110) was used as the annotation basis. Differential gene expression was determined using the R package edgeR v4.0.1 with trimmed mean M value normalization. A false discovery rate (FDR)-adjusted *p* value less than 0.05 was considered to indicate significant differential gene expression. For these analyses, previously reported standard pipelines were used [77].

### Gene set enrichment analyses

Gene set enrichment analyses (GSEAs) were performed with the GSEA software (version 4.3.3) provided by the Broad Institute and MsigDB gene sets (v2024.1) [78–80] using the log2-fold change in mRNA expression, as derived from RNA-seq, for ranking. The data were analyzed using the classic scoring mode.

### Analysis of human LUSC transcriptomic data

For analysis of human lung squamous cell carcinoma, transcriptomic data from 550 patient-derived LUSC samples were obtained from the TCGA “Mixed Lung Squamous Cell Carcinoma v32” dataset. Genes were correlated with PKP1 expression across all samples, and genes whose expression was significantly correlated were selected when p ≤ 0.05. For GSEA, genes were ranked by the inverse correlation coefficient with PKP1 expression to identify pathways enriched in PKP1-low tumors. Enriched gene sets were compared with those obtained from pIC-stimulated PKP1-KO KCs.

### Functional enrichment analysis

A functional enrichment analysis was conducted using g:Profiler [81] via the g:GOSt tool (https://biit.cs.ut.ee/gprofiler/gost) to identify overrepresented biological processes and pathways among putative PKP1 interaction partners identified by mass spectrometry after PKP1-GFP-Trap or IP of endogenous PKP1 (published by [44]).

### Immunofluorescence analysis and image processing

WT, PKP1-KO and PKP1-rescue KCs were seeded on Ø 12-mm collagen I-coated glass coverslips and pIC-transfected as previously described. Six hours after transfection, the cells were fixed for 10 min in 3.7% formaldehyde in PBS at RT, permeabilized in 0.5% Triton X-100/PBS for 15 min at RT, and blocked in 1% BSA/PBS for 30 min at RT. p65 and IRF3 primary antibodies (Table S3) were diluted in blocking solution and incubated overnight at 4 °C in a humid chamber. The next day, the coverslips were washed 3x for 5 min in PBS, briefly blocked, and incubated for 1 h at RT with a Cy3 secondary antibody (Table S4) and Hoechst 33342 (Thermo Fisher Scientific) for DNA staining. Finally, the coverslips were washed 3x for 5 min in PBS, desalted in ddH_2_O, dehydrated in ethanol and mounted in Mowiol [5% Mowiol, 30% glycerol, 0.25% 1,4-diazabicyclo(2.2.2)octane (DABCO)]. Images were acquired using an SP8X confocal microscope (Leica) equipped with a white light laser and a 40×/0.95 PLAN APO objective. For comparisons between cell lines, samples were treated in parallel, and identical acquisition settings were used across all samples. Fiji software was used for image processing [73]. For image processing, only linear adjustments of brightness and contrast were applied to improve clarity. No further modifications or alterations of the image data were performed.

### Proximity ligation assay (PLA)

WT and PKP1-KO keratinocytes were treated exactly as described for immunofluorescence analysis. After the primary antibodies (anti-PKP1-mouse together with anti-DDX3X-, anti-DDX21- or anti-RIG-I-rabbit/PKP1-rabbit together with anti-DDX1- or anti-DHX15-mouse, see Table S3) were incubated, the DuoLink assay (Sigma‒Aldrich) was carried out following the manufacturer’s protocol. Images were acquired using an SP8X confocal microscope (Leica) equipped with a white light laser and a 63×/1.4 NA oil immersion objective. Z-stacks were collected with a step size of 0.5 µm, and identical acquisition settings were used across all samples of a specific antibody combination. Raw confocal images were imported into Fiji/ImageJ (version 2.14.0), and maximum intensity projections were generated from the z-stacks for subsequent quantification. PLA signals were identified using the “Analyze Particles” function following consistent thresholding (manually set value 50–255) to isolate discrete puncta. Size and circularity filters were applied (4–200 pxl², circularity 0.5–1.0) to exclude nonspecific noise. Nuclei were segmented using DAPI staining and manually counted to calculate the average number of PLA signals per cell. In total, five images per condition from two independent experiments were used for quantification.

### Quantification and statistical analysis

#### WB quantification

To quantify the immunoblots, we used the “Gel Analysis” tool in ImageJ. All signals were normalized to the internal loading controls β-actin or α-tubulin. Details concerning the quantification procedures are given in the figure legends.

#### Quantification of immunofluorescence

To quantify the nuclear accumulation of p65 and IRF3 in KCs, the nuclei were segmented in the Hoechst 33342 channel using Fiji/ImageJ. The resulting nuclear masks were transferred to the corresponding p65 or IRF3 fluorescence channels, and the mean nuclear fluorescence intensity was measured for each nucleus. To determine the corresponding non-nuclear background/cytoplasmic signal, nuclear regions were excluded from the image, and the mean fluorescence intensity of the remaining area was measured in the respective p65 or IRF3 channel. Nuclear enrichment was calculated by dividing the mean nuclear fluorescence intensity of each cell by the mean non-nuclear fluorescence intensity of the corresponding image. Mitotic, polynuclear, overlapping or poorly segmented cells were excluded from the analysis. At least 900 cells were analyzed per condition from three independent experiments.

### Statistical analysis

Statistical analysis and graph preparation were performed using GraphPad Prism software version 8.3. Individual data points are shown in the graphs where applicable. Data are presented as the mean ± SD unless stated otherwise in the corresponding figure legend. The number of biological replicates, analyzed cells and analyzed images are indicated in the figure legends. For comparisons of two groups, statistical significance was determined using an unpaired two-tailed Student’s *t* test. For comparisons of more than two groups, one-way ANOVA followed by Tukey’s multiple comparison test was used. For nonnormally distributed single-cell fluorescence intensity data, statistical significance was determined using the nonparametric Kruskal‒Wallis test followed by multiple comparison analysis, as indicated in the figure legend. Significance levels are indicated as follows: n.s. = not significant; * *p* < 0.05; ** *p* < 0.01; *** *p* < 0.001.

## Supplementary information

Supplementary Data

Uncropped blots and images

## Data Availability

All the data are available in the main text or the supplementary materials. The raw RNA-seq data generated in this study have been deposited in the National Center for Biotechnology Information Gene Expression Omnibus (GEO) database (https://www.ncbi.nlm.nih.gov/geo) under the GEO series accession number GSE300261. The mass spectrometry proteomics data have been deposited to the ProteomeXchange Consortium (https://www.ebi.ac.uk/pride/login) via the PRIDE partner repository with the dataset identifier PXD065254.
