# Supplementary material for: Plakophilin 1 suppresses keratinocyte innate immune responses through DExD/H helicases
Source: Cell Mol Immunol. 2026 Aug 19;23(10):1269–84. doi: 10.1038/s41423-026-01462-3 (PMC13623832; doi:10.1038/s41423-026-01462-3)
Supplement: Supplementary file 1 [file 41423_2026_1462_MOESM1_ESM.pdf]

Figure S1

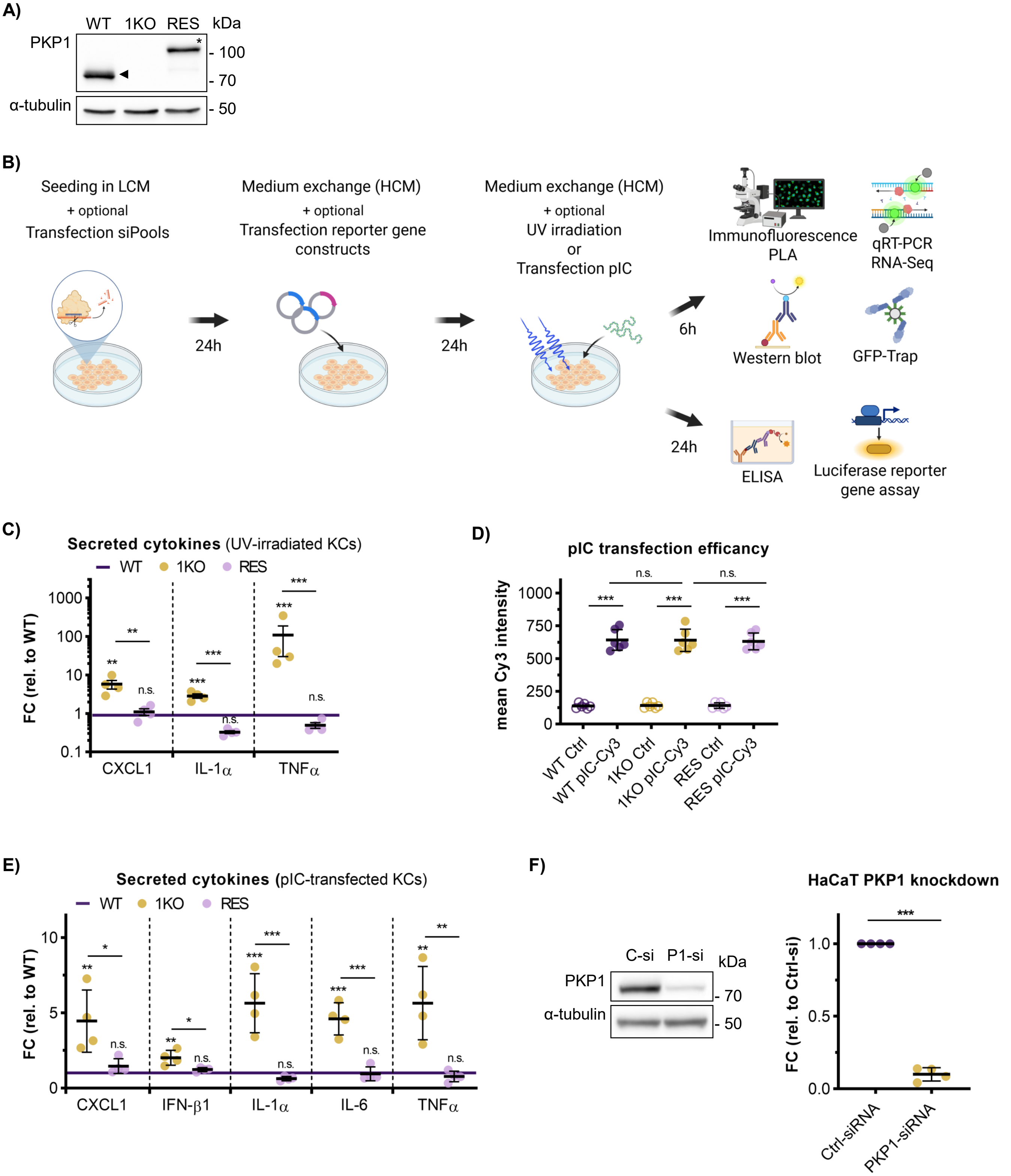
A)
WT 1KO RES kDa
PKP1
- 100
- 70
α-tubulin
- 50
B)
Seeding in LCM
+ optional
Transfection siPools
24h
Medium exchange (HCM)
+ optional
Transfection reporter gene constructs
24h
Medium exchange (HCM)
+ optional
UV irradiation
or
Transfection pIC
6h
24h
Immunofluorescence
PLA
qRT-PCR
RNA-Seq
Western blot
GFP-Trap
ELISA
Luciferase reporter gene assay
C)
Secreted cytokines (UV-irradiated KCs)
WT 1KO RES
FC (rel. to WT)
CXCL1
IL-1α
TNFα
D)
pIC transfection efficancy
mean Cy3 intensity
WT Ctrl
WT pIC-Cy3
1KO Ctrl
1KO pIC-Cy3
RES Ctrl
RES pIC-Cy3
E)
Secreted cytokines (pIC-transfected KCs)
WT 1KO RES
FC (rel. to WT)
CXCL1
IFN-β1
IL-1α
IL-6
TNFα
F)
HaCaT PKP1 knockdown
C-si P1-si kDa
PKP1
- 70
α-tubulin
- 50
FC (rel. to Ctrl-si)
Ctrl-siRNA
PKP1-siRNA

Figure S2

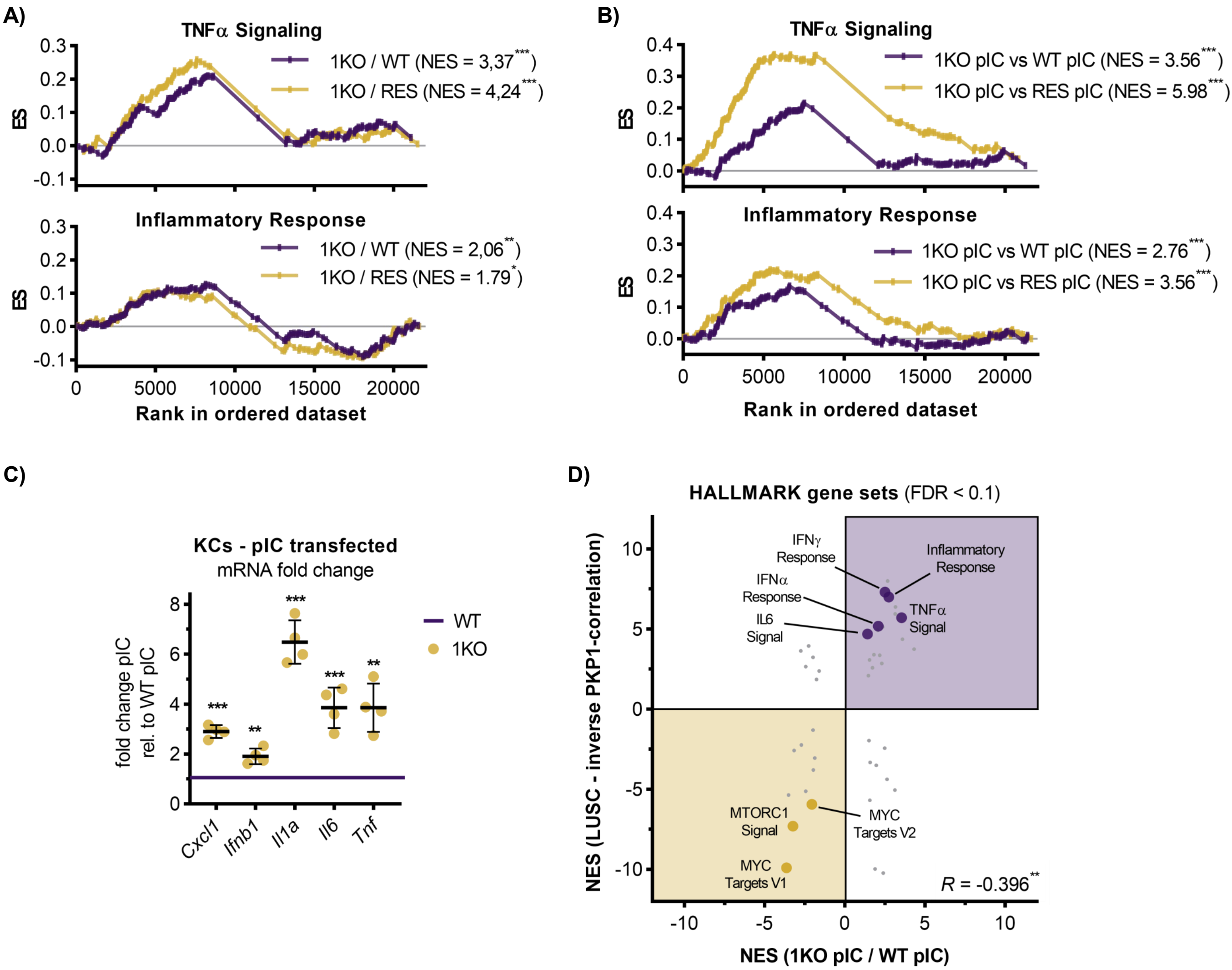
A)
TNFα Signaling
1KO / WT (NES = 3,37***)
1KO / RES (NES = 4,24***)
ES
Inflammatory Response
1KO / WT (NES = 2,06**)
1KO / RES (NES = 1.79*)
Rank in ordered dataset
B)
TNFα Signaling
1KO pIC vs WT pIC (NES = 3.56***)
1KO pIC vs RES pIC (NES = 5.98***)
Inflammatory Response
1KO pIC vs WT pIC (NES = 2.76***)
1KO pIC vs RES pIC (NES = 3.56***)
Rank in ordered dataset
C)
KCs - pIC transfected
mRNA fold change
fold change pIC rel. to WT pIC
WT
1KO
Cxcl1
Ifnb1
Il1a
Il6
Tnf
D)
HALLMARK gene sets (FDR < 0.1)
IFNγ Response
Inflammatory Response
IFNα Response
TNFα Signal
IL6 Signal
MTORC1 Signal
MYC Targets V2
MYC Targets V1
R = -0.396**
NES (LUSC - inverse PKP1-correlation)
NES (1KO pIC / WT pIC)

Figure S3

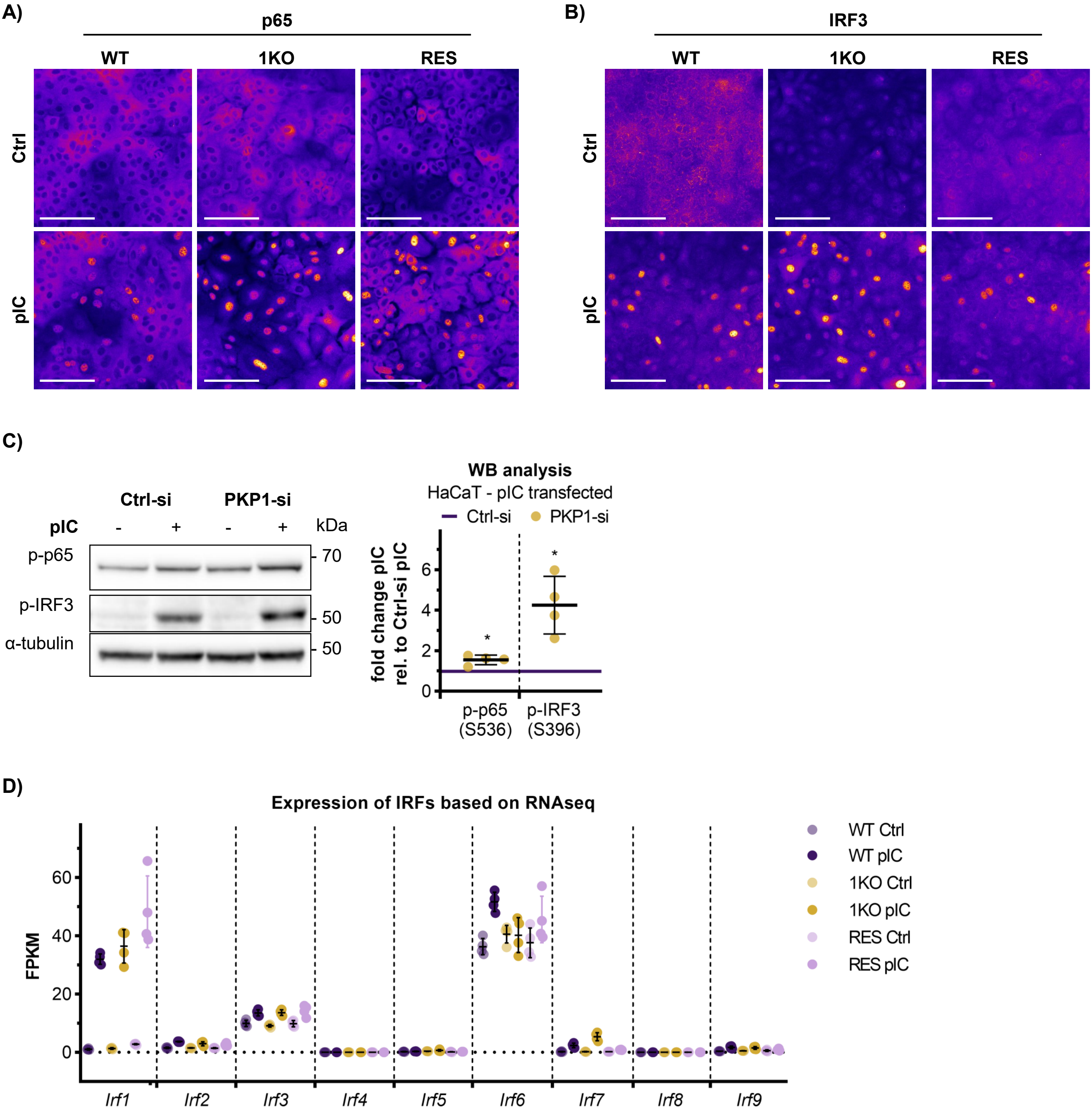

A)
p65
WT
1KO
RES
Ctrl
pIC
B)
IRF3
WT
1KO
RES
Ctrl
pIC
C)
Ctrl-si
PKP1-si
pIC
-
+
-
+
kDa
p-p65
70
p-IRF3
50
α-tubulin
50
WB analysis
HaCaT - pIC transfected
Ctrl-si
PKP1-si
fold change pIC rel. to Ctrl-si pIC
0
2
4
6
*
*
p-p65 (S536)
p-IRF3 (S396)
D)
Expression of IRFs based on RNAseq
FPKM
0
20
40
60
Irf1
Irf2
Irf3
Irf4
Irf5
Irf6
Irf7
Irf8
Irf9
WT Ctrl
WT pIC
1KO Ctrl
1KO pIC
RES Ctrl
RES pIC

Figure S4

A)

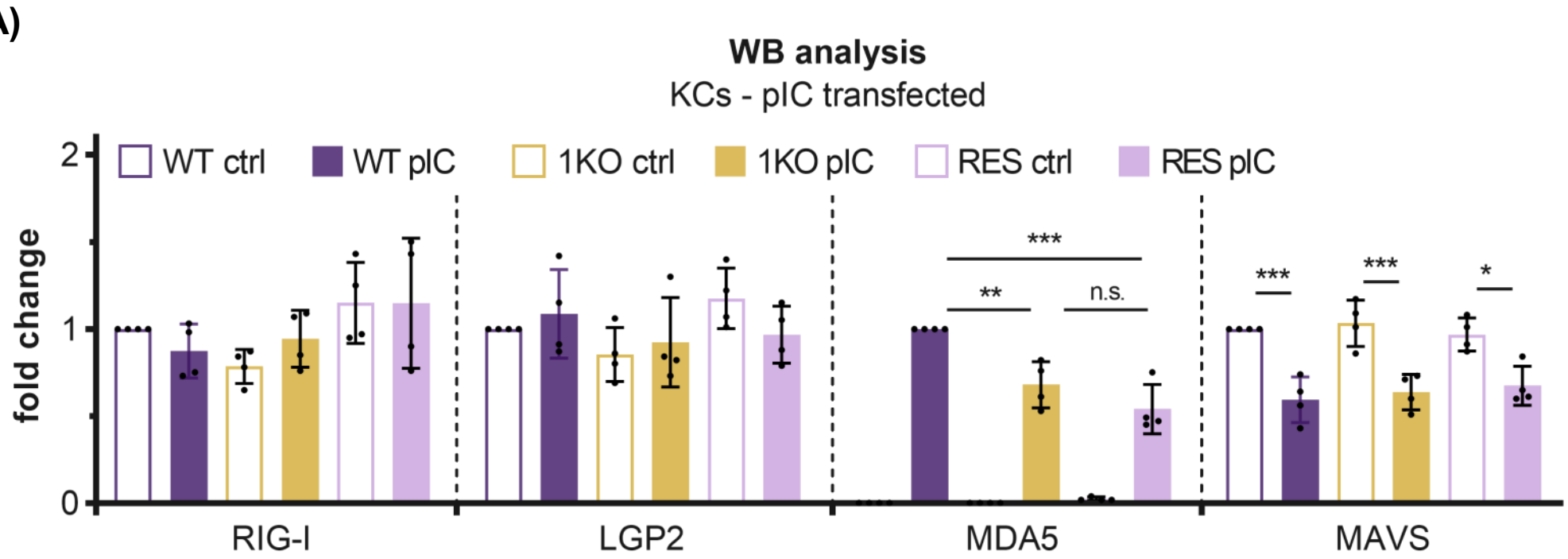

WB analysis
KCs - pIC transfected
WT ctrl
WT pIC
1KO ctrl
1KO pIC
RES ctrl
RES pIC
fold change
RIG-I
LGP2
MDA5
MAVS

B)

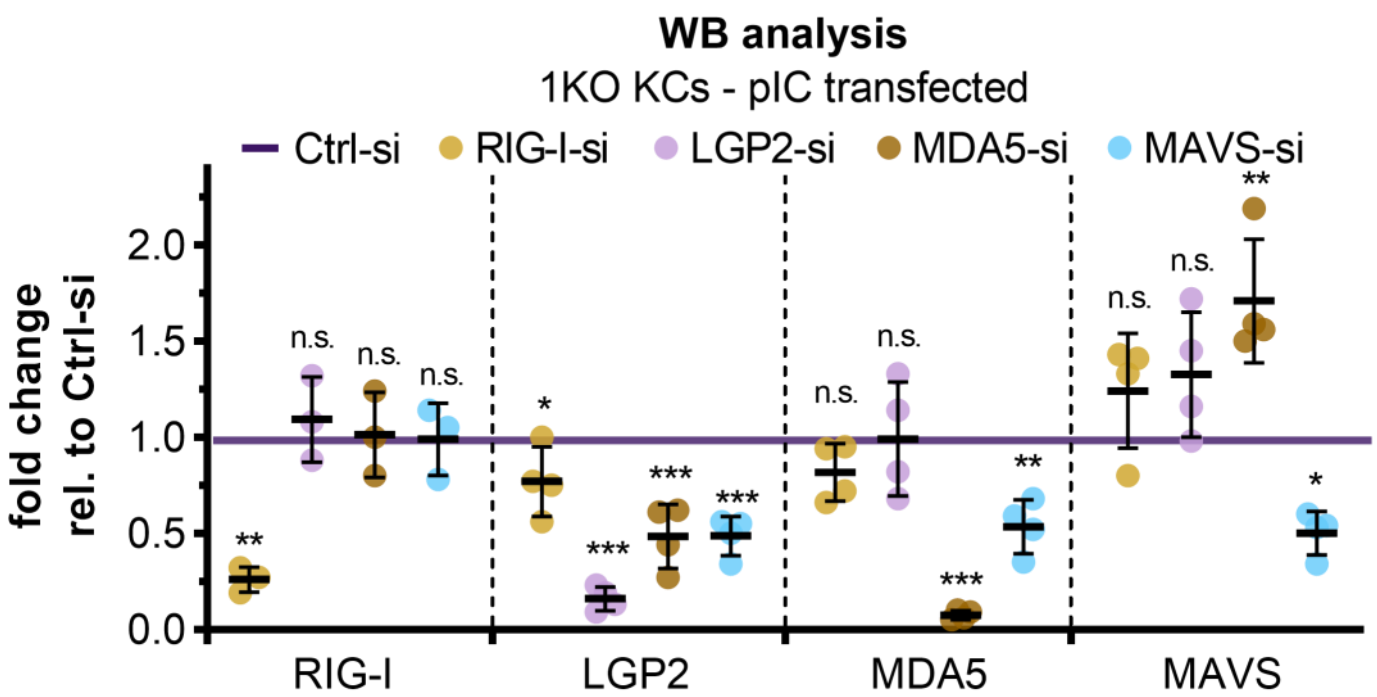

WB analysis
1KO KCs - pIC transfected
Ctrl-si
RIG-I-si
LGP2-si
MDA5-si
MAVS-si
fold change rel. to Ctrl-si
RIG-I
LGP2
MDA5
MAVS

Figure S5

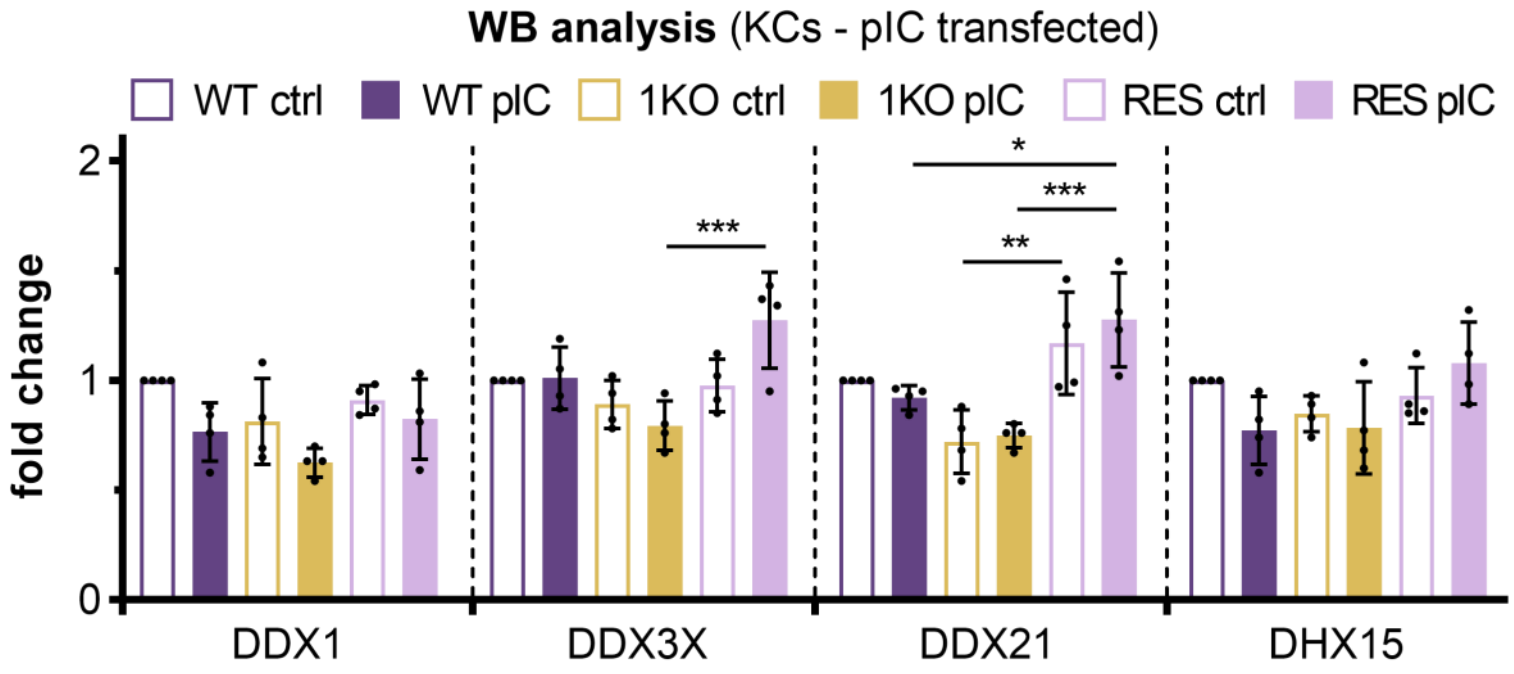

WB analysis (KCs - pIC transfected)
WT ctrl
WT pIC
1KO ctrl
1KO pIC
RES ctrl
RES pIC
fold change
0
1
2
*
***
**
***
DDX1
DDX3X
DDX21
DHX15

Figure S6

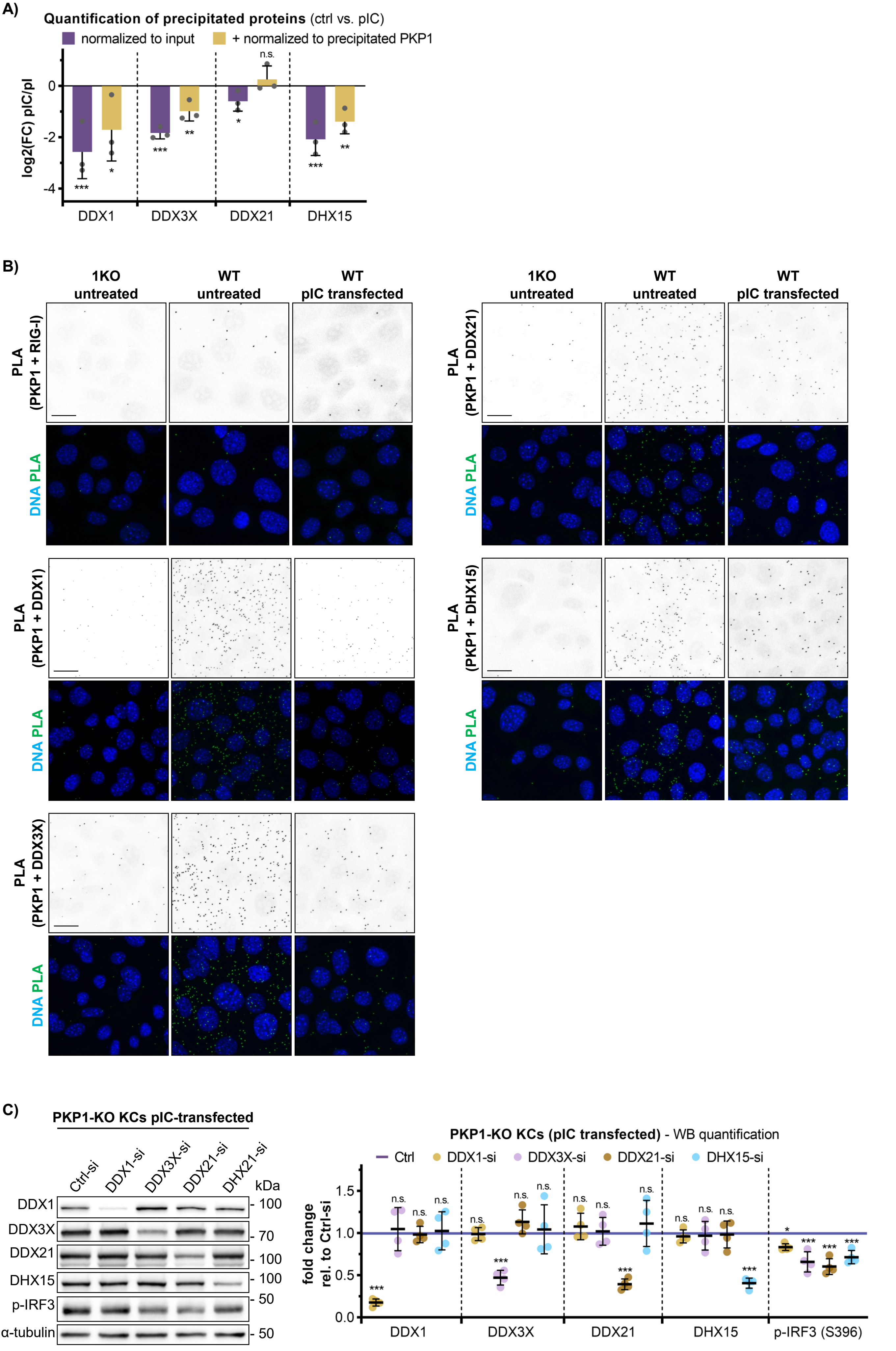

A)
Quantification of precipitated proteins (ctrl vs. pIC)
normalized to input
+ normalized to precipitated PKP1
log2(FC) pIC/pI
0
-2
-4
n.s.
***
*
***
**
*
***
**
DDX1
DDX3X
DDX21
DHX15
B)
1KO untreated
WT untreated
WT pIC transfected
PLA (PKP1 + RIG-I)
DNA PLA
PLA (PKP1 + DDX1)
DNA PLA
PLA (PKP1 + DDX3X)
DNA PLA
1KO untreated
WT untreated
WT pIC transfected
PLA (PKP1 + DDX21)
DNA PLA
PLA (PKP1 + DHX15)
DNA PLA
C)
PKP1-KO KCs pIC-transfected
Ctrl-si
DDX1-si
DDX3X-si
DDX21-si
DHX21-si
kDa
DDX1
- 100
DDX3X
- 70
DDX21
- 100
DHX15
- 100
p-IRF3
- 50
α-tubulin
- 50
PKP1-KO KCs (pIC transfected) - WB quantification
Ctrl
DDX1-si
DDX3X-si
DDX21-si
DHX15-si
fold change rel. to Ctrl-si
1.5
1.0
0.5
0.0
n.s.
***
*
DDX1
DDX3X
DDX21
DHX15
p-IRF3 (S396)

Figure S7

A)

PKP1 - phosphorylation sites

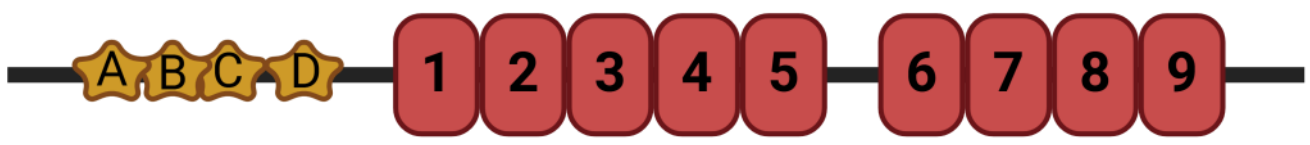

Motive A: RQKS^{54}K$S^{56}S^{57}$Q$S^{59}S^{60}T^{61}$LS^{63}HS^{65}NRGS^{69}
Motive B: DNYNYGT^{82}TS^{84}RSSYYS
Motive C: DNRRF$S^{118}S^{119}$YS^{121}QMENWS^{127}RHYPR
Motive D: RGT^{166}LRKGT^{171}LGS^{174}KGQK$T^{179}T^{180}$QNRYS^{185}FY$S^{188}T^{189}$CS^{191}G

B)

PKP1-WT-EGFP

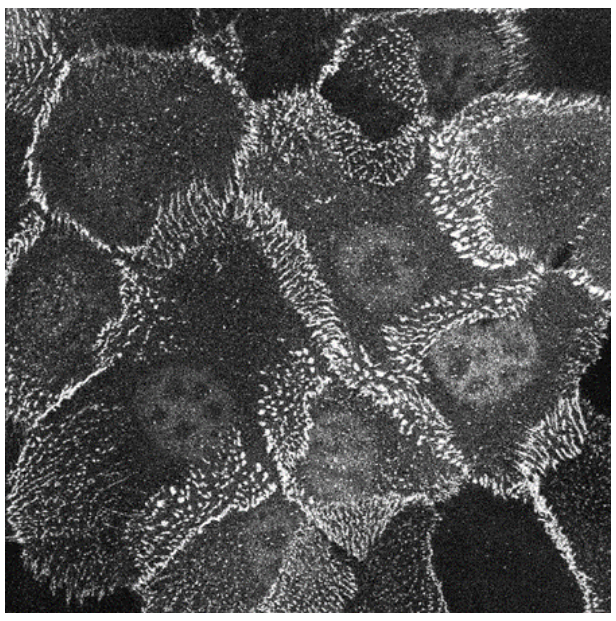

PKP1-4xA-EGFP

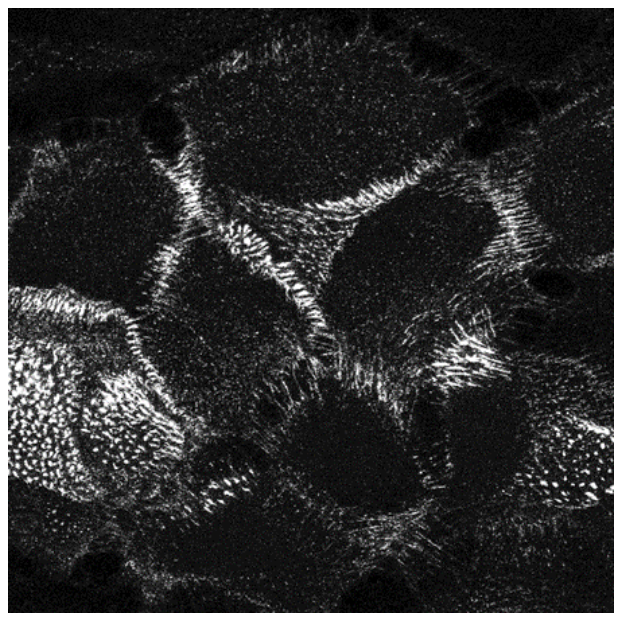

Figure S8

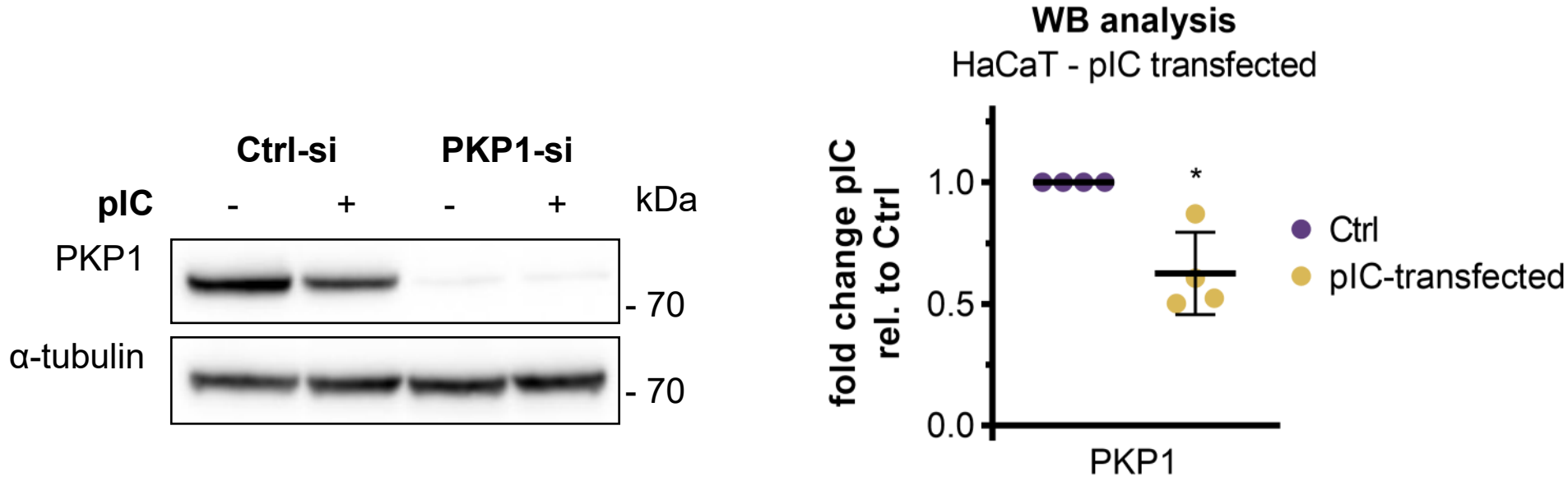
Ctrl-si
PKP1-si
pIC
-
+
-
+
kDa
PKP1
- 70
α-tubulin
- 70
WB analysis
HaCaT - pIC transfected
fold change pIC rel. to Ctrl
1.0
0.5
0.0
*
PKP1
Ctrl
pIC-transfected

Figure S1: Loss of PKP1 increases secretion of pro-inflammatory cytokines.

(A) Western blot analysis of WT, PKP1-KO (1KO) and PKP1-rescue (RES) KCs showing PKP1 expression. Endogenous PKP1 is indicated by an arrowhead, and PKP1-WT-EGFP expressed in RES KCs is marked by an asterisk. α-tubulin served as loading control.

(B) Schematic overview of the general experimental design used in this study. Created with BioRender.

(C) WT, 1KO and RES KCs were exposed to UVB irradiation, related to Fig. 1C. After 24 h, culture supernatants were collected and secreted CXCL1, IL-1α and TNFα were quantified by ELISA. Data show mean fold changes ± SD relative to UVB-treated WT KCs; n = 4.

(D) WT, 1KO and RES KCs were mock-transfected or transfected with Cy3-pIC. Cy3 fluorescence intensity was quantified by flow cytometry 6 h after transfection. Data show mean ± SD from three independent biological replicates performed in technical duplicates.

(E) WT, 1KO and RES KCs were transfected with pIC, related to Fig. 1D. After 24 h, culture supernatants were collected and secreted CXCL1, IFN-β1, IL-1α, IL-6 and TNFα were quantified by ELISA. Data show mean fold changes ± SD relative to pIC-transfected WT KCs; n = 4.

(F) Western blot analysis of HaCaT cells transfected with control or PKP1-directed siRNAs, related to Fig. 1E, F. Left: representative immunoblots of endogenous PKP1. α-tubulin served as loading control. Right: densitometric quantification of PKP1 levels normalized to α-tubulin. Data show fold changes ± SD relative to control siRNA-transfected cells; n = 4.

Statistical significance was determined by unpaired two-tailed Student’s t-test (F) or one-way ANOVA with Tukey’s multiple comparison test (C-E). n.s. = not significant; * $p < 0.05$; ** $p < 0.01$; *** $p < 0.001$

Figure S2: PKP1 globally affects inflammatory and innate immune gene expression.

(A) Enrichment plots of the indicated gene sets determined by GSEA using gene lists ranked by fold change in untreated PKP1-KO (1KO) versus WT KCs or untreated 1KO versus PKP1-rescue (RES) KCs, related to Fig. 2A. ES, enrichment score.

(B) Enrichment plots of the indicated gene sets determined by GSEA using gene lists ranked by fold change in pIC-transfected 1KO versus WT KCs or pIC-transfected 1KO versus RES KCs, related to Fig. 2C.

(C) qRT-PCR analysis of proinflammatory cytokine mRNA levels in WT and 1KO KCs after pIC transfection, related to Fig. 2D. Eef2 was used as reference gene. Data show mean mRNA fold changes ± SD relative to pIC-transfected WT KCs; n = 4.

(D) XY plot of NES for Hallmark gene sets comparing pIC-transfected 1KO versus WT KCs (x-axis) and gene sets enriched in LUSC samples with inverse PKP1 correlation (y-axis), related to Fig. 2F.

Statistical significances were determined by one-way ANOVA with Tukey's multiple comparison test; ** $p < 0.01$; *** $p < 0.001$.

Figure S3: PKP1 represses NFκB and IRF transcription factor activation.

(A, B) Immunofluorescence analysis of mock- and pIC-transfected WT, PKP1-KO (1KO) and PKP1-rescue (RES) KCs showing localization of p65 (A) and IRF3 (B), related to Fig. 3D, E. Representative uncropped images are shown. Scale bars, 100 µm.

(C) Western blot analysis of control and PKP1-siRNA-transfected HaCaT cells 6 h after mock or pIC transfection, related to Fig. 3C. Left: representative immunoblots of phosphorylated p65 and phosphorylated IRF3. α-tubulin served as loading control. Right: densitometric quantification of phospho-protein levels normalized to α-tubulin. Data show fold changes ± SD in pIC-transfected PKP1-knockdown cells relative to pIC-transfected control cells; n = 4.

(D) Expression of IRF family members in murine KCs based on RNA-seq data, related to Fig. 4B. FPKM values are shown for mock- and pIC-transfected WT, 1KO and RES KCs.

Statistical significances were determined by paired t test. * $p < 0.05$

Figure S4: Western blot analysis of RLR signaling pathway components.

(A) Densitometric quantification of RIG-I, LGP2, MDA5 and MAVS protein levels from the Western blot analysis shown in Fig. 5B. Protein levels were normalized to α-tubulin. RIG-I, LGP2 and MAVS values are shown relative to mock-transfected WT KCs, whereas MDA5 values are shown relative to pIC-transfected WT KCs; n = 4.

(B) Densitometric quantification of RLR pathway component knockdown efficiencies from the Western blot analysis shown in Fig. 5C. Protein levels were normalized to α-tubulin and are shown relative to control siPool-transfected 1KO KCs; n = 4.

Statistical significances were determined by one-way ANOVA with Tukey's multiple comparison test. n.s. = not significant; * $p < 0.05$; ** $p < 0.01$; *** $p < 0.001$.

Figure S5: Quantitative analysis of DExD/H-box helicase expression.

Densitometric quantification of DDX1, DDX3X, DDX21 and DHX15 protein levels from the Western blot analysis shown in Fig. 6D. Protein levels were normalized to α-tubulin and are shown relative to mock-transfected WT KCs; n = 4.

Statistical significances were determined by one-way ANOVA with Tukey's multiple comparison test. n.s. = not significant; * $p < 0.05$; ** $p < 0.01$; *** $p < 0.001$.

Figure S6: PKP1 sequesters DExD/H-box helicases.

(A) Densitometric quantification of DExD/H-box helicases precipitated in GFP-Trap experiments shown in Fig. 7C. Precipitated helicase levels were normalized either to the corresponding input

levels or to precipitated PKP1-RES to account for reduced PKP1 levels after pIC transfection. Data show log2 fold changes comparing pIC-transfected with mock-transfected cells.

(B) Representative DuoLink proximity ligation assay images showing proximity between endogenous PKP1 and the indicated proteins, related to Fig. 7D. Untreated PKP1-KO (1KO) KCs served as specificity control. Untreated WT KCs and pIC-transfected WT KCs were used to monitor stimulus-dependent changes in PKP1-helicase proximity. Nuclei were stained with DAPI. Scale bars, 20 µm.

(C) Validation of DExD/H-box helicase knockdown efficiency, related to Fig. 7E, F. Left: representative immunoblots of 1KO KCs transfected with the indicated siPools prior to pIC stimulation. α-tubulin served as loading control. Right: densitometric quantification of DDX1, DDX3X, DDX21, DHX15 and p-IRF3 levels normalized to α-tubulin. Data show fold changes ± SD relative to control siPool-transfected 1KO KCs; $n = 4$.

Statistical significances were determined by one-way ANOVA with Tukey's multiple comparison test. n.s. = not significant; * $p < 0.05$; ** $p < 0.01$; *** $p < 0.001$.

Figure S7: A PKP1 mutant covering several phosphorylation sites localizes to desmosomes.

(A) Schematic overview of PKP1 head-domain phosphorylation motifs, related to Fig. 8A-C. The four indicated motifs contain serine and threonine residues previously shown to regulate PKP1 localization through phosphorylation.

(B) Fluorescence images of PKP1-KO KCs expressing PKP1-WT-EGFP or PKP1-4xA-EGFP after 24 h incubation in high calcium medium, related to Fig. 8A-C. Substitution of serine and threonine residues within the motifs shown in (A) by alanine restricts PKP1-4xA-EGFP predominantly to cell-cell contacts.

Figure S8: pIC stimulation triggers the reduction of endogenous PKP1 levels in HaCaT cells.

Western blot analysis of HaCaT cells transfected with control siRNA or PKP1-directed siRNA followed by mock or pIC transfection, related to Fig. 8D. Left: representative immunoblots of endogenous PKP1. α-tubulin served as loading control. Right: densitometric quantification of PKP1 protein levels in control siRNA-transfected HaCaT cells after pIC stimulation. Protein levels were normalized to α-tubulin and are shown relative to mock-transfected control cells; $n = 4$ Statistical significance was determined by paired two-tailed Student's t-test. * $p < 0.05$.

Table S1: List of used siPools (defined pools of 30 selected siRNAs) obtained from siTools Biotech GmbH

Target	NCBI Gene ID
non-targeting control	N/A
human *PKP1*	5317
murine *Ddx1*	104721
murine *Ddx3x*	13205
murine *Dhx15*	13204
murine *Ddx21*	56200
murine *Rela* (p65)	19697
murine *Irf3*	54131
murine *Mavs*	228607
murine *Ifih1* (MDA5)	71586
murine *Dhx58* (LGP2)	80861
murine *Ddx21*	56200
murine *Rigi* (RIG-I, DDX58)	230073

Table S2: List of used ELISA kits

ELISA-Kit	Source	Identifier
ELISA MAX™ Standard Set Mouse IL-1α	BioLegend	Cat# 433401
ELISA MAX™ Standard Set Mouse IL-6	BioLegend	Cat# 431301
ELISA MAX™ Standard Set Mouse TNF-α	BioLegend	Cat# 430901
ELISA MAX™ Standard Set Human IL-6	BioLegend	Cat# 430501
ELISA MAX™ Standard Set Human TNF-α	BioLegend	Cat# 430201
Mouse CXCL1/KC DuoSet ELISA	R&D Systems	Cat# DY453-05
Mouse IFN-beta DuoSet ELISA	R&D Systems	Cat# DY8234-05

Table S3: Primary antibodies including dilutions in Western Blot (WB), immunofluorescence (IF), immunoprecipitation (IP) or proximity ligation assay (PLA)

Antibody	Source	Identifier	Dilution
c-Myc (D84C12) Rabbit mAb	Cell Signaling Technology	RRID: AB_1903938	1:1000 (WB)
DDX1 (A-7) Mouse mAb	Santa Cruz Biotechnology	RRID: AB_10650122	1:500 (WB)<br>1:250 (PLA)
DDX21 Rabbit pAb	Cell Signaling Technology	RRID: n.a.<br>Cat# 75762	1:1000 (WB)<br>1:500 (PLA)
DDX3 (D19B4) Rabbit mAb	Cell Signaling Technology	RRID: AB_10860416	1:1000 (WB)<br>1:500 (PLA)
DHX15 (E-6) Mouse mAb	Santa Cruz Biotechnology	RRID: AB_10707990	1:1000 (WB)<br>1:500 (PLA)
GFP Rabbit pAb	Rockland	RRID: AB_828167	1:2000 (WB)
IRF-3 (D83B9) Rabbit mAb	Cell Signaling Technology	RRID: AB_1904036	1:500 (WB)<br>1:250 (IF)
LGP2 Rabbit pAb	Thermo Fisher Scientific	RRID: AB_2855046	1:1000 (WB)
MAVS (E8Z7M) Rabbit mAb #83000	Cell Signaling Technology	RRID: AB_2927715	1:2000 (WB)
MDA-5 (D74E4) Rabbit mAb #5321	Cell Signaling Technology	RRID: AB_10694490	1:1000 (WB)
NF-κB p65 (D14E12) XP® Rabbit mAb #8242	Cell Signaling Technology	RRID: AB_10859369	1:2000 (WB)<br>1:1000 (IF)
Phospho-IKK alpha/beta (Ser176/180) (16A6) Rabbit mAb #2697	Cell Signaling Technology	RRID: n.a.<br>Cat# 2697	1:1000 (WB)
Phospho-IRF-3 (Ser396) (D6O1M) Rabbit mAb #29047	Cell Signaling Technology	RRID:AB_2773013	1:500 (WB)
Phospho-NF-κB p65 (Ser536) (93H1) Rabbit mAb #3033	Cell Signaling Technology	RRID: AB_331284	1:1000 (WB)
PKP1 (10B2) Mouse mAb #sc-33636	Santa Cruz Biotechnology	RRID: AB_2164139	1:1000 (WB)
PKP1 guinea pig (serum)	Hatzfeld Lab	(1)	1:200 (IP)
PKP1 Rabbit (serum)	Hatzfeld Lab	(2, 3)	1:1000 (PLA)
RIG-I (D14G6) Rabbit mAb #3743	Cell Signaling Technology	RRID: AB_2269233	1:1000 (WB)<br>1:500 (PLA)
β-actin Mouse mAb #A2228	Sigma-Aldrich	RRID: AB_476697	1:2000 (WB)
α-tubulin Mouse mAb	Sigma-Aldrich	RRID: AB_2617116	1:2000 (WB)

Table S4: List of secondary antibodies including dilutions in immunoblot (IB) and immunofluorescence (IF)

Antibody	Source	Identifier	Dilution
Peroxidase-AffiniPure Donkey Anti-Mouse IgG	Jackson ImmunoResearch Labs	RRID: AB_2340770	1:20,000 (WB)
Peroxidase-AffiniPure Donkey Anti-Rabbit IgG	Jackson ImmunoResearch Labs	RRID: AB_10015282	1:40,000 (WB)
Cy3 AffiniPure F(ab')$_2$ Fragment Donkey Anti-Rabbit IgG	Jackson ImmunoResearch Labs	RRID: AB_2313568	1:500 (IF)
IRDye® 800CW Donkey anti-Mouse IgG	LI-COR Biotech	RRID: AB_2716622	1:15000 (WB)

Table S5: Sequences of primers (FW = forward primer, REV = reverse primer) used in qRT-PCR

Primer	Sequence 5' --> 3'
Eef2-mouse FW	ATGAGGCCGCCATGGGTATTA
Eef2-mouse REV	TAGTTGGGGCCCATGATCCG
Cxcl1-mouse FW	AAGAATGGTCGCGAGGCTTG
Cxcl1-mouse REV	GTGTTGTCAGAAGCCAGCGT
Il1a-mouse FW	GTCAACTCATTGGCGCTTGA
Il1a-mouse REV	TGCAAGTCTCATGAAGTGAGC
Il6-mouse FW	AACGATGATGCACTTGCAGAAA
Il6-mouse REV	TGGTACTCCAGAAGACCAGAG
Tnf-mouse FW	GCCTATGTCTCAGCCTCTTCTC
Tnf-mouse REV	AGGGTCTGGGCCATAGAACTG
Ifnb1-mouse FW	CGTGGGAGATGTCCTCAACT
Ifnb1-mouse REV	CTGAAGATCTCTGCTCGGACC

1. Rietscher K, Wolf A, Hause G, Rother A, Keil R, Magin TM, et al. Growth Retardation, Loss of Desmosomal Adhesion, and Impaired Tight Junction Function Identify a Unique Role of Plakophilin 1 In Vivo. J Invest Dermatol. 2016;136:1471–8.
2. Hatzfeld M, Haffner C, Schulze K, Vinzens U. The function of plakophilin 1 in desmosome assembly and actin filament organization. J Cell Biol. 2000;149:209–22.
3. Wolf A, Krause-Gruszczynska M, Birkenmeier O, Ostareck-Lederer A, Huttelmaier S, Hatzfeld M. Plakophilin 1 stimulates translation by promoting eIF4A1 activity. J Cell Biol. 2010;188:463–71.
