# Supplementary material for: Plakophilin 1 suppresses keratinocyte innate immune responses through DExD/H helicases
Source: Cell Mol Immunol. 2026 Aug 19;23(10):1269–84. doi: 10.1038/s41423-026-01462-3 (PMC13623832; doi:10.1038/s41423-026-01462-3)

Fig. 3C

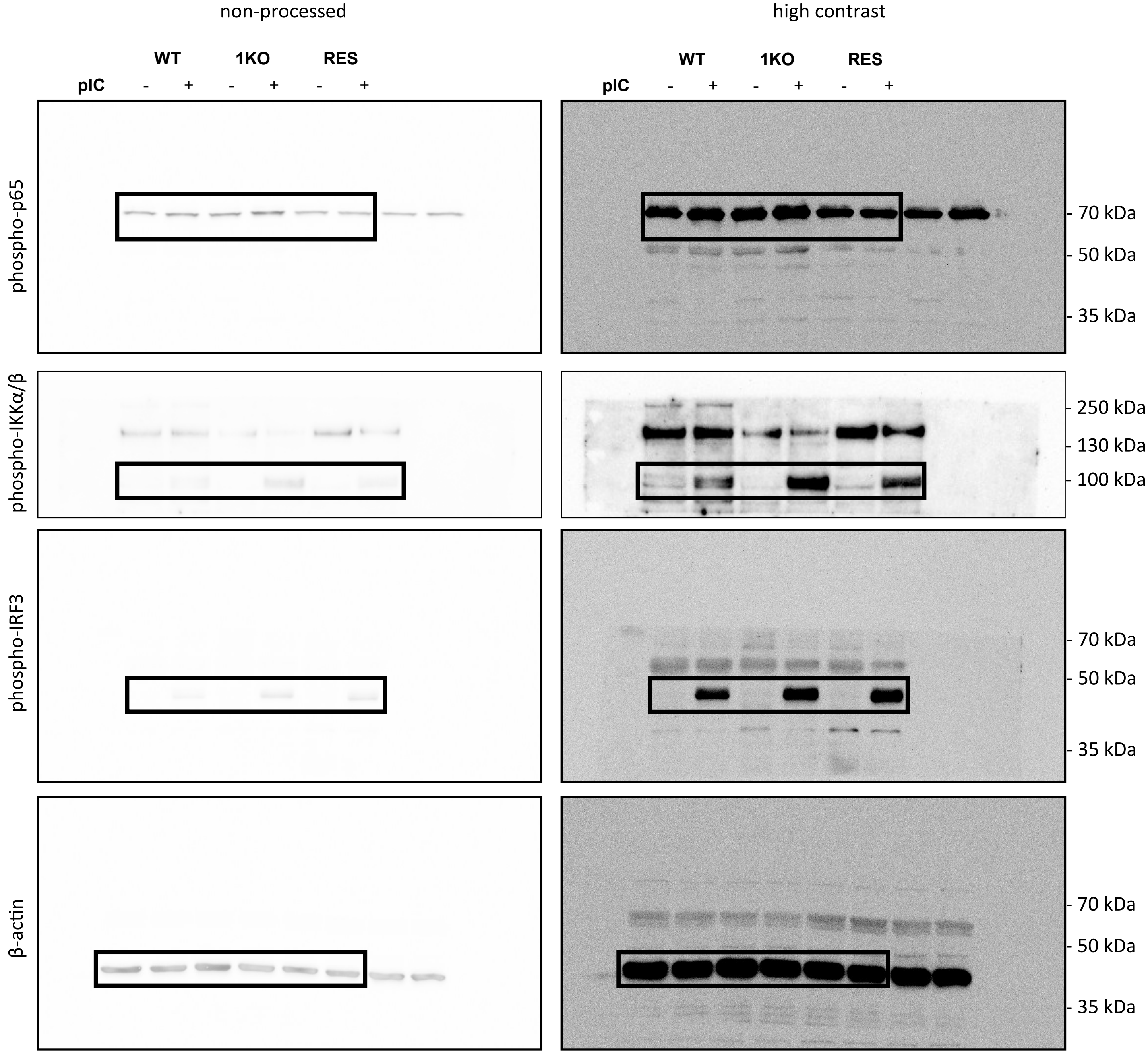
non-processed
high contrast
WT
1KO
RES
pIC
- + - + - +
phospho-p65
phospho-IKKα/β
phospho-IRF3
β-actin
70 kDa
50 kDa
35 kDa
250 kDa
130 kDa
100 kDa

Fig. 4A

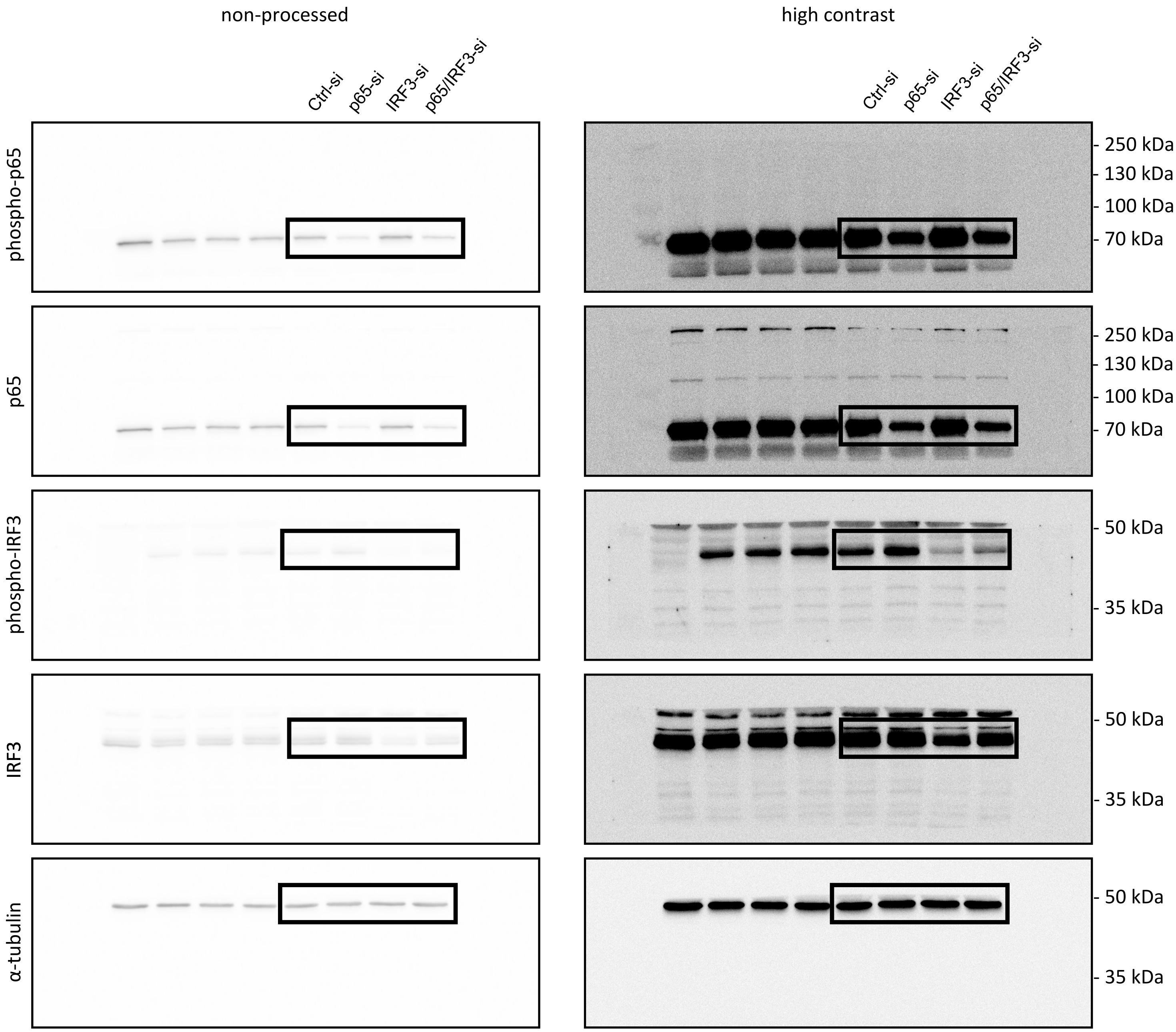

non-processed
high contrast
Ctrl-si
p65-si
IRF3-si
p65/IRF3-si
phospho-p65
p65
phospho-IRF3
IRF3
α-tubulin
250 kDa
130 kDa
100 kDa
70 kDa
250 kDa
130 kDa
100 kDa
70 kDa
50 kDa
35 kDa
50 kDa
35 kDa
50 kDa
35 kDa

Fig. 5B

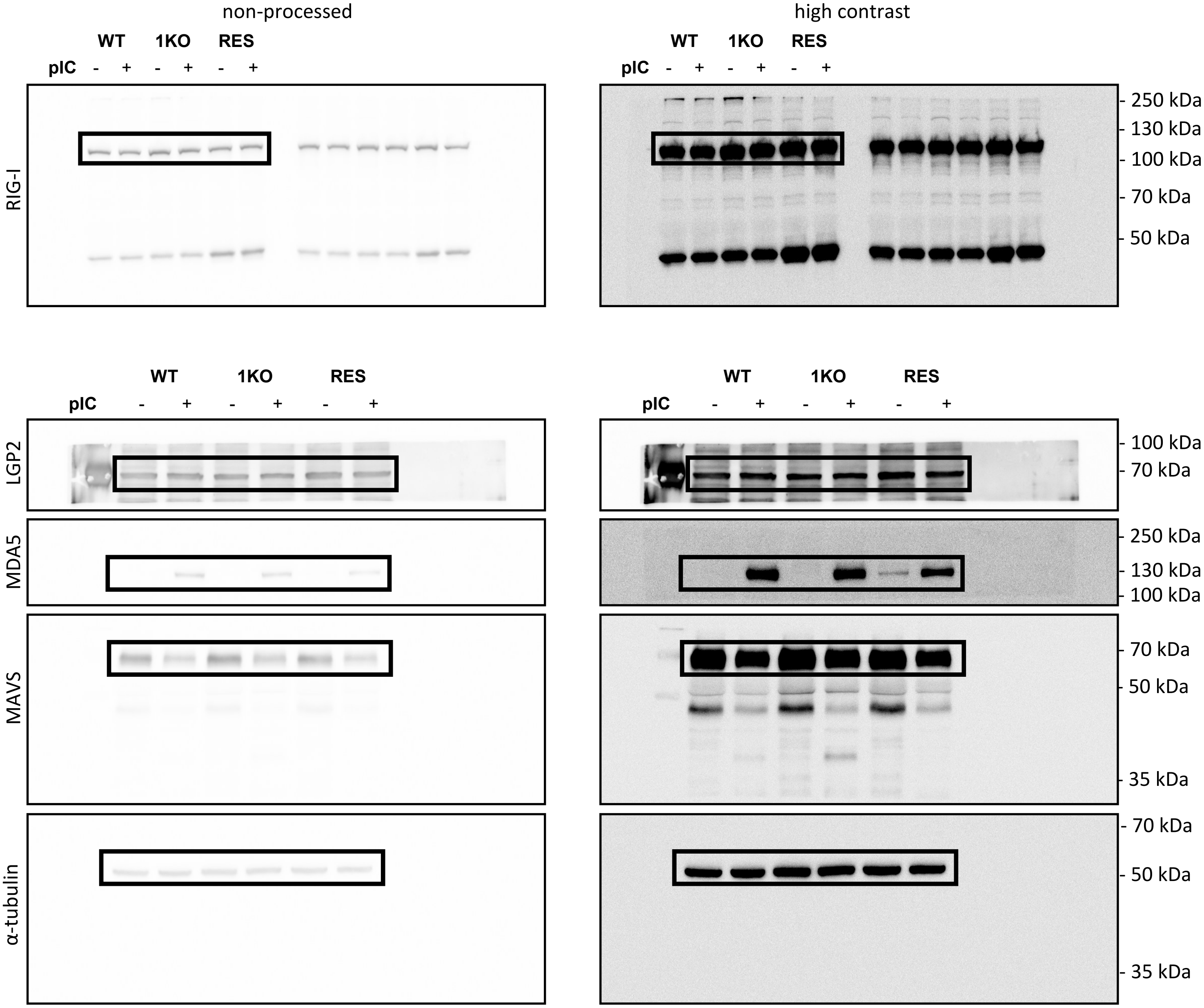
non-processed
high contrast
WT
1KO
RES
pIC
- + - + - +
RIG-I
LGP2
MDA5
MAVS
α-tubulin
250 kDa
130 kDa
100 kDa
70 kDa
50 kDa
100 kDa
70 kDa
250 kDa
130 kDa
100 kDa
70 kDa
50 kDa
35 kDa
70 kDa
50 kDa
35 kDa

Fig. 5C

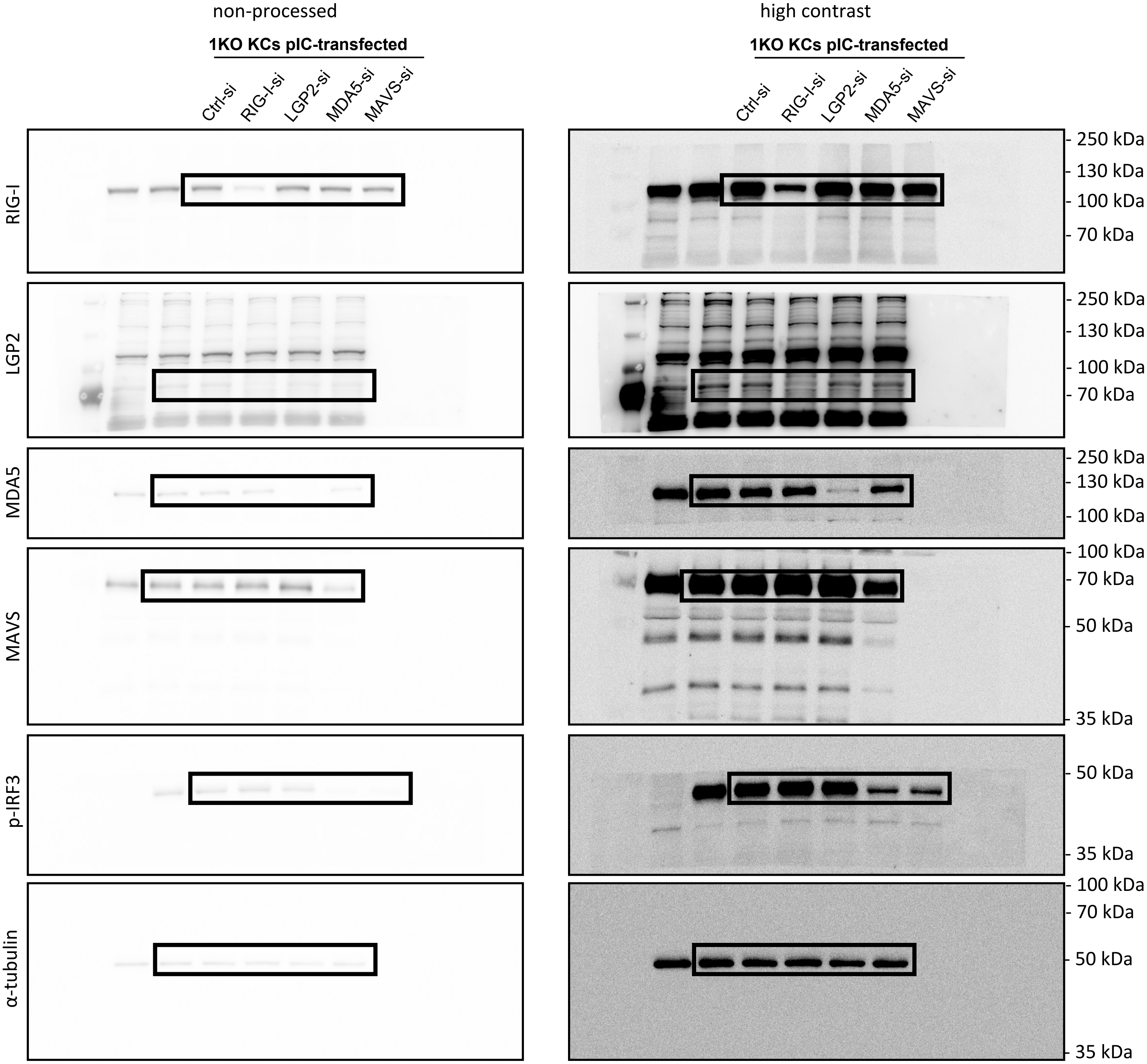
non-processed
high contrast
1KO KCs pIC-transfected
Ctrl-si
RIG-I-si
LGP2-si
MDA5-si
MAVS-si
RIG-I
LGP2
MDA5
MAVS
p-IRF3
α-tubulin
250 kDa
130 kDa
100 kDa
70 kDa
50 kDa
35 kDa

Fig. 6D

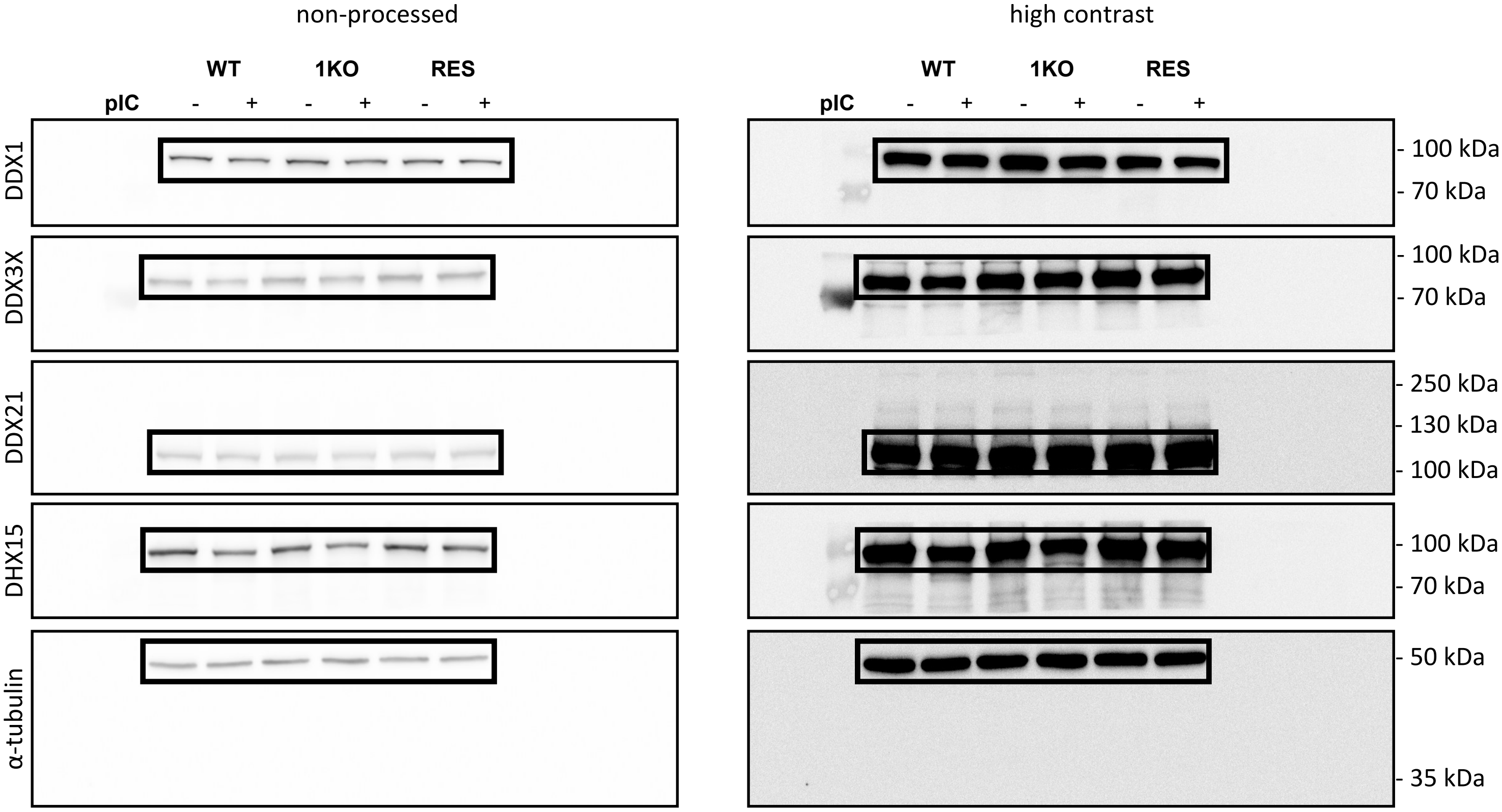
non-processed
high contrast
WT
1KO
RES
pIC
-
+
DDX1
DDX3X
DDX21
DHX15
α-tubulin
100 kDa
70 kDa
250 kDa
130 kDa
50 kDa
35 kDa

Fig. 7A—I

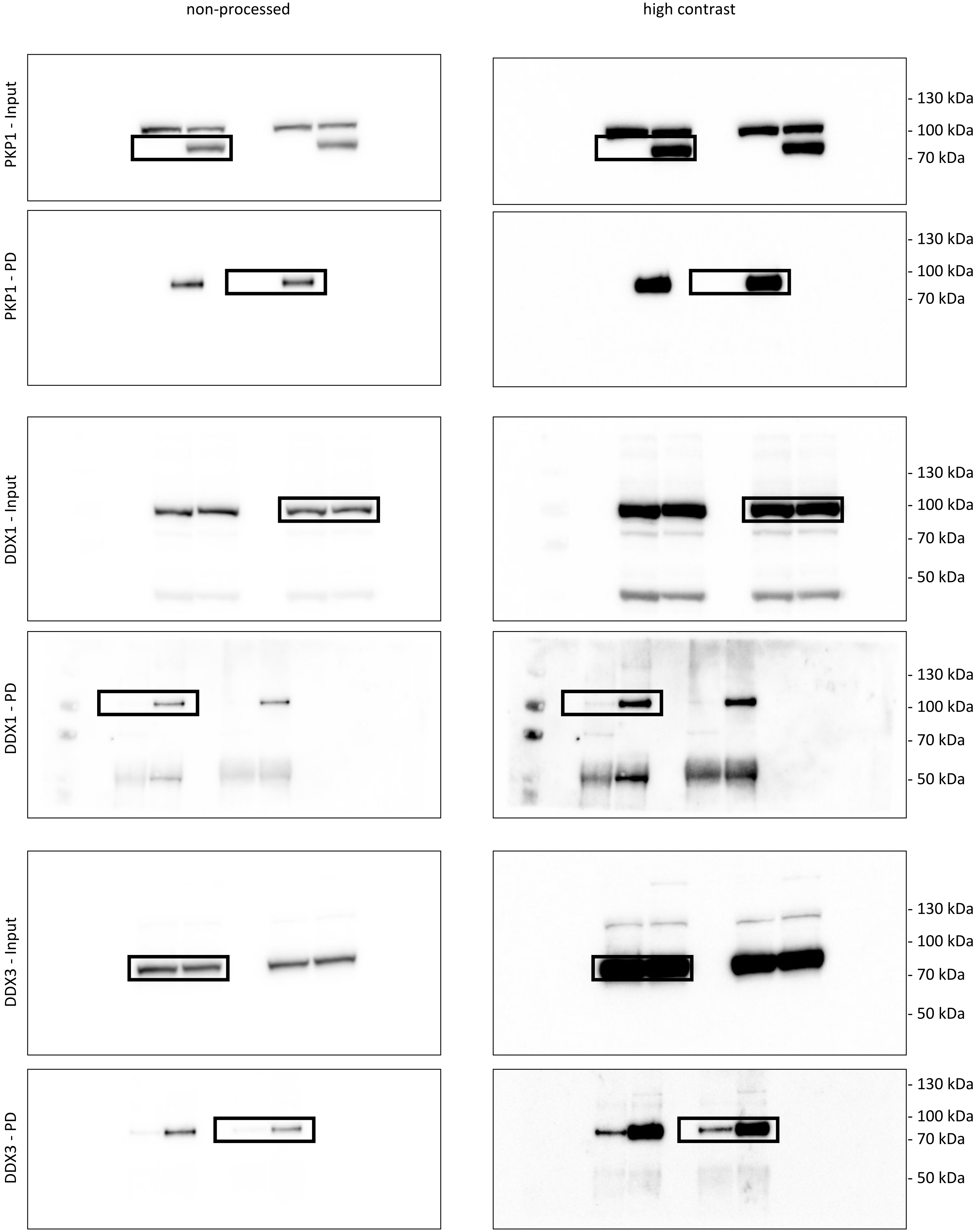
non-processed
high contrast
PKP1 - Input
PKP1 - PD
DDX1 - Input
DDX1 - PD
DDX3 - Input
DDX3 - PD
130 kDa
100 kDa
70 kDa
50 kDa

Fig. 7A - II

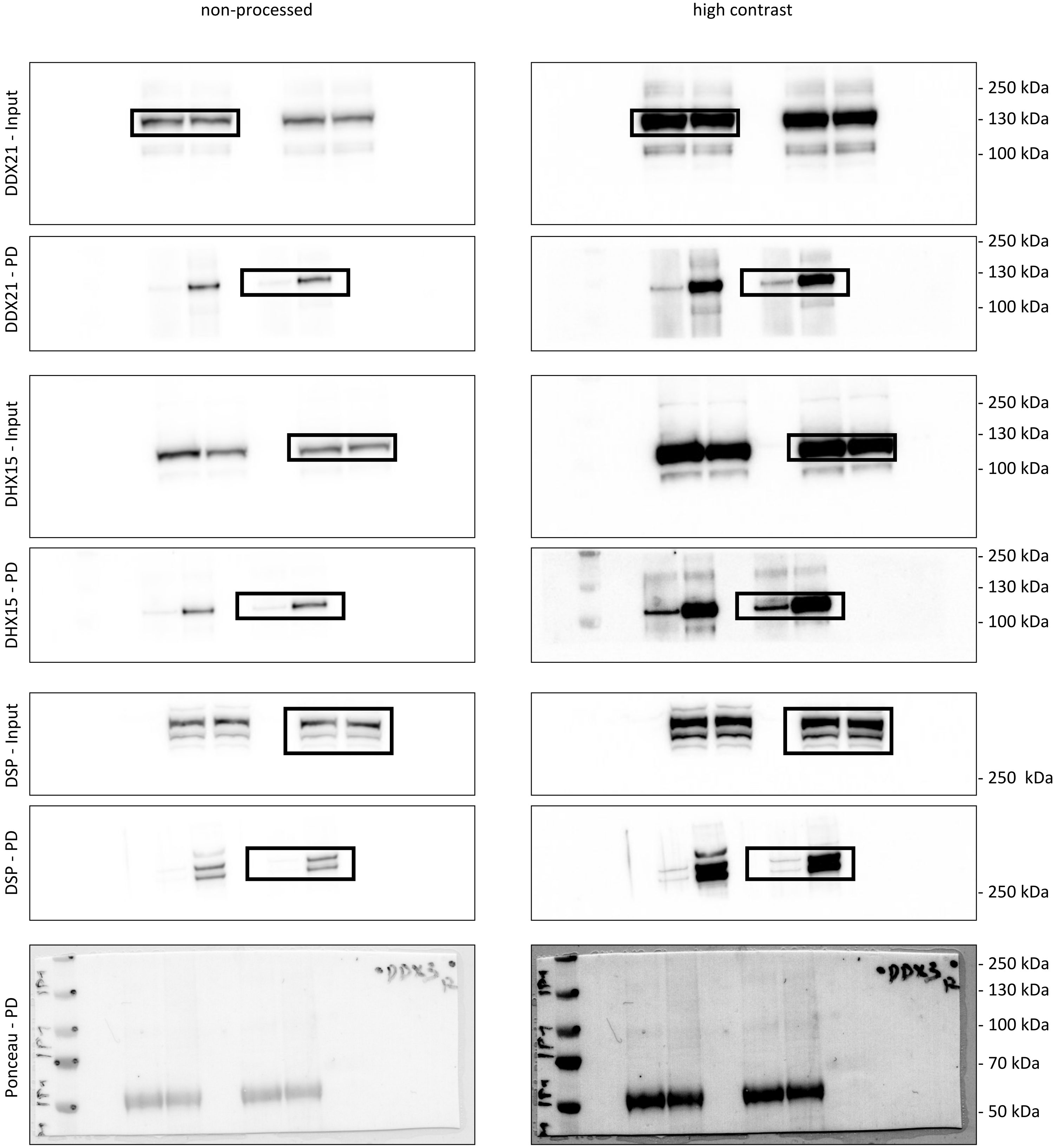

non-processed
high contrast
DDX21 - Input
DDX21 - PD
DHX15 - Input
DHX15 - PD
DSP - Input
DSP - PD
Ponceau - PD
- 250 kDa
- 130 kDa
- 100 kDa
- 250 kDa
- 130 kDa
- 100 kDa
- 250 kDa
- 130 kDa
- 100 kDa
- 250 kDa
- 130 kDa
- 100 kDa
- 250 kDa
- 250 kDa
- 250 kDa
- 130 kDa
- 100 kDa
- 70 kDa
- 50 kDa

Fig. 7B

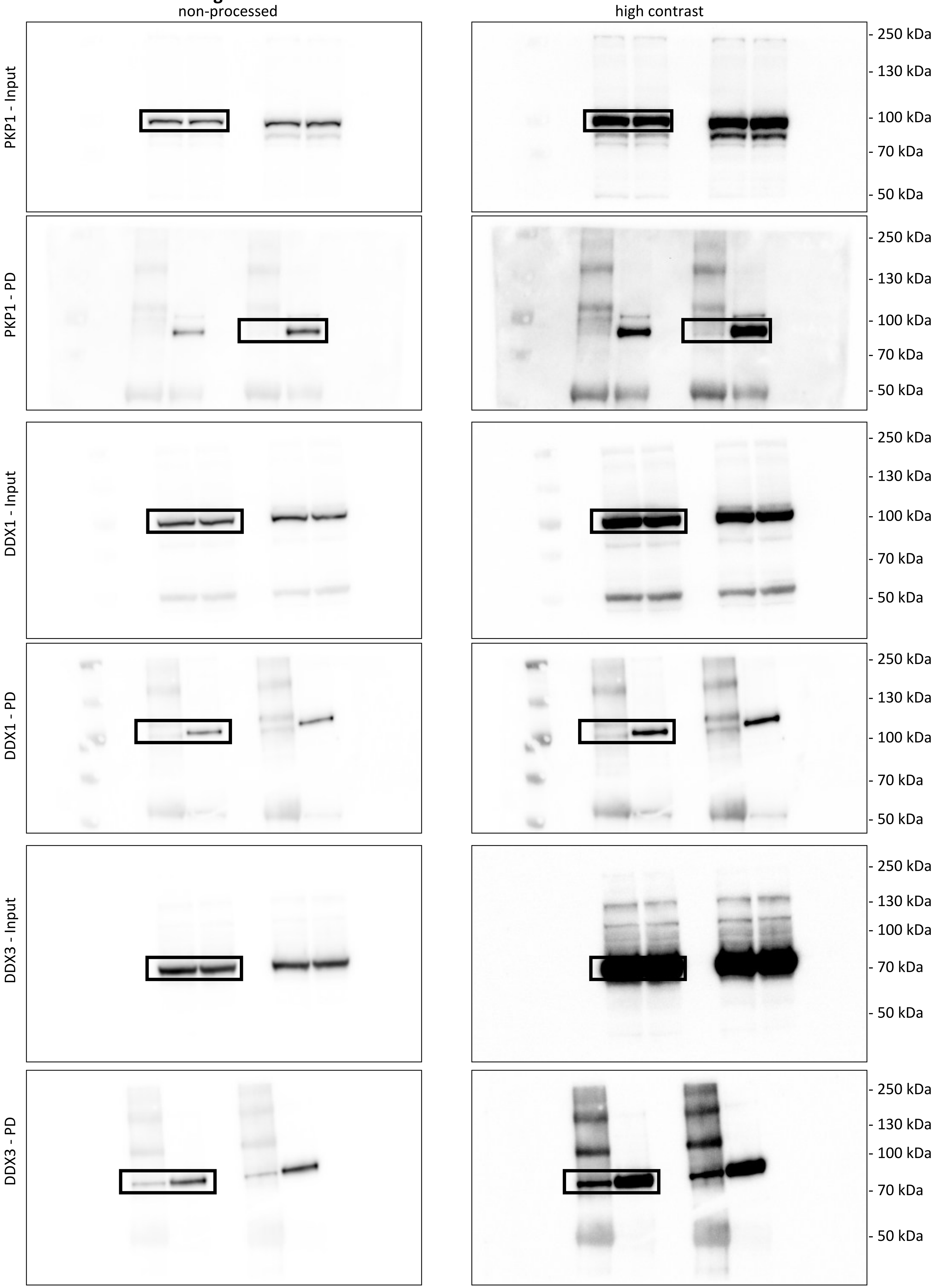
non-processed
high contrast
PKP1 - Input
PKP1 - PD
DDX1 - Input
DDX1 - PD
DDX3 - Input
DDX3 - PD
250 kDa
130 kDa
100 kDa
70 kDa
50 kDa

Fig. 7B—II

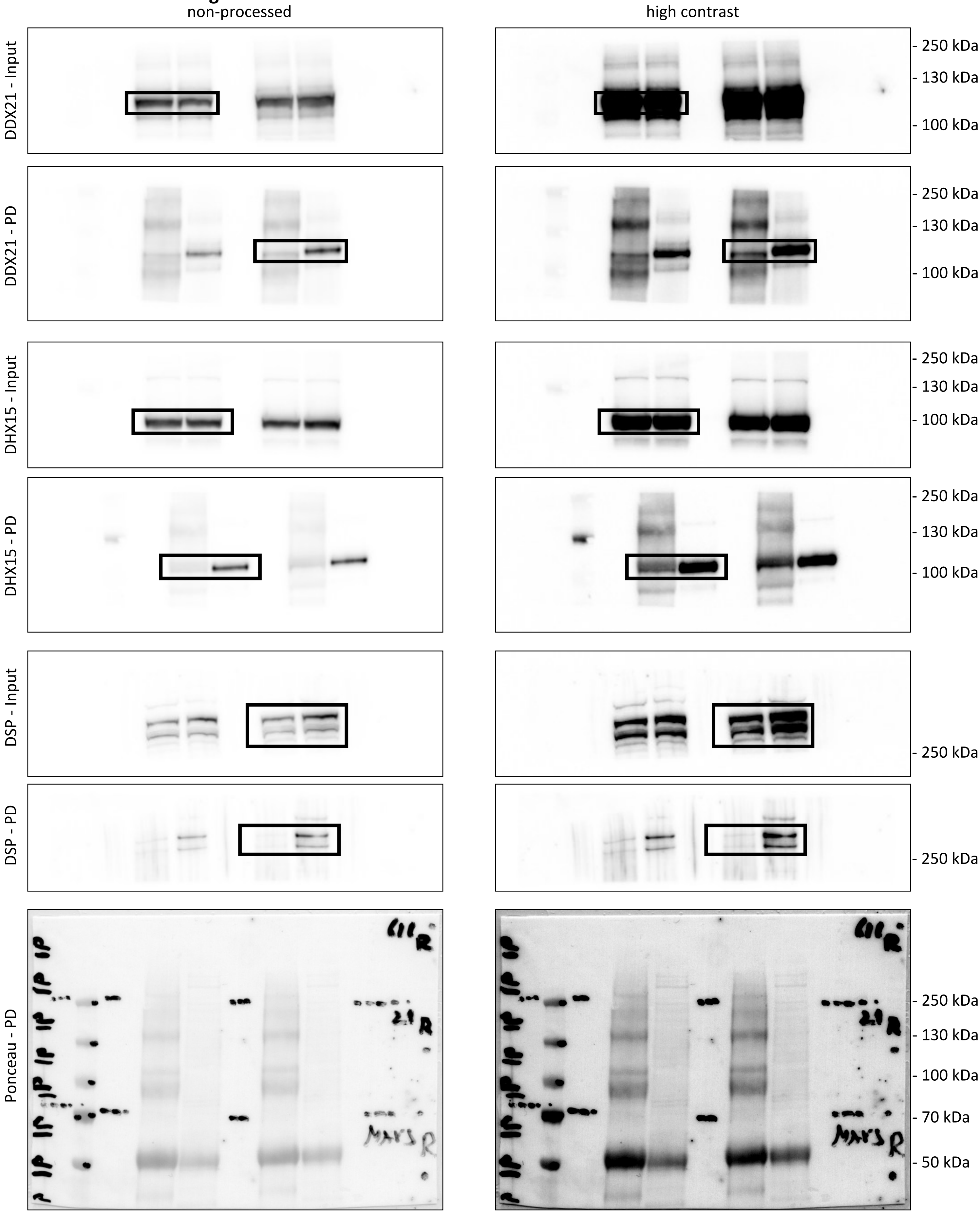
non-processed
high contrast
DDX21 - Input
DDX21 - PD
DHX15 - Input
DHX15 - PD
DSP - Input
DSP - PD
Ponceau - PD
250 kDa
130 kDa
100 kDa
70 kDa
50 kDa

Fig. 7C

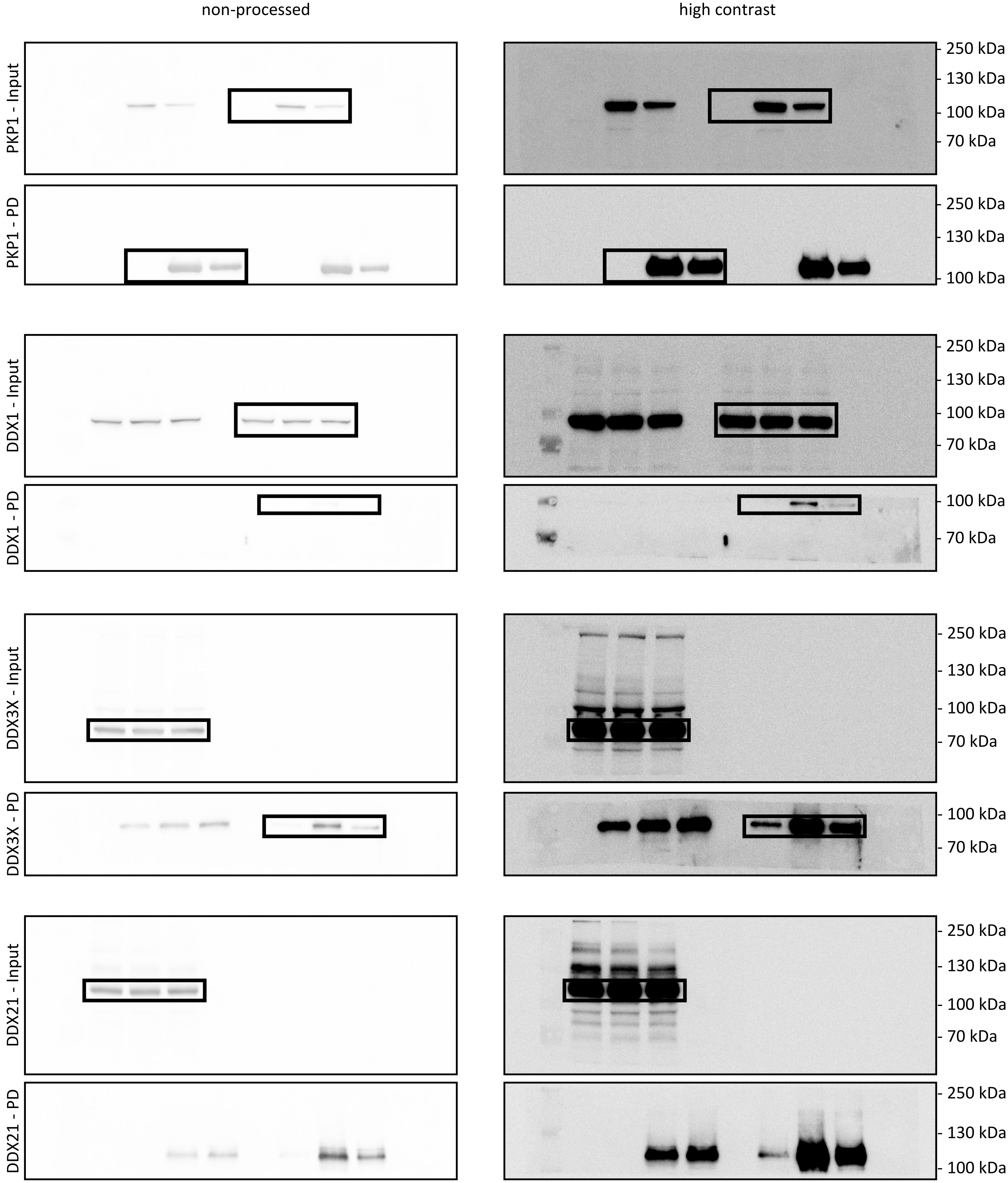

Left lane - PKP1-KO keratinocytes expressing EGFP / Ctrl

Middle lane - PKP1-KO keratinocytes expressing PKP1-WT-EGFP / Ctrl

Right lane - PKP1-KO keratinocytes expressing PKP1-WT-EGFP / pIC-transfected

Fig. 7C

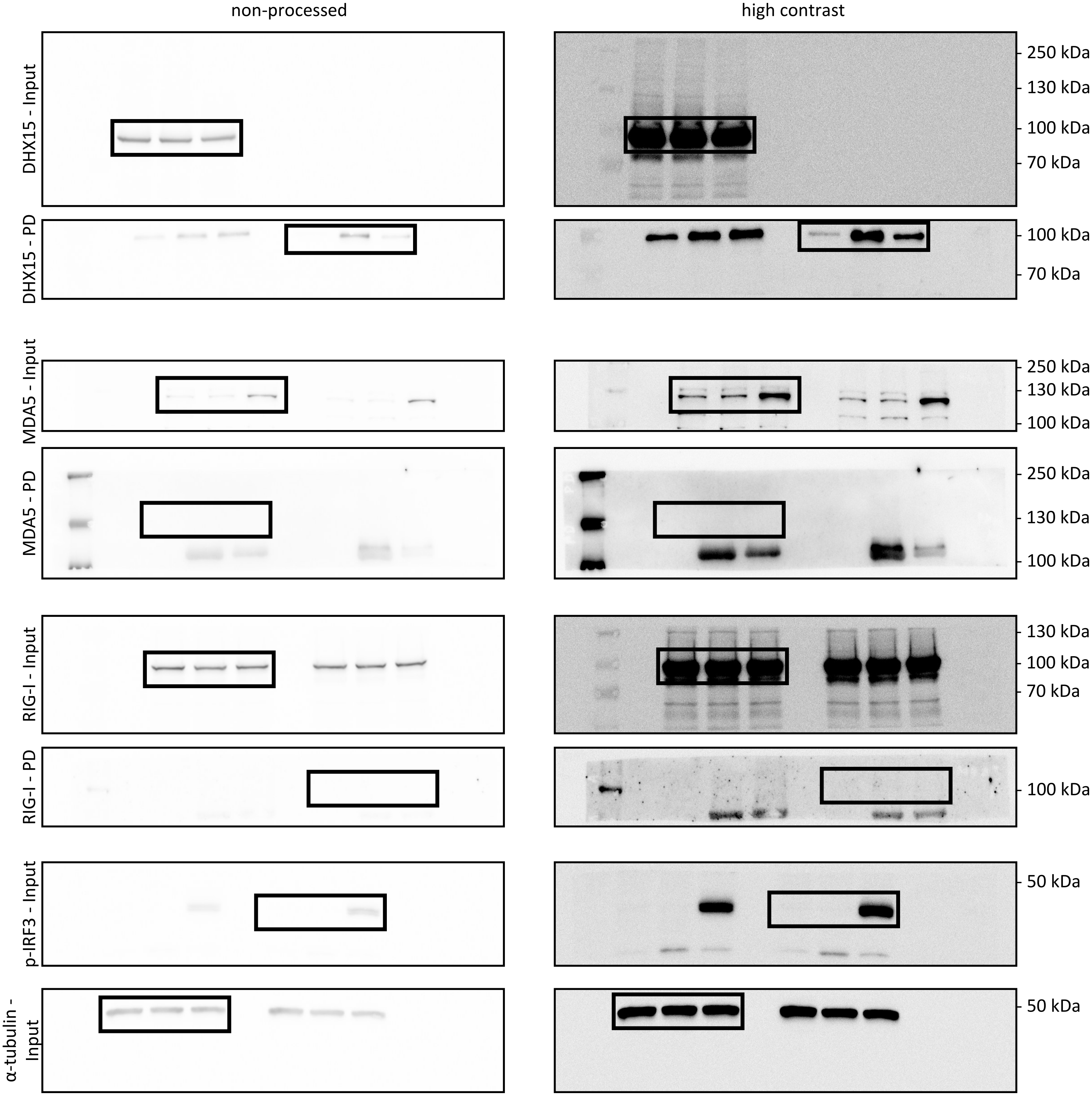

Left lane - PKP1-KO keratinocytes expressing EGFP / Ctrl

Middle lane - PKP1-KO keratinocytes expressing PKP1-WT-EGFP / Ctrl

Right lane - PKP1-KO keratinocytes expressing PKP1-WT-EGFP / pIC-transfected

Fig. 8A

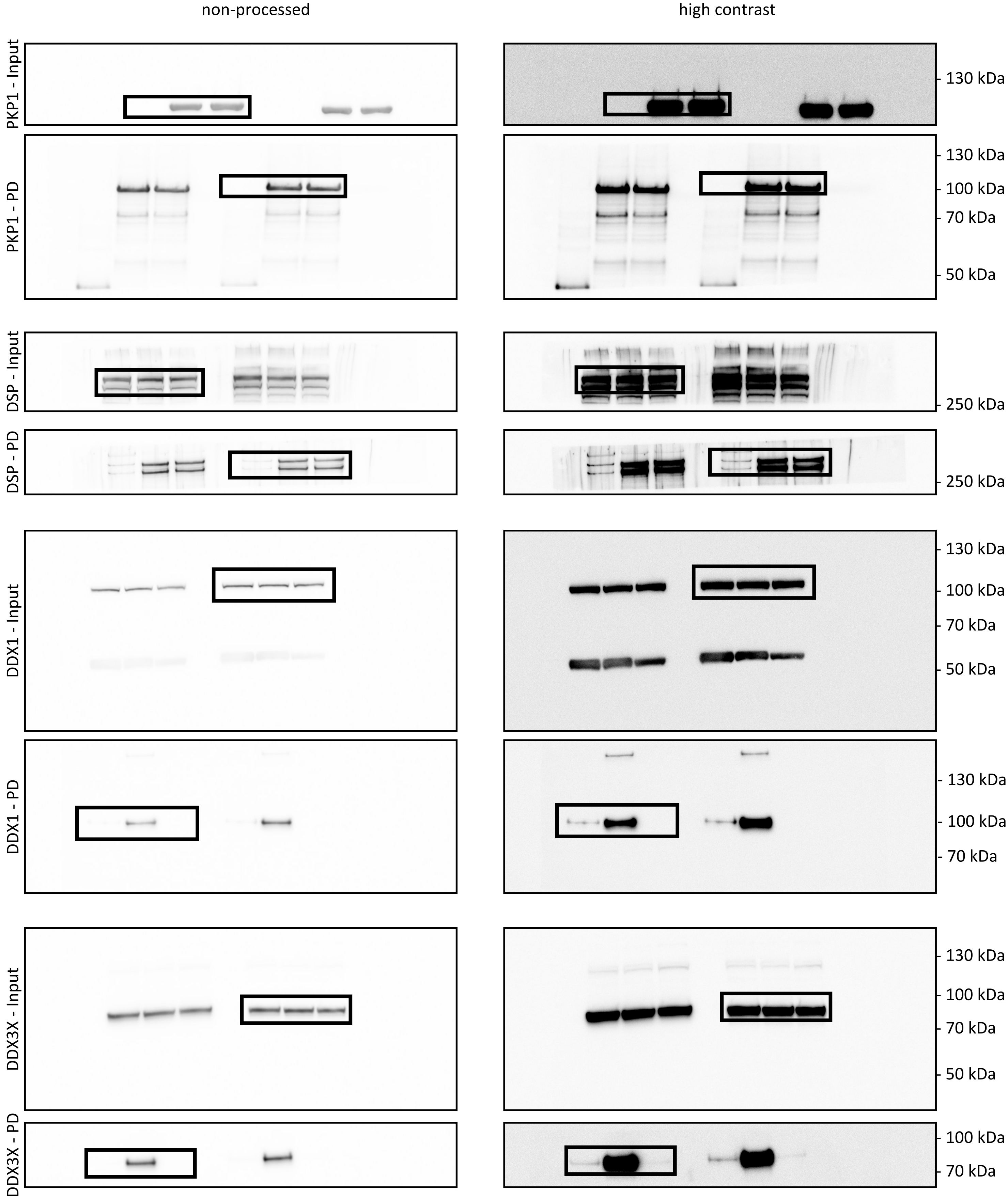

Left lane - PKP1-KO keratinocytes expressing EGFP

Middle lane - PKP1-KO keratinocytes expressing PKP1-WT-EGFP

Right lane - PKP1-KO keratinocytes expressing PKP1-4xA-EGFP

Fig. 8A

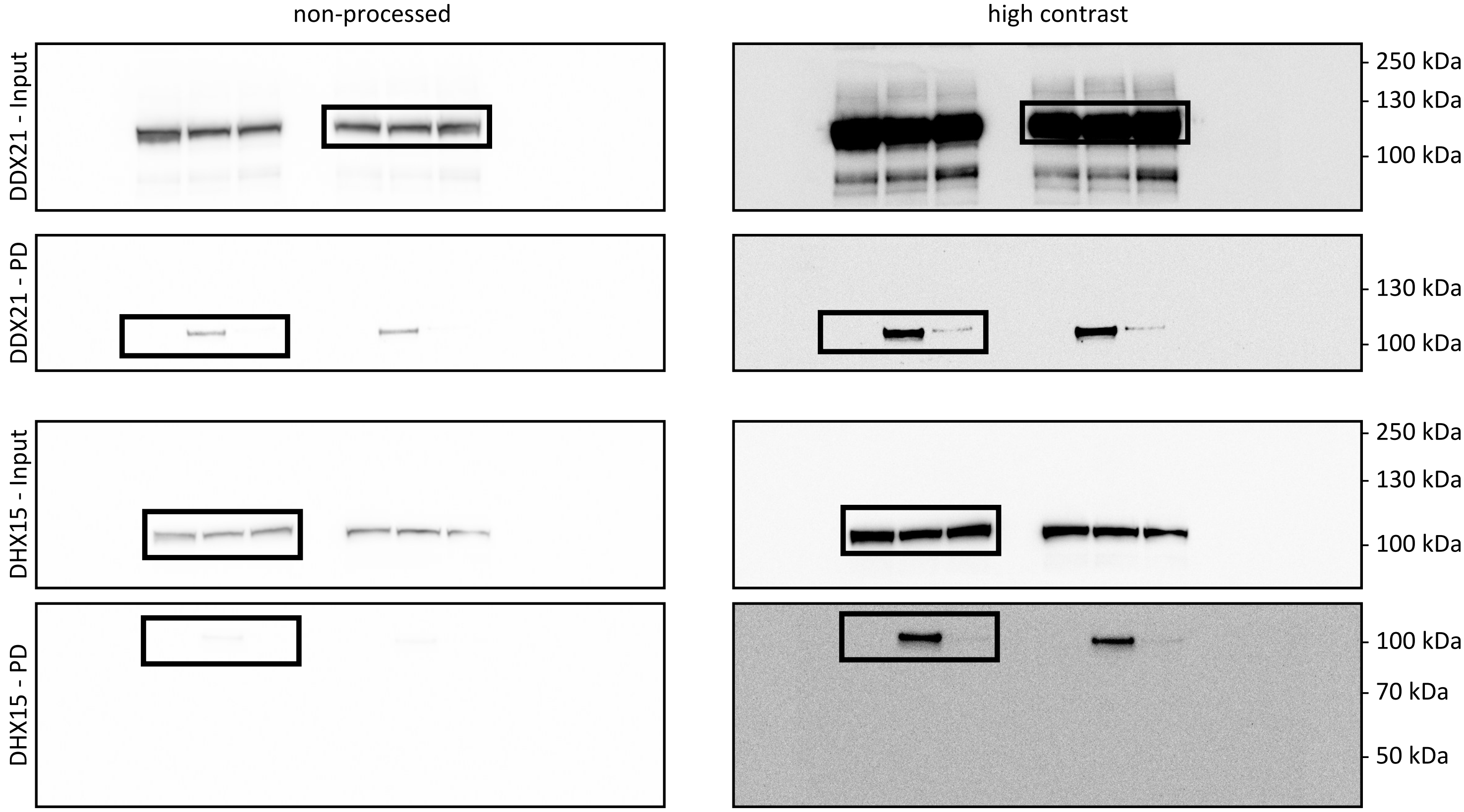

Left lane - PKP1-KO keratinocytes expressing EGFP

Middle lane - PKP1-KO keratinocytes expressing PKP1-WT-EGFP

Right lane - PKP1-KO keratinocytes expressing PKP1-4xA-EGFP

Fig. 8D

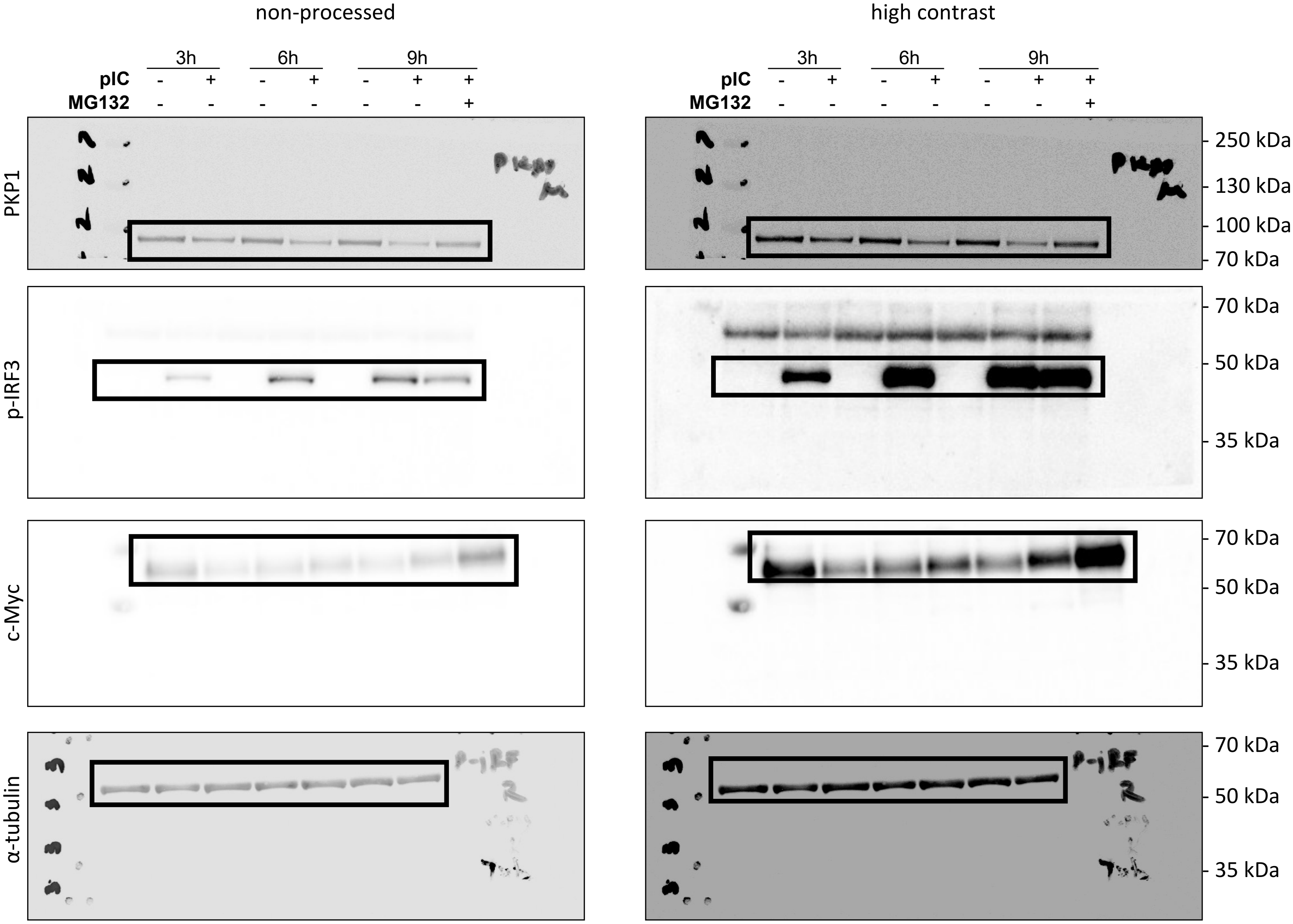
non-processed
high contrast
3h
6h
9h
pIC
MG132
PKP1
p-IRF3
c-Myc
α-tubulin
250 kDa
130 kDa
100 kDa
70 kDa
50 kDa
35 kDa

Fig. S1A

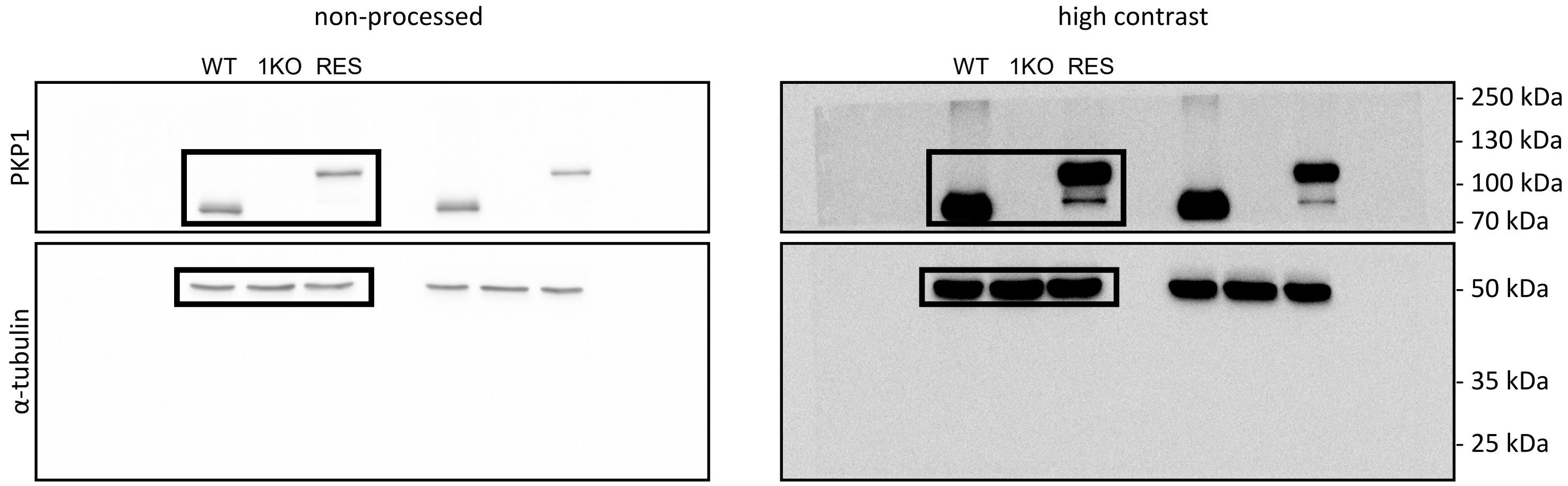

Fig. S1E / S8

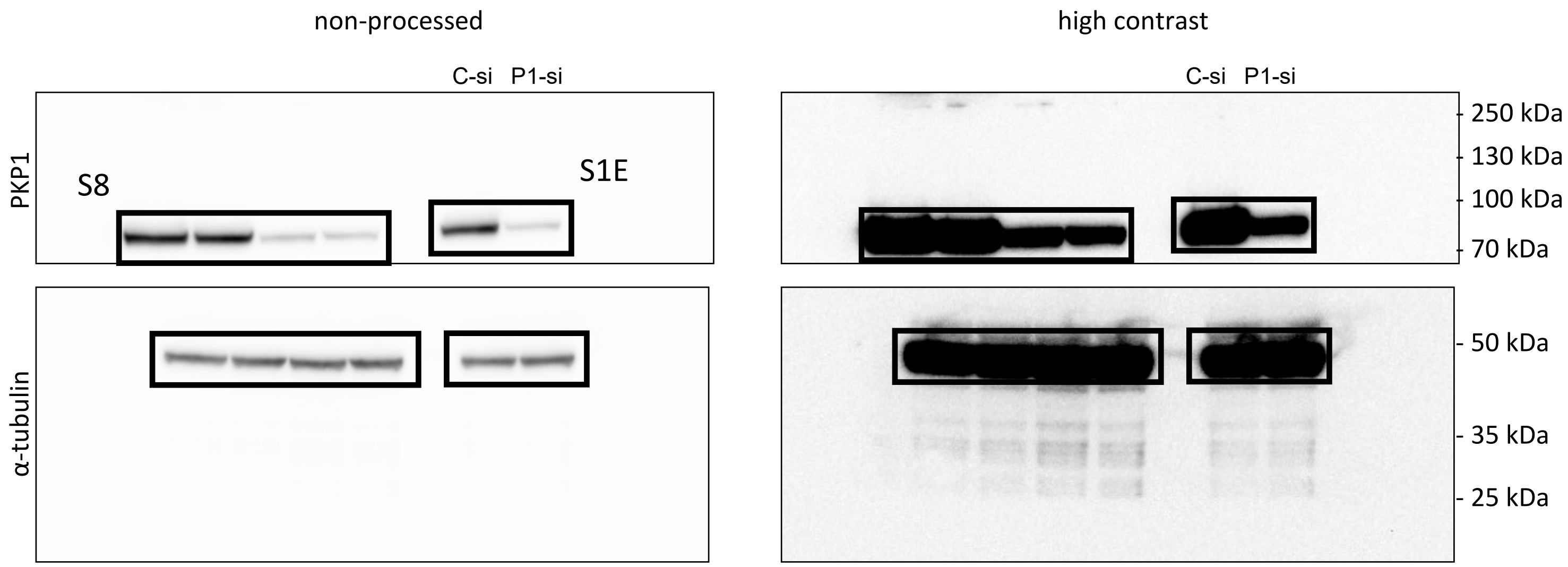

Fig. S3C

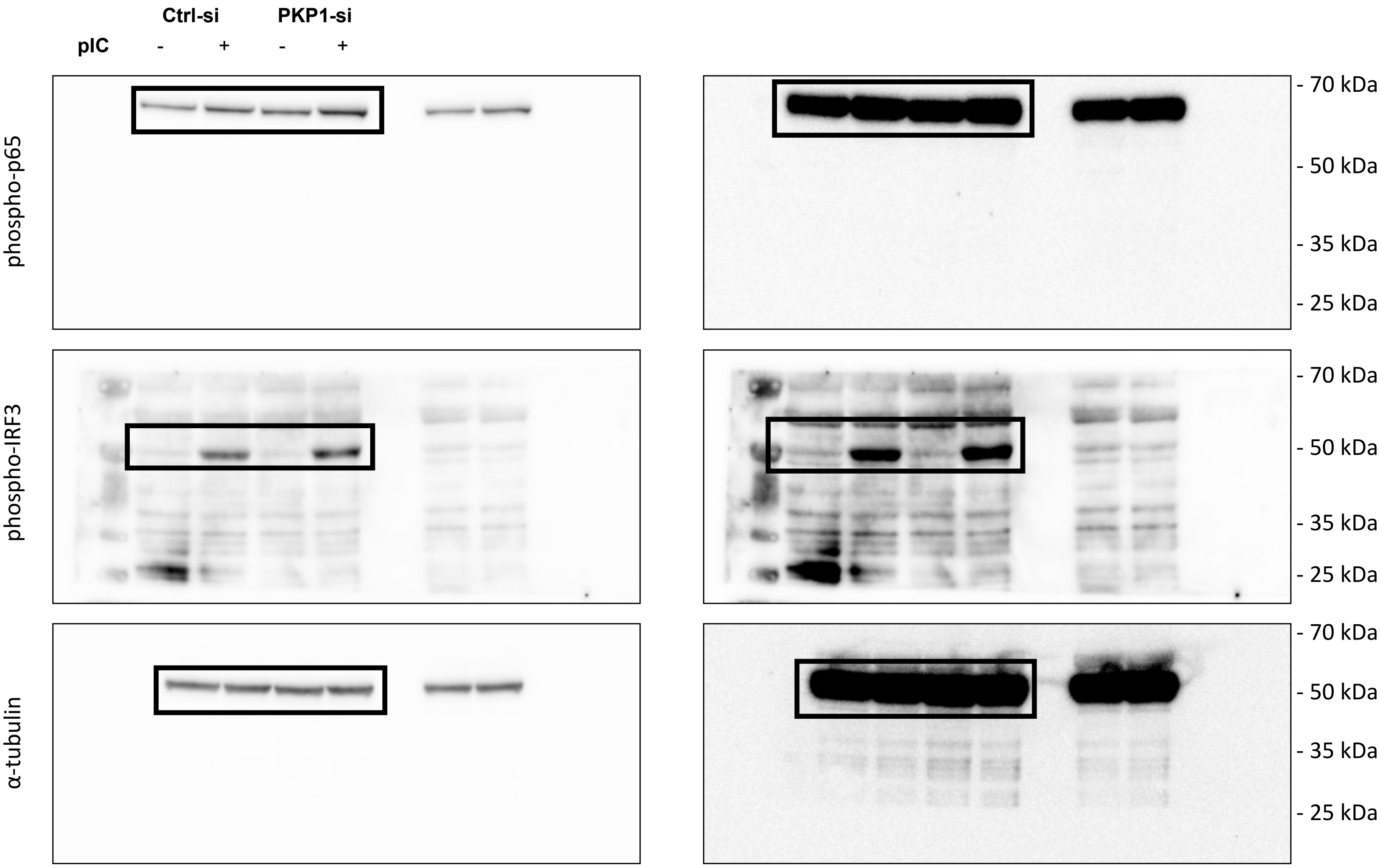
Ctrl-si
PKP1-si
pIC
-
+
-
+
phospho-p65
phospho-IRF3
α-tubulin
- 70 kDa
- 50 kDa
- 35 kDa
- 25 kDa
- 70 kDa
- 50 kDa
- 35 kDa
- 25 kDa
- 70 kDa
- 50 kDa
- 35 kDa
- 25 kDa

Fig. S6B

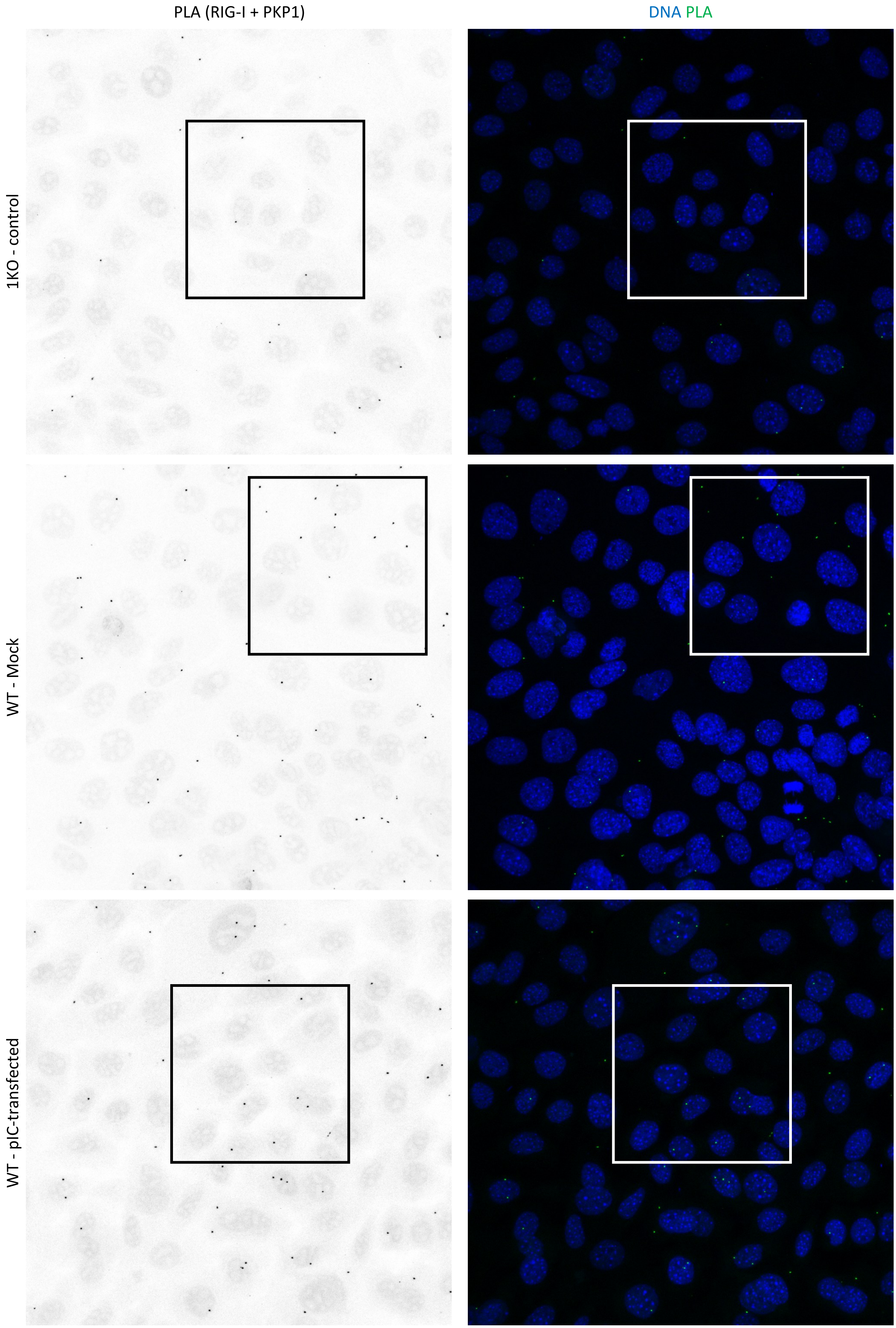

Fig. S6B

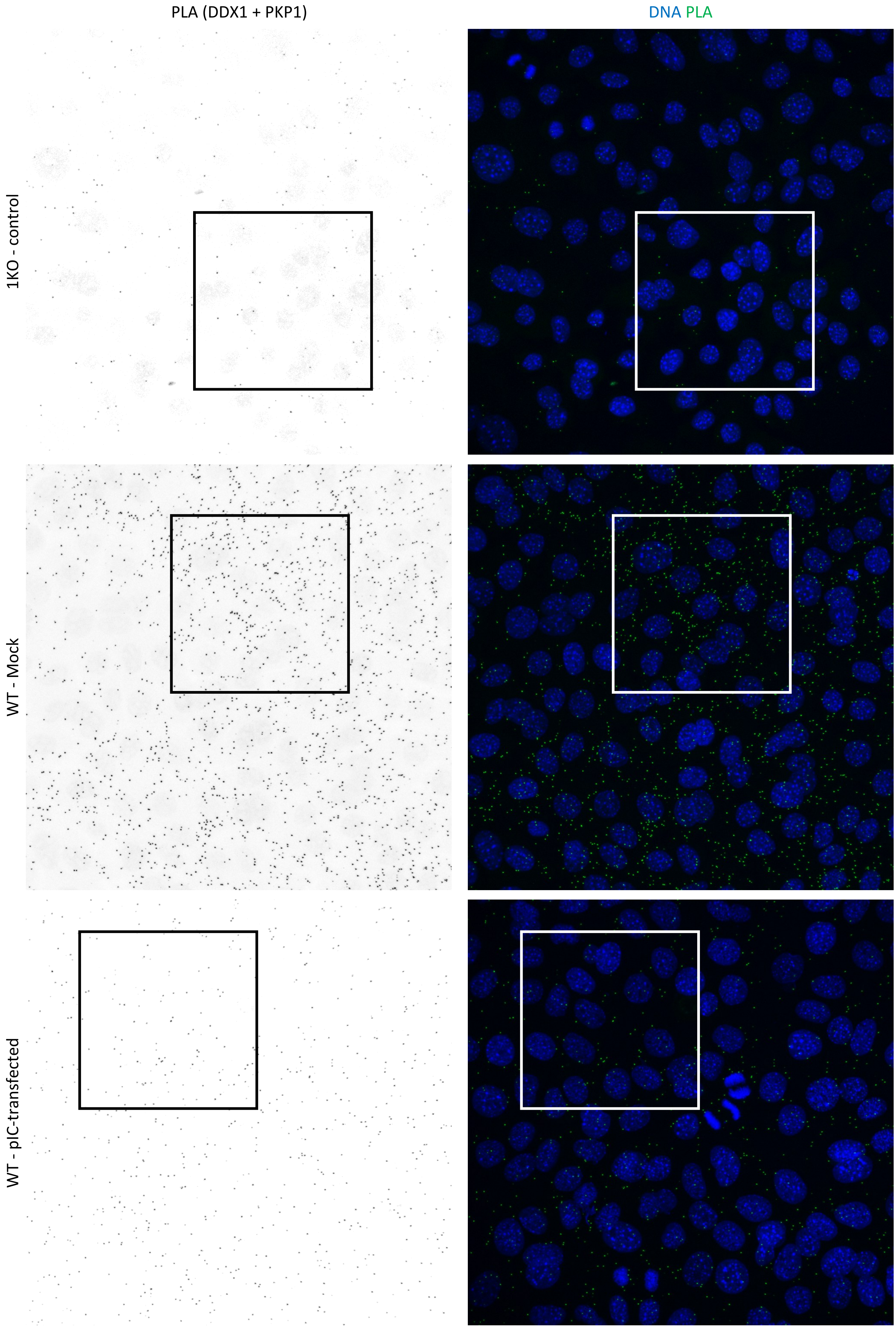

Fig. S6B

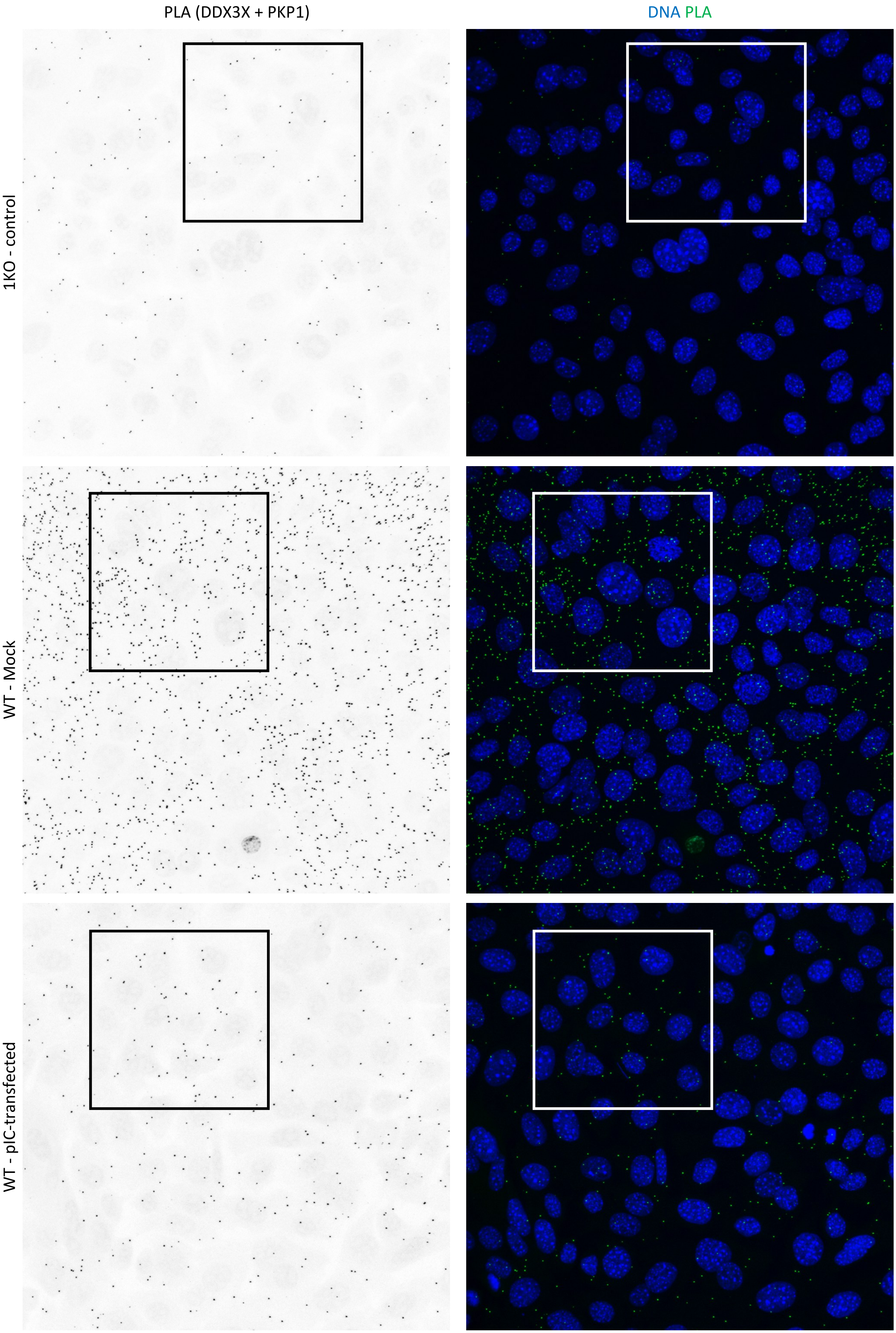
PLA (DDX3X + PKP1)
DNA PLA
1KO - control
WT - Mock
WT - pIC-transfected

Fig. S6B

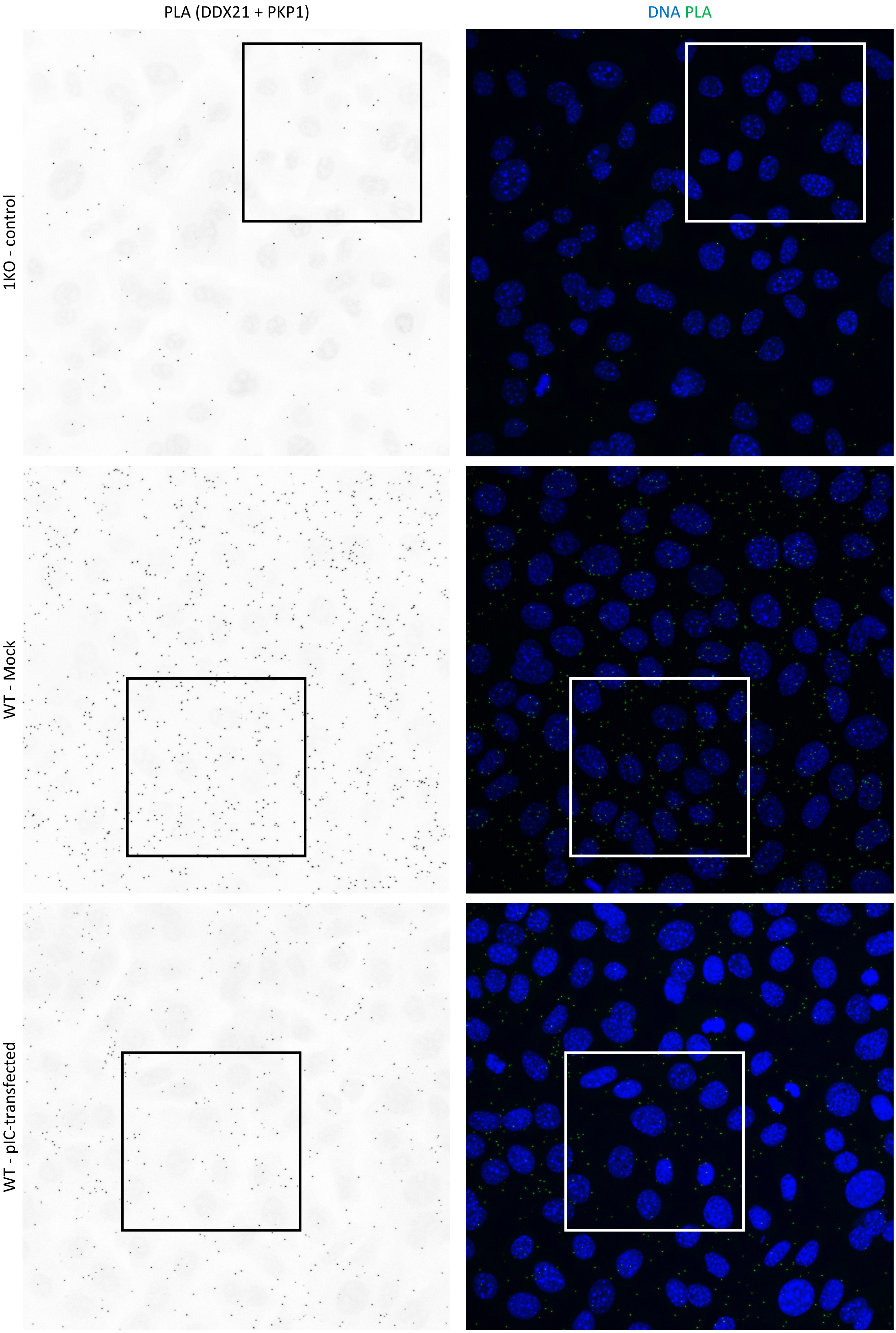
PLA (DDX21 + PKP1)
DNA PLA
1KO - control
WT - Mock
WT - pIC-transfected

Fig. S6B

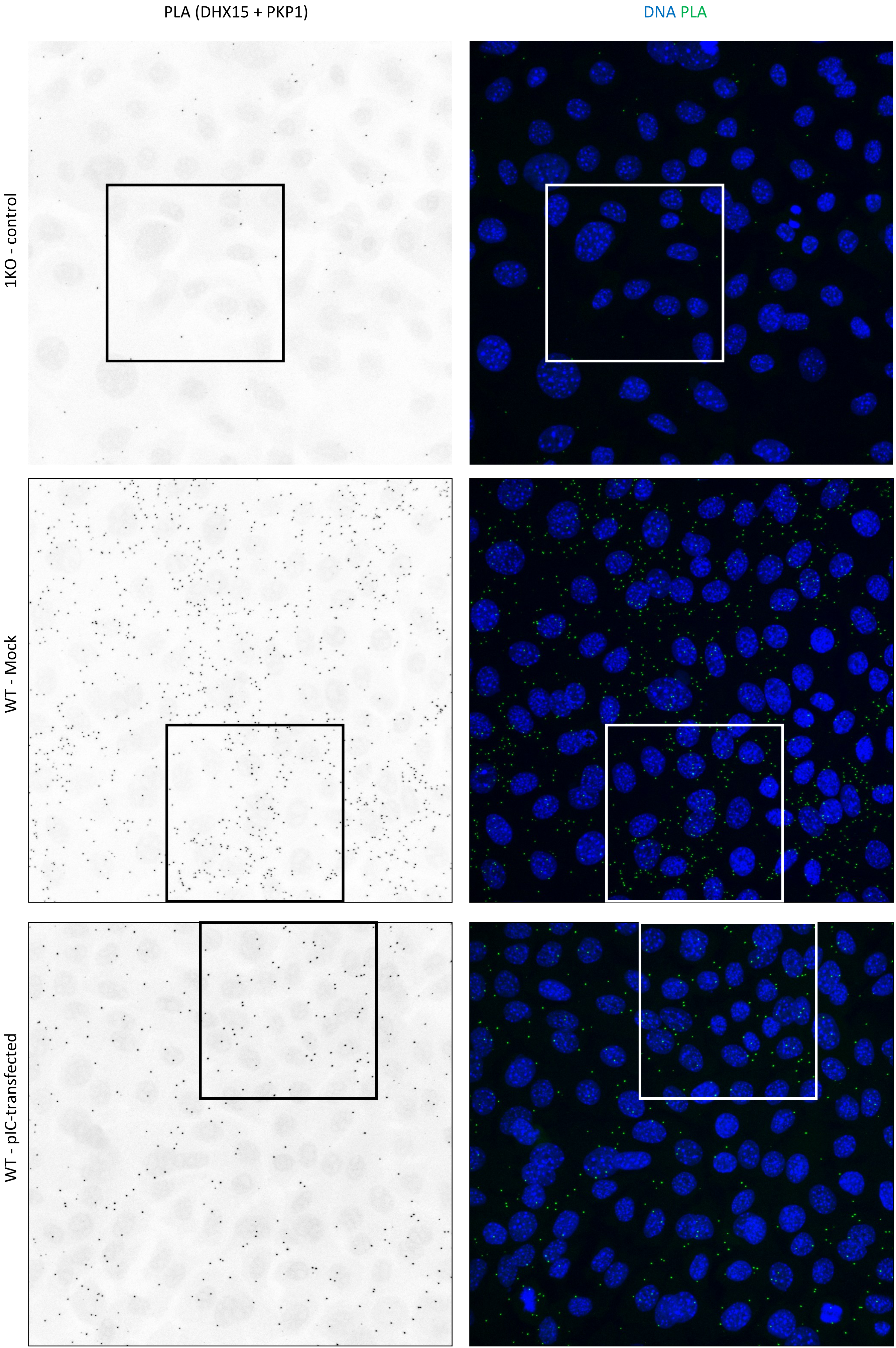
PLA (DHX15 + PKP1)
DNA PLA
1KO - control
WT - Mock
WT - pIC-transfected

Fig. SC

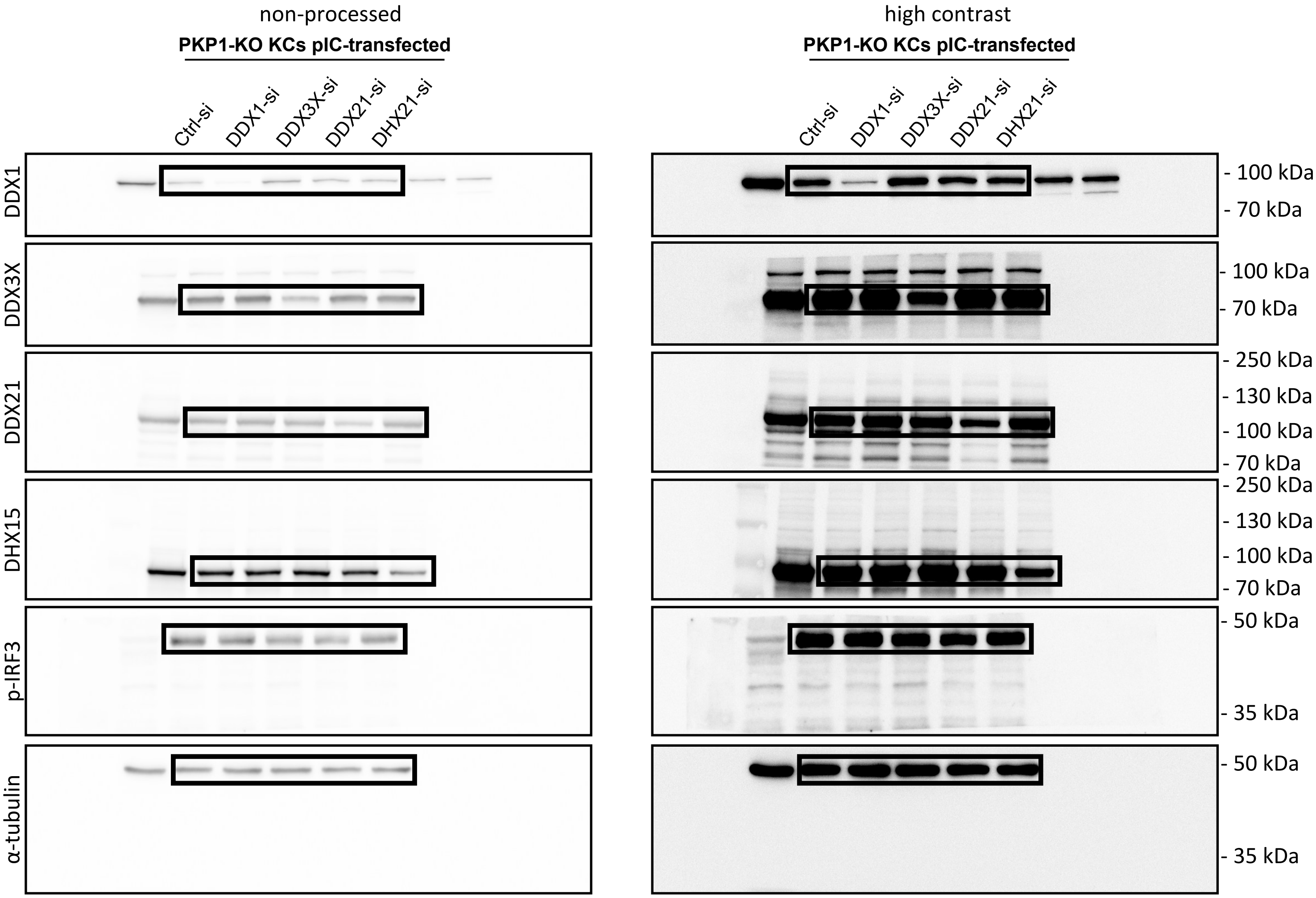
non-processed
PKP1-KO KCs pIC-transfected
Ctrl-si
DDX1-si
DDX3X-si
DDX21-si
DHX21-si
DDX1
DDX3X
DDX21
DHX15
p-IRF3
α-tubulin
high contrast
PKP1-KO KCs pIC-transfected
Ctrl-si
DDX1-si
DDX3X-si
DDX21-si
DHX21-si
- 100 kDa
- 70 kDa
- 100 kDa
- 70 kDa
- 250 kDa
- 130 kDa
- 100 kDa
- 70 kDa
- 250 kDa
- 130 kDa
- 100 kDa
- 70 kDa
- 50 kDa
- 35 kDa
- 50 kDa
- 35 kDa

Fig. S6B

MKC - PKP1-WT-EGFP

MKC - PKP1-4xA-EGFP

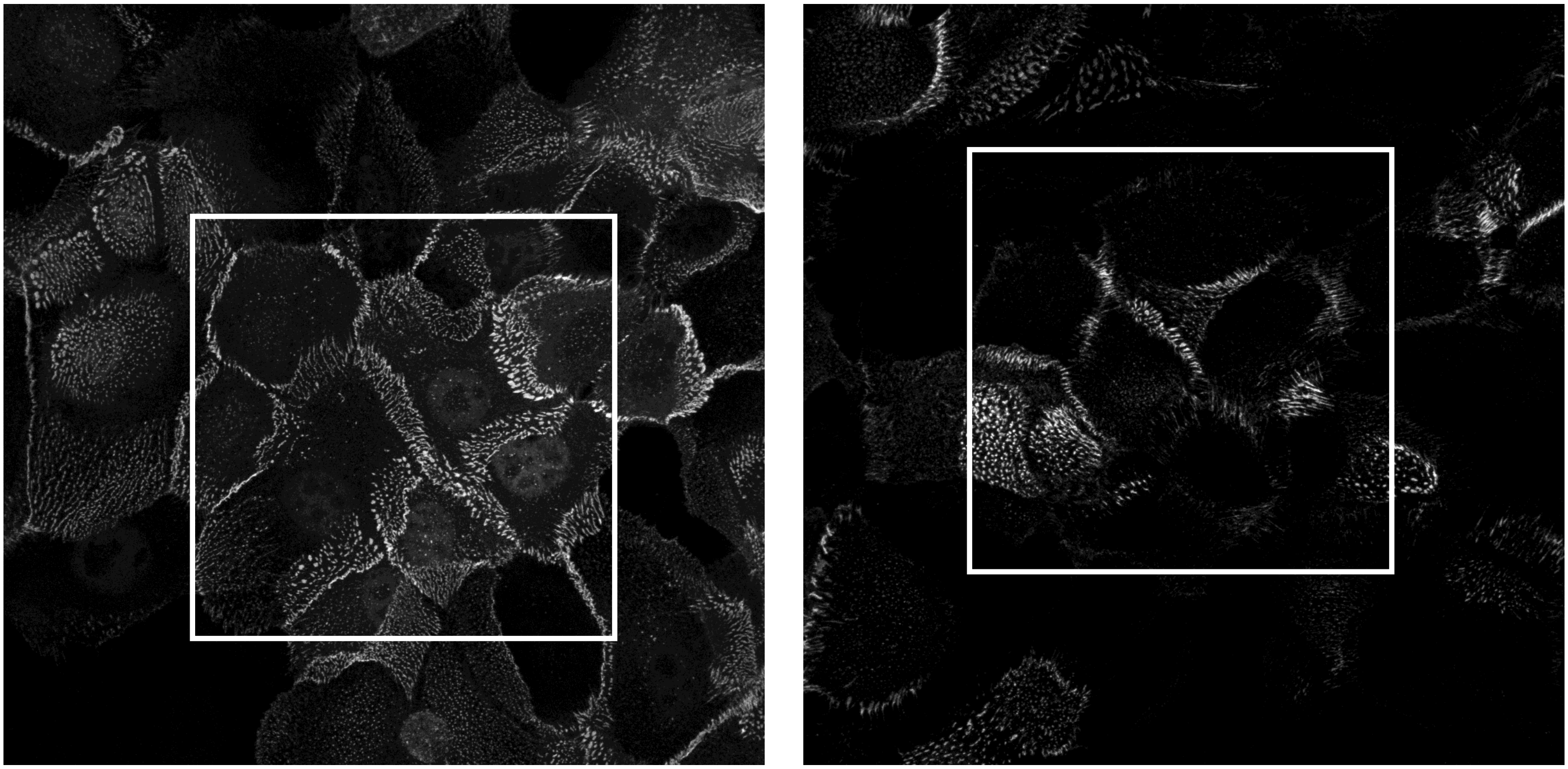

Supplement: Supplementary file 2 [file 41423_2026_1462_MOESM2_ESM.pdf]
